# TimeNorm: a novel normalization method for time course microbiome data

**DOI:** 10.3389/fgene.2024.1417533

**Published:** 2024-09-24

**Authors:** Qianwen Luo, Meng Lu, Hamza Butt, Nicholas Lytal, Ruofei Du, Hongmei Jiang, Lingling An

**Affiliations:** ^1^ Department of Biosystems Engineering, University of Arizona, Tucson, AZ, United States; ^2^ Graduate Interdisciplinary Program in Statistics and Data Science, University of Arizona, Tucson, AZ, United States; ^3^ Department of Biostatistics and Epidemiology, University of Arizona, Tucson, AZ, United States; ^4^ Department of Mathematics and Statistics, California State University at Chico, Chico, CA, United States; ^5^ Department of Biostatistics, University of Arkansas for Medical Sciences, Little Rock, AR, United States; ^6^ Department of Statistics and Data Science, Northwestern University, Evanston, IL, United States

**Keywords:** microbiome, metagenomics, normalization, time-course, dominant features, longitudinal

## Abstract

Metagenomic time-course studies provide valuable insights into the dynamics of microbial systems and have become increasingly popular alongside the reduction in costs of next-generation sequencing technologies. Normalization is a common but critical preprocessing step before proceeding with downstream analysis. To the best of our knowledge, currently there is no reported method to appropriately normalize microbial time-series data. We propose TimeNorm, a novel normalization method that considers the compositional property and time dependency in time-course microbiome data. It is the first method designed for normalizing time-series data within the same time point (intra-time normalization) and across time points (bridge normalization), separately. Intra-time normalization normalizes microbial samples under the same condition based on common dominant features. Bridge normalization detects and utilizes a group of most stable features across two adjacent time points for normalization. Through comprehensive simulation studies and application to a real study, we demonstrate that TimeNorm outperforms existing normalization methods and boosts the power of downstream differential abundance analysis.

## 1 Introduction

Microbial time-series studies offer insight into investigating microbial system dynamics. The human microbiota is highly dynamic and closely associated with various health factors (Gerber, 2014; David et al., 2014), including diet (Zmora et al., 2019; Kashtanova et al., 2016), infectious diseases (Zhang et al., 2015), and palliative measures such as antibiotic regimens (Becattini et al., 2016) and cancer treatments (Roy and Trinchieri, 2017). Longitudinal studies are characterized by multiple observations from the same individual over regular/irregular time intervals and are invaluable for understanding the dynamics of host-microbe associations. These studies prompt several research questions such as: Which microbes have differential longitudinal patterns between conditions? Does the microbiome recover to its original state if it is altered through stimuli such as infections? What are the starting and ending time points when the differences arise? How do microbes associate with the host over time?

Metagenomics relies on high-throughput sequencing technologies to yield genomic and taxonomic information of microbes in a sample. Two approaches are commonly used to study microbial communities: 16S rRNA sequencing and whole-genome shotgun (WGS) sequencing. 16S rRNA genes are highly conserved regions in bacterial ribosomes and hence serve as a useful phylogenetic marker. WGS sequences all given genomic DNA from a sample. Time course microbiome studies usually prefer 16S rRNA sequencing due to its low cost. Numerous bioinformatics pipelines have been developed for processing metagenomic sequencing data and subsequently quantifying them through microbial count/abundance tables. Count data from different samples are not directly comparable due to differences in library sizes, which are highly variable and constrained by sequencing depth and technology. This total count constraint introduces strong dependencies between taxa abundances, particularly magnifying the differences in relative abundances between taxa when one taxon is highly expressed in a sample (Calle, 2019). The sparse and high-dimensional nature of microbial count data (Knight et al., 2018) requires the development of appropriate statistical tools capable of accommodating those data characteristics. Considering the challenges in obtaining the same number of sequencing reads for each sample, normalization is imperative to correct or eliminate bias introduced by variable library sizes and make samples comparable between conditions or time points.

Various normalization methods have been developed for sequencing-based microbiome data. Two types of commonly used normalization methods are rarefying and scaling. Rarefying involves subsampling the original sample to even depth without replacement, which impacts alpha and beta diversities (McMurdie and Holmes, 2014). Scaling, on the other hand, is based on dividing the observed abundance of the feature by a scaling factor or normalization factor to eliminate biases arising from unequal library size. Total Sum Scaling (TSS) or total count (TC) normalization method scales counts into proportions, e.g., the count for each taxon is divided by the library size, which is not robust to outliers. Outliers can overestimate the library size and not reflect the true abundance. Furthermore, counts would be skewed by a few relatively abundant features. An ideal normalization method should be robust against outliers. Cumulative sum scaling (CSS) is a normalization method developed for microbiome sequencing data (Paulson et al., 2013), which calculates the normalization factor as a sum over a subset of features. CSS assumes that the count distribution is approximately equal to a certain quantile. This normalization factor is implemented in the metagenomeSeq Bioconductor package.

Several normalization methods developed for RNA-seq data (Dillies et al., 2013; Evans et al., 2018) are also applicable to microbiome data. Trimmed mean of M-values (TMM) (Robinson and Oshlack, 2010), is a method implemented in the edgeR Bioconductor package. TMM assumes that most features are not differentially expressed. The TMM scaling factor is calculated as the weighted mean of log-fold changes between each pair of samples, excluding the extreme log-fold changes and the most extreme counts. Normalized counts are calculated by dividing raw counts by scaling factors. Another well-known normalization method developed for RNA-seq data is Relative Log Expression (RLE) (Anders and Huber, 2010). RLE calculates the geometric means of all features and the median ratio of each sample to the median library. The median ratio is the scaling factor, which is specified in edgeR.

Geometric mean of pair ratios (GMPR) is a recently proposed normalization method that considers zero-inflation (Chen L. et al., 2018) in microbiome data and is built upon RLE normalization. It reverses the order of scaling factor calculation steps in RLE to deal with excess zeros, which commonly occur in microbiome sequencing data. It first calculates the median count ratio of non-zero counts between samples, and then calculates the size factor of a given sample. RioNorm2, another recently developed normalization technique for microbiome data (Ma et al., 2020), assumes that there exists a subset of features which are relatively invariant across samples and conditions. RioNorm2 calculates the pairwise dissimilarity among the top abundant features and then finds a subset of relatively invariant features using network analysis. The normalization size factor is the total count of this identified subset of features.

These existing normalization methods are for non-time series data. In this paper, we develop a novel normalization method, TimeNorm, for microbiome time-course data taking into account of the data characteristics such as compositional property and correlated measurements over time. This new method uses different strategies when normalizing samples under the same condition and across time points. Simulation studies and real data analysis are used to demonstrate the good performance of TimeNorm.

## 2 Methods

### 2.1 An overview of TimeNorm

**FIGURE 1:**
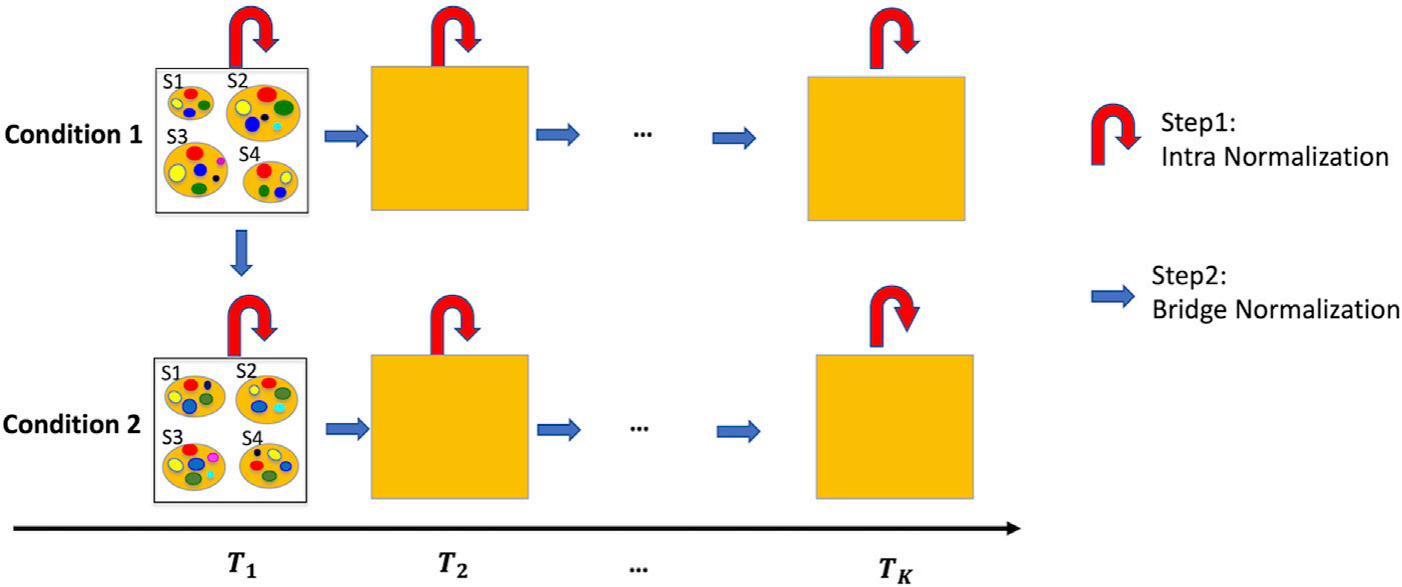
Illustration of the normalization method, TimeNorm, for time series data under two conditions with four subjects per condition as an example. Intra-time normalization normalizes the microbial samples under the same condition and at the same time point according to the common dominant features (red curved arrows); Bridge normalization normalizes the microbial samples at the initial time point under condition 1 and condition 2. Then within each condition, it normalizes the samples at two adjacent time points (blue arrows). To better view the sample details in each square/time point, we display the samples at the initial time point as an example. S1 – S4 are four samples; small colorful bubbles represent different microbiome species/features. The dominant features in this example are red, green, yellow, and blue features (see the details in Sections 2.2, 2.3 for normalization).

Time-series microbiome studies, characterized by multiple time-points and multiple samples at each time point, are additionally limited to under-sampling and possible batch effects between time-points. Figure 1 provides a roadmap for the proposed normalization method –TimeNorm which includes intra-time normalization and Bridge normalization, for time series microbiome data under two conditions such as two different treatments. First, at each time point, Intra-time normalization is performed for the microbial samples under the same condition using the common dominant features (i.e., features which appear in all samples). Next, Bridge normalization is then conducted for the comparison of (1) two conditions at the initial time point and (2) two adjacent time points under the same condition, sequentially. Specifically, we make two assumptions. The first assumption is that the majority of features are not differentially abundant at the initial timepoint between the two conditions. This simplified assumption seems strong but is valid for many microbiome studies including multi-arm clinical experiments. For the real data used in this paper, the mice were under the same condition at the beginning of the experiment, where all mice were fed using the microbial community from a freshly voided fecal sample from a healthy adult human. Later on, half of the mice were maintained on a low-fat diet and half of the mice were switched to Western diet. The second assumption is that the majority of features are not differentially abundant between two adjacent time points under the same condition. Thus, the samples can be normalized from comparison of the first time point to the second point, and then the third, till the last one. Using this approach, samples at a later time point are normalized using the information from the previous time point.

### 2.2 Intra-time normalization

Experiencing the same situation, like at a given time point and under a certain condition, biological samples are assumed similar or homogeneous. Thus, the Intra-time normalization is conducted for count data from the microbial samples collected at the same time point and under the same condition (e.g., healthy or diseased). 16S rRNA sequencing is widely used in longitudinal microbiome studies, with applications in health and disease, environmental studies, and dietary studies. It enables the study of microbial diversity and dynamics over time. However, it suffers from under-sampling (Kaul et al., 2017; Kodikara et al., 2022). Due to limited sequencing depths, some features, especially relatively low abundant features, only appear in a small number of samples. These rare features should be excluded from normalization. At a given time point, dominant features (i.e., the features with abundant reads) need to be comparable across the samples under the same condition. Hence, these common dominant features can be used for normalizing the microbial samples under the same condition.

Assume that *n* features are contained across *m* samples. Some features are dominant in abundance, while quite a few are rare. For a given condition, let $c_{ijk}$ represent the number of reads in sample j assigned to feature i at time *k*. Intra-time normalization involves the following steps:

(a) Consider the sub dataset containing the dominant features only (assume $n_{0}$ features out of n features are dominant), and estimate the proportion of each feature using the ratio of the sum of counts for the feature to the grand total of counts:

$${\hat{p}}_{ik}=\sum_{j=1}^{m}c_{ijk}/\sum_{i=1}^{n_{0}}\sum_{j=1}^{m}c_{ijk}$$

(b) Estimate the sample scale for sample j by the average:

$$s_{jk}=\sum_{i=1}^{n_{0}}\left(\frac{c_{ijk}}{{\hat{p}}_{ik}}\right)/n_{0}$$

(c) Calculate the scale factor of sample j as ${DSS}_{jk}=\frac{s_{jk}}{median\left(s_{1k},\ldots ,{\;s}_{mk}\;\right)}$ . A normalized count is thus given by $c_{ijk}^{*}=\frac{c_{ijk}}{{DSS}_{jk}},i=1,\ldots ,n;j=1,\ldots ,m;\;k=1,\ldots ,K$ .

### 2.3 Bridge normalization

Bridge normalization contains two stages: i) normalization of samples at the initial time point, and ii) normalization of samples at two adjacent time points within the same condition. For the first stage, a group of stable features between two conditions at the initial time point is used for normalization. For the second stage, subsequently within each condition, a group of stable features across two adjacent time points is used for normalization. To identify the stable features, the RAIDA method (Sohn et al., 2015) is applied. RAIDA is developed for identifying differentially abundant features between two conditions/groups. Note, the term “conditions” in the RAIDA method is more general. In our current setting it could be either the two (treatment) conditions for the comparison at the initial time point or two adjacent time points within the same condition.

The RAIDA method computes the ratio between features, identifies the stable features between two conditions as a common divisor, and detects the differentially abundant features. We consider a zero-inflated log-normal distribution for the ratios:

$$r_{ijk}=\left\{\begin{array}{l} 0,\text{with probablity }\rho_{ik}\\ \log-\text{normal }\left(\mu_{ik},\sigma_{ik}^{2}\;\right),\text{with probablity }1-\rho_{ik} \end{array}\right.$$

where $r_{ijk}$ represents the ratio of $c_{ijk}^{*}$ to $c_{djk}^{*}$ , and *d* represents a feature or a set of features used as a divisor, i.e., scaling factor and $c_{ijk}^{*}$ denotes the Intra-time normalized count for feature i and sample j at time *k*. Details of normalization for the sample at two adjacent time points k,k+1 are given below (note: the normalization of samples for the two conditions at the initial time point can be treated as a special case of time series, e.g., treating the control condition as the previous time point and treatment condition as the later time point respectively, as the assumptions are essentially equivalent):

(a) Randomly choose a feature with non-zero counts in all samples and calculate the ratios using this feature as a divisor.
(b) At the time point *k*, for each of the features ( i = 1, … , *n*) estimate the parameters $\left(\mu_{ik},\sigma_{ik}^{2},\rho_{ik}\right)$ using the Expectation-Maximization algorithm (Hastie et al., 2009).
(c) Within a time point, use mean $\mu_{ik}$ and variance $\sigma_{ik}^{2}$ to measure the similarity in abundance between features using the Bhattacharyya distance (Coleman and Andrews, 1979), and then apply hierarchical clustering based on the Bhattacharyya distance. Create a set of clustered features common in both time *k* and *k* +1 and use it as possible common divisors.
(d) For each of the new clusters obtained after step (c), sum up the counts for the involved features and get a “combined” feature which is used as a new divisor to calculate new ratios. Then estimate the parameter using step (b) for the new ratios for time k and k+1. Construct a likelihood ratio (LR) test for the log ratio of each feature and obtain the p-value (Chen J. et al., 2018). We fit the model using MLE under both the null and alternative models. The LR statistic can be easily obtained by fitting the zero-inflated log-normal (ZILN) model to all samples and the samples from each group separately. The new cluster giving the minimum number of differentially abundant features (whose p-value is less than a certain significance level) is a set of stable features, i.e., Ω , and the scale factor between time k and k+1 is calculated as:

$$\Delta_{k,k+1}=\frac{\sum_{i\in \Omega }\sum_{j\;}c_{ij\left(k+1\right)}^{*}}{\sum_{i\in \Omega }\sum_{j\;}c_{ijk}^{\prime }}$$

where ${c_{ijk}}^{\prime }$ represents the Bridge normalized counts for the samples at time *k*, calculated as: ${c_{ijk}}^{\prime }=c_{ijk}^{*}/\Delta_{k-1,k}$ . In such way, samples are normalized sequentially. The algorithm of Bridge normalization can be found in the Supplementary Figure S1.

## 3 Results

### 3.1 Simulation settings to evaluate the performance of TimeNorm

A series of simulation studies were conducted to evaluate the performance of the proposed normalization method and compare it with existing methods, namely TMM, CSS, TC and GMPR. The data were simulated under zero-inflated Negative Binomial (ZINB) distributions using the copula package in R. A simulated dataset consists of two conditions (conditions one and two or treatment and control), 500 features over six time points, and ten samples (individuals) from each condition; 100 out of 500 features were assumed to be differentially abundant features (DAFs) between the two conditions. We considered two levels of zero proportions: 0.3 (low) and 0.7 (high), and two levels of dispersion: 500/ μ (low) and 150/ μ (high), where μ is the average count for the features in the control samples simulated from the ZINB model. The autoregressive AR1/exchangeable correlation ρ=0.6 was used to simulate the within-subject correlation due to repeated measures over the timepoints. We named these four combinations of zero proportion and dispersion levels Test 1 ∼ 4 (Table 1). In addition, four scenarios for each simulated dataset were considered: (A) Differentially abundant (DAF) features exhibit an increasing pattern over time for the treatment group with no dynamic changes observed in the control group. Meanwhile, non-DAF features demonstrate a consistent absence of change over time, with no discernible differences between the two groups; (B) Based on scenario A, the total count across all features in each sample in the treatment group (condition 1) at the last time point is double that of its original count as setting A, including DAFs; (C) Based on scenario A, at each time point two randomly selected samples (out of 10 samples) are half of their original count value; (D) Based on scenario A, for any sample under the treatment condition, the total count across all features at each time point exhibits similar magnitudes. In total, we examined the performance of the methods on datasets simulated with 16 settings. Out of the four tests, both Test 2 and 4 accurately mimics the characteristics of real microbiome data, being zero-inflated and dispersed. Among four distinct settings, setting D, in which the total count across samples under the treatment condition have similar size, closely replicates the DAFs observed in real microbiome data.

**TABLE 1:** Summary of parameter settings for the simulation studies, where μ denotes the means of features. Four scenarios A∼D are examined for each of these four combinations of the zero proportion and the dispersion level.

		Zero proportion	
		0.3	0.7
Dispersion	500/ μ	Test 1	Test 2
	150/ μ	Test 3	Test 4

The five scaling normalization methods (TMM, CSS, GMPR, TC, and TimeNorm) were compared for each scenario under each setting, with ten replications per combination. The performance was measured by Root of Relative Mean Square Error (RRMSE):

$$RRMSE=\sqrt{\frac{1}{2mnK}\sum_{l=1}^{2}\sum_{k=1}^{K}\sum_{j=1}^{m}\sum_{i=1}^{n}{\left(\frac{{\hat{\mu }}_{ijlk}-\mu_{ijlk}}{\mu_{ijlk}}\right)}^{2}}$$

where $\mu_{ijlk}$ is the true abundance and ${\hat{\mu }}_{ijlk}$ is the estimated abundance for feature *i* in sample *j* under condition l at time *k*; n is the number of features, m is the number of samples, and *K* is the number of time points. We also evaluated the performance by carrying out differential abundance analysis using metaDprof (Luo et al., 2017) and SplinectomeR (Shields-Cutler et al., 2018). metaDprof is a spline-based method to detect differentially abundant features for time series metagenomic data. This method comes with its default normalization method TMM and uses the Benjamin-Hochberg procedure to control the false discovery rate (FDR). SplinectomeR uses smoothing splines for hypothesis testing in longitudinal studies. To study the performance of various normalization methods and differential analyses, type I error, power and receiver operating characteristic (ROC) curves for each method are also compared.

### 3.2 Results of simulation studies

**FIGURE 2:**
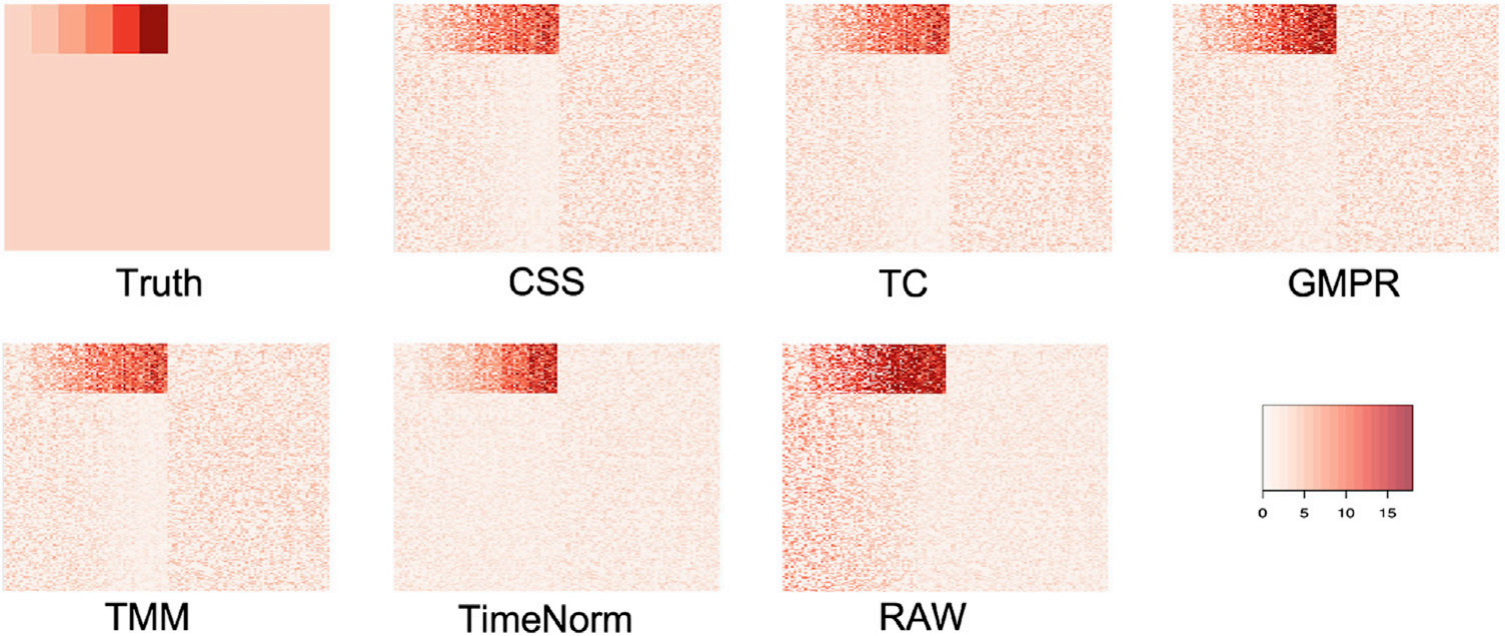
Heatmap of simulated data from ground truth, raw count (with noise), and normalized data using CSS, TS, TMM, TimeNorm, and GMPR for the setting Test 2D. Each row represents a feature and the 100 non-differentially abundant features are at the top. In each plot the left half is the time series profiles for features under condition one (treatment group) and right half for features under condition two (control group).

**FIGURE 3:**
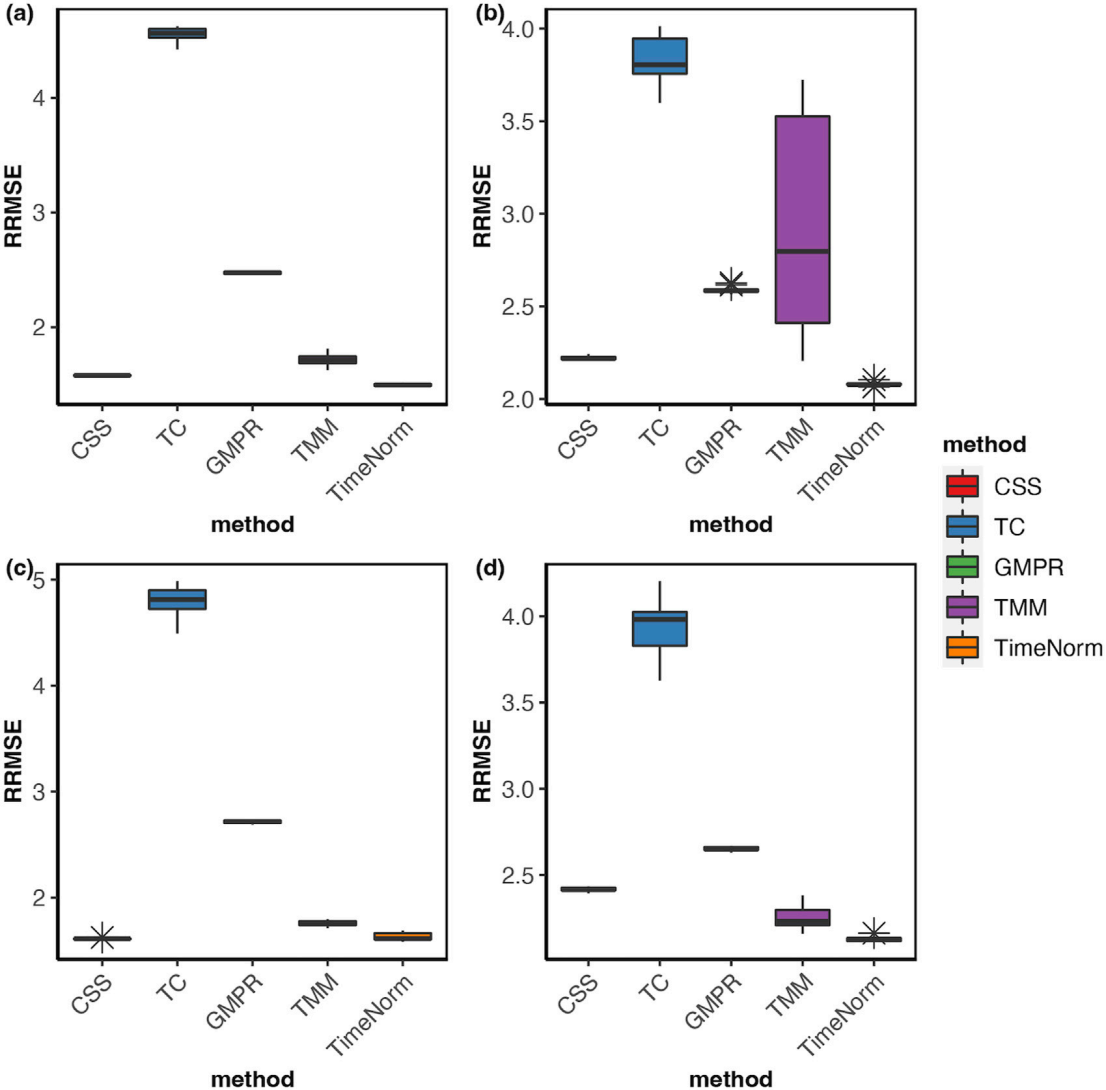
Boxplots of Relative Root Mean Square Error for comparison of different normalization methods based on ten replicated simulations for **(A)** Test 1D, **(B)** Test 2D, **(C)** Test 3D and **(D)** Test 4D. The short error bars represent the standard deviation from ten replications.

As we considered 16 simulation settings, only results for Test 2 scenario D (Test 2D) which mimic the real microbiome studies in each test, are shown here (scenario A-C results are given in the Supplementary Figures S2–S16). The expression profiles for the ground truth, raw counts, and five normalized data were shown and compared using heatmaps (Figure 2). It can be seen that results from TimeNorm exhibit a closer pattern to the ground truth. In the simulation studies, the abundance of non-DAFs in condition one is the same as the features in condition two across all time points. There is an identifiable vertical pattern on each of the heatmaps by CSS, TC, GMPR, and TMM, indicating a separation between the two conditions for the non-DAF. For the raw count data, the color for condition one is generally darker than condition two. Only TimeNorm indicates no difference between the two conditions for the non-DAFs which is demonstrated through the color scale in heatmaps. Figure 3 shows the results of RRMSE for various settings. RRMSE is a relative error measure, where errors are scaled based on true settings. Lower RRMSE values are expected for normalization methods that are closer to the ground truth. TimeNorm outperforms other normalization methods with lower RRMSE. Rarefying is a conventional method for microbiome normalization. In our simulation study for Test 2, settings A–D (Supplementary Figure S20), we also applied rarefying normalization. This approach often results in higher RRMSE values compared to TimeNorm. McMurdie and Holmes (2014) found that rarefied counts could lead to an unacceptable high rate of false positive OTUs and fail to address over-dispersion, thus inducing a systematic bias that increases the Type-I error rate, even after corrections for multiple hypotheses are made (McMurdie and Holmes, 2014). Consequently, we did not use rarefying in the differential abundance (DA) analysis.

The comparative performance metrics from the DA analysis using metaDprof on the normalized data for Test 2D are shown in Figures 3, 4. All normalization methods controlled the Type I error under 0.05 for different settings. TimeNorm has the highest power to detect DAFs. Figure 5 displays the results of mean ROC for Test 1D∼4D. TimeNorm outperforms other methods when the zero proportion is high. For settings A-C across tests 1 – 4 (Supplementary Figures S17–S19, S21–S35, S52, S53), similar conclusions to those in Test 2D can be drawn. Overall, TimeNorm surpasses other existing normalization methods for time-series data. The results from splinectomeR DA method (Supplementary Figures S36–S51) are consistent with those from metaDprof. splinectomeR has the same power to detect DAFs in different normalization methods. However, TimeNorm has higher precision, specificity, F1 score, and lower Type-I error.

**FIGURE 4:**
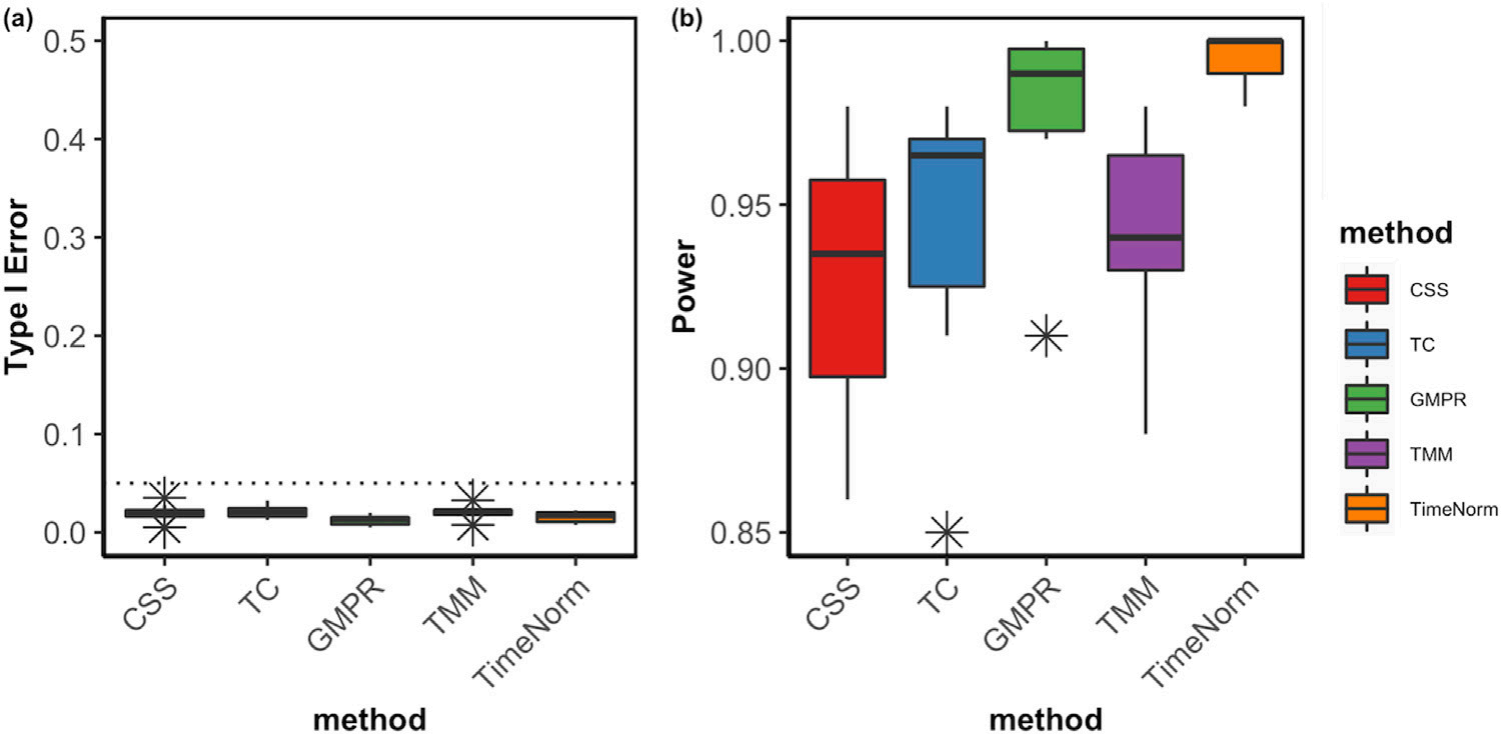
Boxplots of Type I error **(A)** and power of DA analysis **(B)** using metaDprof on the normalized data using various methods based on ten replicated simulations for Test 2D. The short error bars represent the standard deviation from ten replications.

**FIGURE 5:**
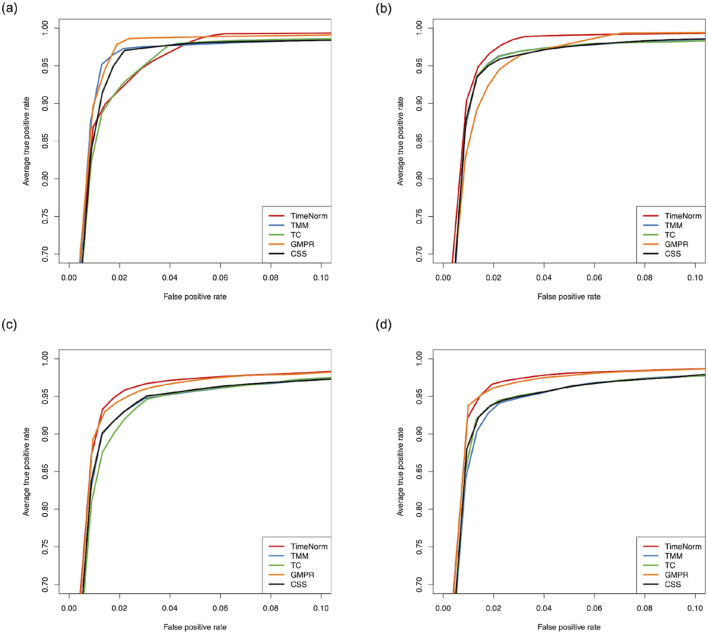
Partial area of mean Receiver Operating Characteristic (ROC) for various settings based on ten replicated simulations with 500 features. **(A)** Test 1D; **(B)** Test 2D; **(C)** Test 3D; and **(D)** Test 4D.

The proposed normalization method relies on the choice of stable features detected by RAIDA. A sensitivity analysis was conducted in a simulation study to test the robustness by choosing a set with second least number of different features as stable features. The comparison result is shown in Supplementary Figure S54 where TimeNorm1 represents the result using the minimum number of different features as stable features while TimeNorm2 uses the second least number of different features. It is noticed that two options lead to very close results, which implies that the selection of the set is robust while the one with the least number of different features results in the optimal value.

### 3.3 Real data analysis

**FIGURE 6:**
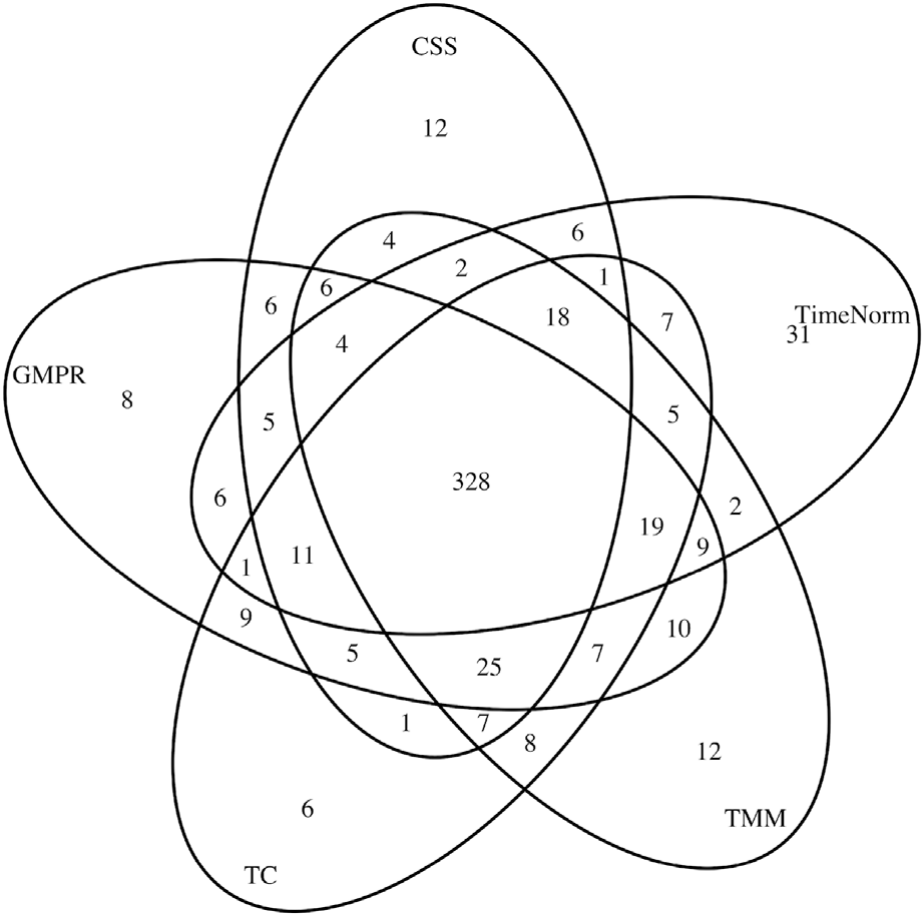
Venn diagram of detected differentially abundant feature lists for different normalization methods: CSS, TMM, TC, GMPR, and TimeNorm.

In addition to simulation studies, we applied TimeNorm to real data. We used a publicly available dataset from a longitudinal study (Turnbaugh et al., 2009). Fecal samples were collected from 12 adult mice (6 underwent a plant polysaccharide-rich diet (Low Fat) and 6 underwent a high-fat and high-sugar diet (Western diet) during 8 weeks. To characterize the microbial taxa present within the samples, polymerase chain reaction (PCR) from variable region 2 of the 16s rRNA gene was used to perform 16s rRNA sequencing. The microbiome count data were obtained through metagenomeSeq R package (Paulson et al., 2013). Low-Fat mice were compared with Western diet mice, over 910 operational taxonomical units (OTUs). First, we applied different normalization methods (CSS, TMM, TC, GMPR, and TimeNorm) to these data and then carried out differential abundance analysis using metaDprof. OTUs were detected as statistically significant at 0.05 level, post Benjamin-Hochberg adjustment. We plotted the Venn diagram to summarize the results using different normalization methods (Figure 6).

**FIGURE 7:**
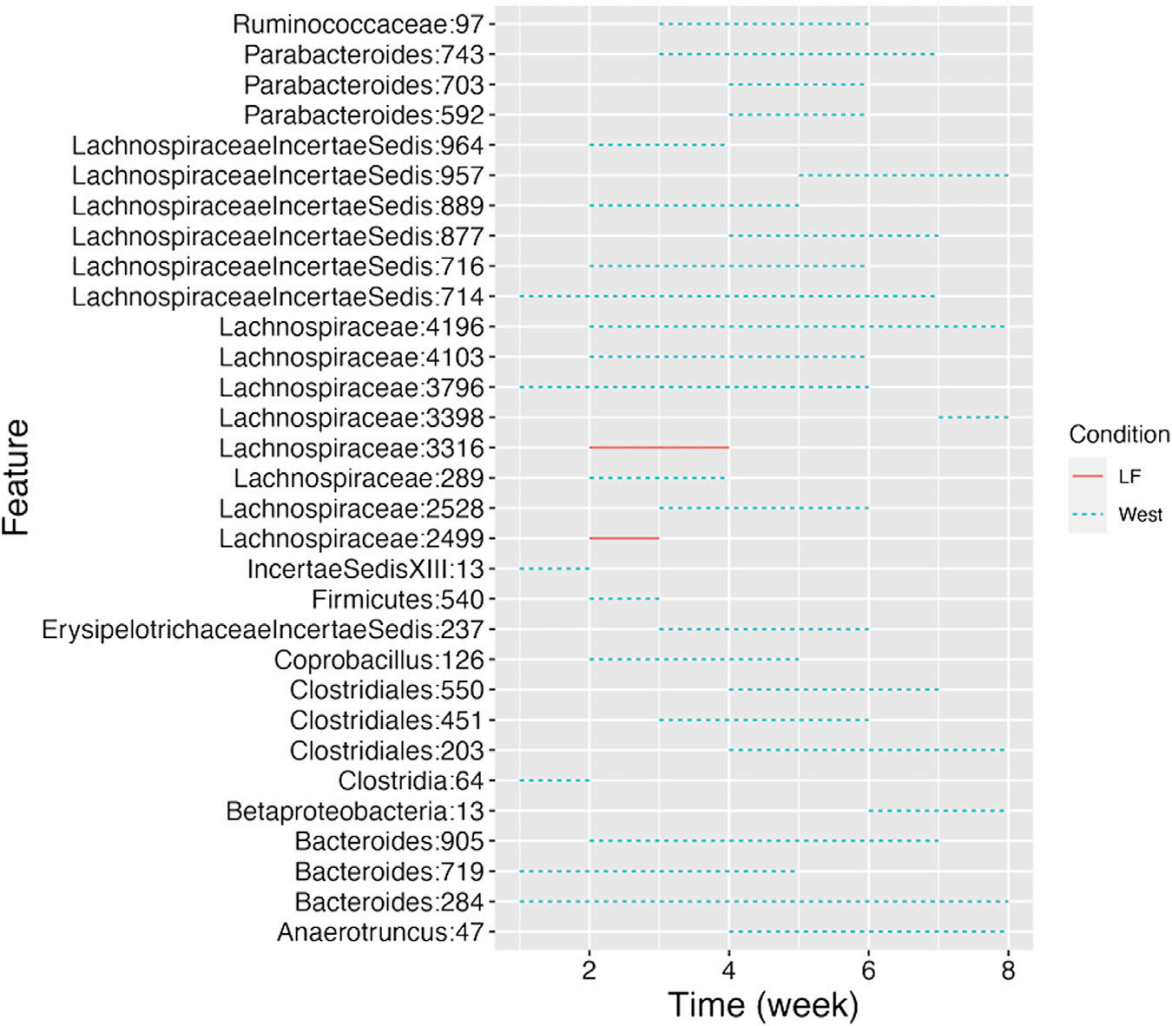
Detected time intervals for the 31 unique OTUs. The segment for each OTU indicates the start and end week during which the abundance difference arises between the two types of diet. The solid segment represents that the abundance in the low-fat group is higher than that in the Western diet group, while the dotted segment indicates a higher abundance in Western diet group.

CSS, TMM, TC, GMPR and TimeNorm identified 456, 486, 475, 485, and 475 significant OTUs respectively. Among the significant OTUs detected by different normalization and DA methods, 328 OTUs were commonly identified by all normalization methods. 31 unique OTUs were detected in the data normalized by TimeNorm (Table 2) and the detected time intervals are plotted in Figure 7. Among the unique OTUs, 2 OTUs were more abundant in the low-fat group from week 2 to week 4. The majority of these belong to the class of Clostridia (phylum Firmicutes), Bacteroidia (phylum Bacteroidota) and Erysipelotrichia (phyla Firmicutes). One of these OTUs belonging to Betaproteobacteria (phylum Proteobacteria) was significantly more abundant in the Western diet group. A recent study reported similar findings; Betaprotebobacteria was significantly enriched only in the High-Fat sugar group (Shon et al., 2019). The proposed method could discover relevant features for this study that might be missed using other normalization methods.

**TABLE 2:** 31 OTUs uniquely identified by TimeNorm using metaDprof ( + indicates the OTU is abundant in Western diet or Low-Fat group).

Class	OTU	Western diet	Low fat	pvalue	padj
Clostridia	LachnospiraceaeIncertaeSedis:877	+		0.000	0.000
Clostridia	LachnospiraceaeIncertaeSedis:964	+		0.000	0.000
*Bacteroidetes*	*Bacteroides*:905	+		0.002	0.015
Erysipelotrichi	Coprobacillus:126	+		0.002	0.015
*Bacteroidetes*	Parabacteroides:592	+		0.002	0.015
*Bacteroidetes*	Parabacteroides:703	+		0.002	0.015
*Bacteroidetes*	*Bacteroides*:719	+		0.004	0.020
*Bacteroidetes*	Parabacteroides:743	+		0.004	0.020
Clostridia	Lachnospiraceae:2499		+	0.006	0.025
Betaproteobacteria	Betaproteobacteria:13	+		0.014	0.031
Clostridia	Clostridia:64	+		0.018	0.031
Erysipelotrichi	ErysipelotrichaceaeIncertaeSedis:237	+		0.020	0.031
NA	Firmicutes:540	+		0.014	0.031
Clostridia	IncertaeSedisXIII:13	+		0.018	0.031
Clostridia	Lachnospiraceae:2528	+		0.018	0.031
Clostridia	Lachnospiraceae:4103	+		0.014	0.031
Clostridia	Lachnospiraceae:4196	+		0.020	0.031
Clostridia	LachnospiraceaeIncertaeSedis:957	+		0.016	0.031
Clostridia	LachnospiraceaeIncertaeSedis:716	+		0.028	0.035
Clostridia	Clostridiales:451	+		0.030	0.036
*Bacteroidetes*	*Bacteroides*:284	+		0.032	0.037
Clostridia	Lachnospiraceae:3398	+		0.034	0.038
Clostridia	Ruminococcaceae:97	+		0.036	0.039
Clostridia	LachnospiraceaeIncertaeSedis:714	+		0.038	0.041
Clostridia	Clostridiales:203	+		0.040	0.042
Clostridia	Lachnospiraceae:3316		+	0.040	0.042
Clostridia	LachnospiraceaeIncertaeSedis:889	+		0.044	0.046
Clostridia	Anaerotruncus:47	+		0.046	0.046
Clostridia	Lachnospiraceae:289	+		0.046	0.046
Clostridia	Clostridiales:550	+		0.048	0.048
Clostridia	Lachnospiraceae:3796	+		0.048	0.048

## 4 Discussion

Normalizing sequencing-based microbiome data is a commonly used but critical preprocessing step before performing downstream analysis, which serves to eliminate sequence depth bias and make samples comparable. The characteristic zero-inflation and over-dispersion in microbiome sequencing data makes it vital to choose an appropriate normalization method that effectively lowers false-positive rates in the DA analysis.

This study proposes the first normalization method tailored for time-series microbiome count data, which considers the latter’s compositional property and time dependency by performing both the within and across time point normalizations. The simulation studies show that the proposed method surpasses existing normalization methods by exhibiting a closer pattern to the ground truth manifested with lower error rate. We also demonstrate that TimeNorm outperforms other normalization methods in DA analysis. We apply different normalization techniques on the simulated datasets and perform DA analysis using two different approaches; TimeNorm yields good Type I error control and performance in detecting DA features, especially in mimicking the real microbiome study. Furthermore, in the study examining the effect of diet on microbial community composition, we demonstrate that DA analysis using metaDprof on TimeNorm normalized data is able to detect significantly differentially abundant features missed by other normalization methods. These findings are consistent with those reported in the previous literature, providing insight into studying microbial communities. Overall, TimeNorm outperforms TMM, TC, CSS and GMPR in various settings, particularly when the zero proportion is high.

In the illustration of Figure 1 and simulation studies, we assume the two conditions are comparable at the initial time point. It is not necessary. We can assume two conditions are quite different at the initial point, but comparable or similar at the last time point (e.g., some antibiotic medicine is applied to the disease samples and eventually the samples under two conditions become similar). In such way we need to start our normalization procedure from the last point and then go back to the first point step by step.

Since the TimeNorm method is distribution-based, estimating the parameter through the EM algorithm is computationally time-intensive. TimeNorm calculates the size factors for a typical microbiome dataset in minutes. When sample size is larger, it will take more time. Currently, TimeNorm calculates the size factors for Bridge normalization for every two adjacent time points. When there are more time points (Gusareva et al., 2020), normalizing the samples across three adjacent points might be more accurate, resulting in a set of more stable features. However, the challenge could be finding the stable features for multiple timepoints, as for longer time series, there are fewer stable features. Hence, our future research will focus on Bridge normalization for multiple time points.

In conclusion, we demonstrate that applying TimeNorm on both simulated and real datasets could normalize time-course microbiome data appropriately with lower RRMSE. Additionally, it empowers the DA analysis. We believe that TimeNorm can serve as a useful approach for time-course microbiome data normalization.

## Data Availability

The real data analyzed in this study is publicly available. It can be found in the metagenomeSeq R package (https://bioconductor.org/packages/release/bioc/html/metagenomeSeq.html). The R code for TimeNorm can be downloaded from github.com/anlingUA/TimeNorm.
