# Supplementary material for: TimeNorm: a novel normalization method for time course microbiome data
Source: Front Genet. 2024 Sep 24;15:1417533. doi: 10.3389/fgene.2024.1417533 (PMC11458461; doi:10.3389/fgene.2024.1417533)
Supplement: Supplementary file 1 [file DataSheet1.pdf]

TimeNorm: a novel normalization method for time course microbiome data

Qianwen Luo[1], Meng Lu[2], Hamza Butt[3], Nicholas Lytal[4], Ruofei Du[5], Hongmei Jiang[6], Lingling An[1,2,3]

[1]Department of Biosystems Engineering, University of Arizona, Tucson, AZ, USA
[2] Graduate Interdisciplinary Program in Statistics and Data Science, University of Arizona, Tucson, AZ, USA
[3]Department of Biostatistics and Epidemiology, University of Arizona, Tucson, AZ, USA
[4]Department of Mathematics and Statistics, California State University at Chico, Chico, CA, USA
[5]Department of Biostatistics, University of Arkansas for Medical Sciences, Little Rock, AR, USA
[6]Department of Statistics and Data Science, Northwestern University, Evanston, IL, USA

*** Correspondence:**
Lingling An
anling@arizona.edu

Supplemental Materials

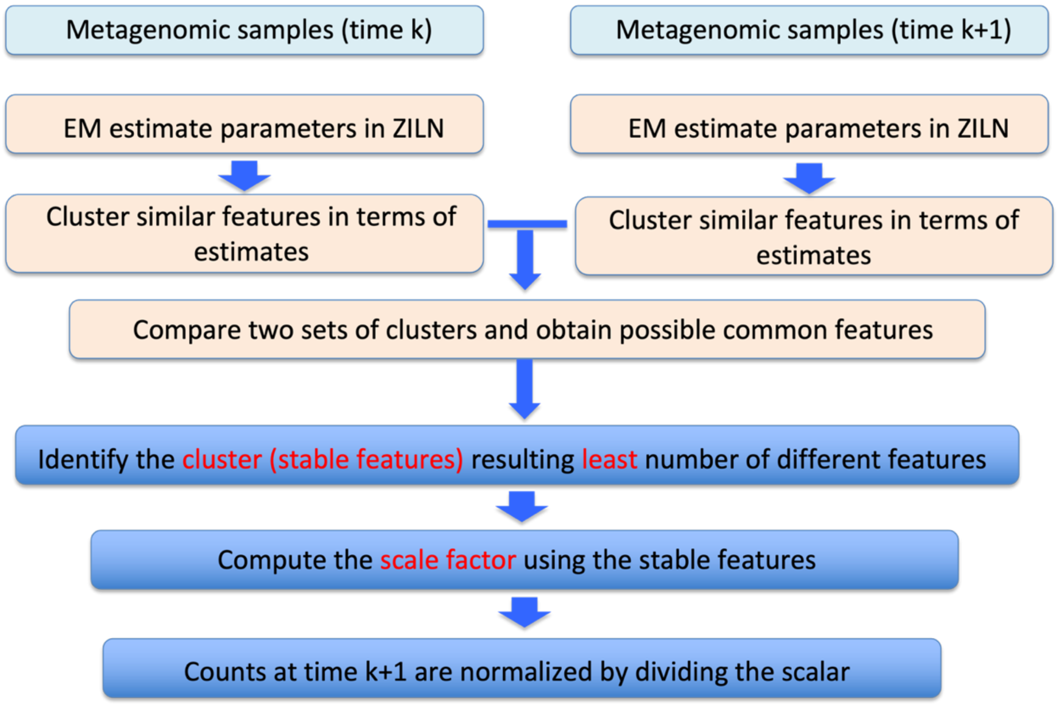

S1: Bridge normalization flowchart.

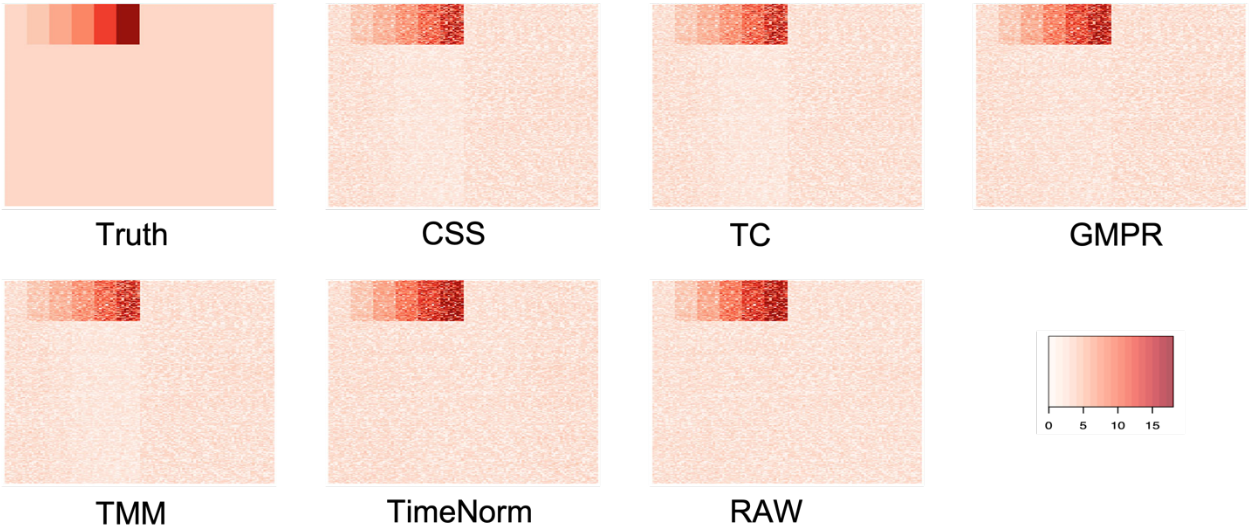

S2: Heatmap of simulated data from ground truth, raw count (with noise), and normalized data using CSS, TS, TMM, TimeNorm, and GMPR for the setting **Test 1A**. Each row represents a feature and the 100 DAFs are at the top. In each plot the left half is the time series profiles for features under condition one (treatment group) and right half for features under condition two (control group).

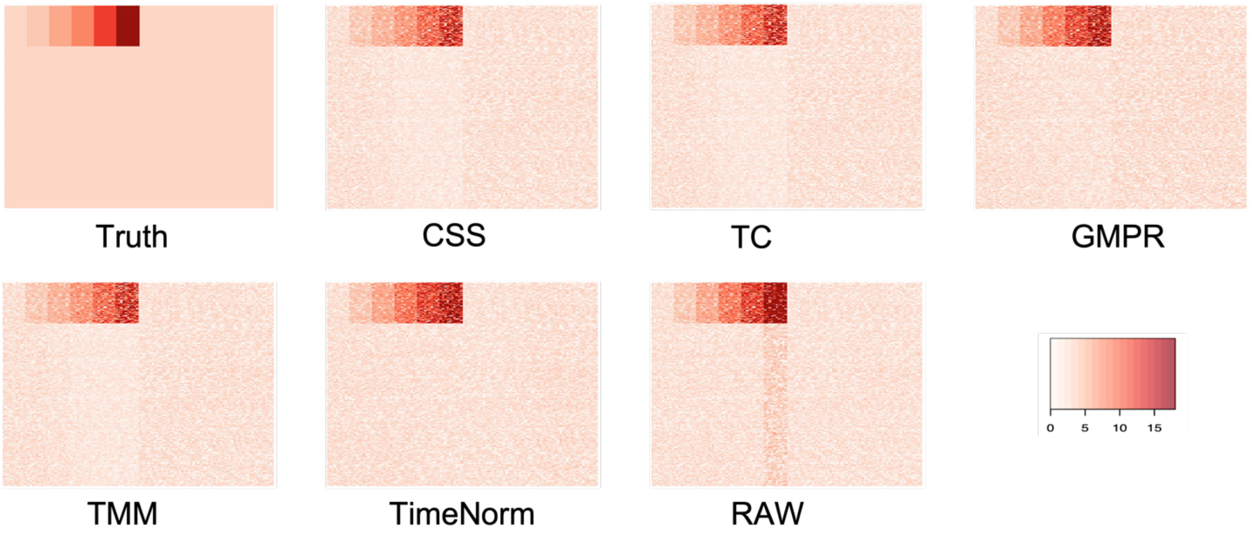

S3: Heatmap of simulated data from ground truth, raw count (with noise), and normalized data using CSS, TS, TMM, TimeNorm, and GMPR for the setting **Test 1B**. Each row represents a feature and the 100 DAFs are at the top. In each plot the left half is the time series profiles for features under condition one (treatment group) and right half for features under condition two (control group).

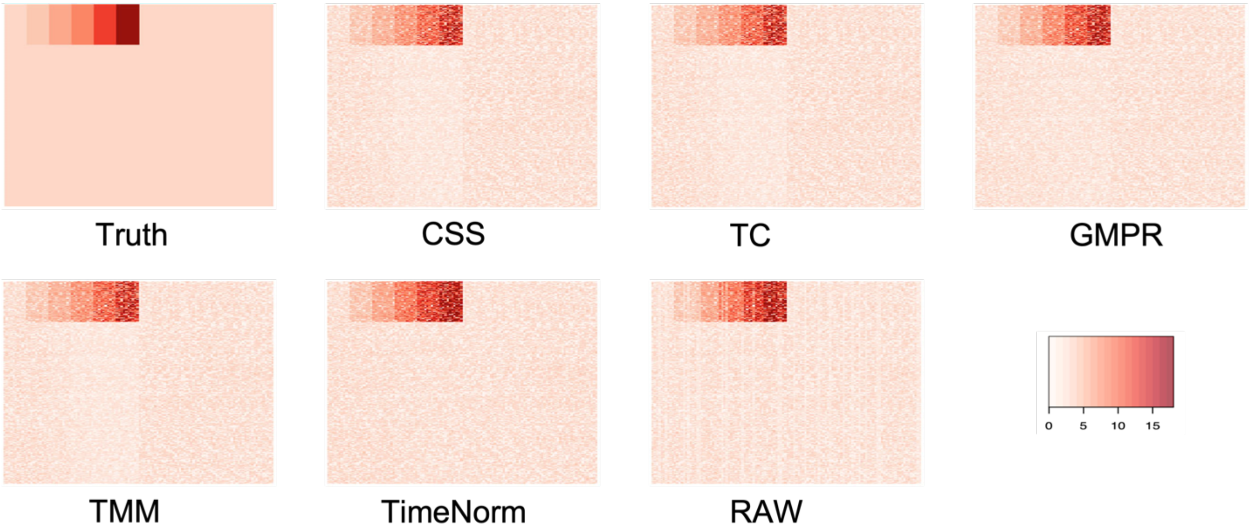

S4: Heatmap of simulated data from ground truth, raw count (with noise), and normalized data using CSS, TS, TMM, TimeNorm, and GMPR for the setting **Test 1C**. Each row represents a feature and the 100 DAFs are at the top. In each plot the left half is the time series profiles for features under condition one (treatment group) and right half for features under condition two (control group).

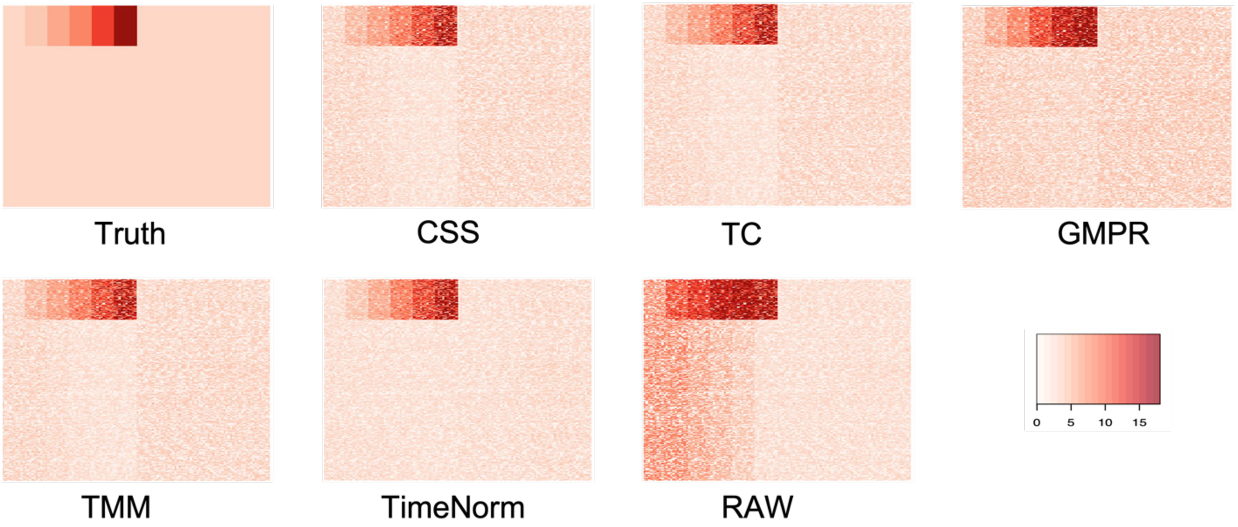

S5: Heatmap of simulated data from ground truth, raw count (with noise), and normalized data using CSS, TS, TMM, TimeNorm, and GMPR for the setting **Test 1D**. Each row represents a feature and the 100 DAFs are at the top. In each plot the left half is the time series profiles for features under condition one (treatment group) and right half for features under condition two (control group).

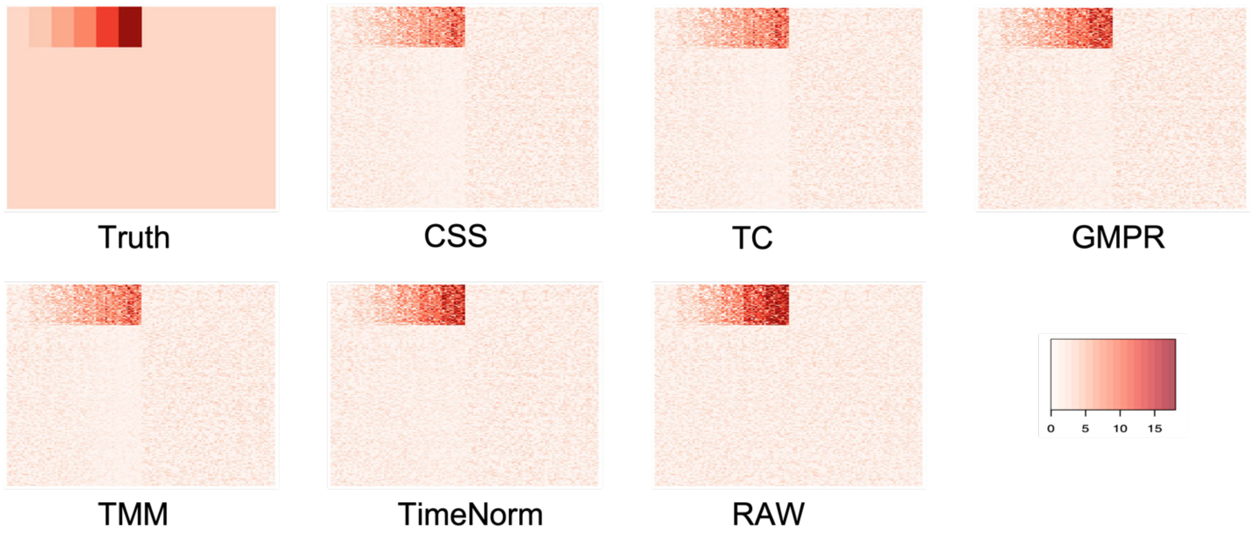

S6: Heatmap of simulated data from ground truth, raw count (with noise), and normalized data using CSS, TS, TMM, TimeNorm, and GMPR for the setting **Test 2A**. Each row represents a feature and the 100 DAFs are at the top. In each plot the left half is the time series profiles for features under condition one (treatment group) and right half for features under condition two (control group).

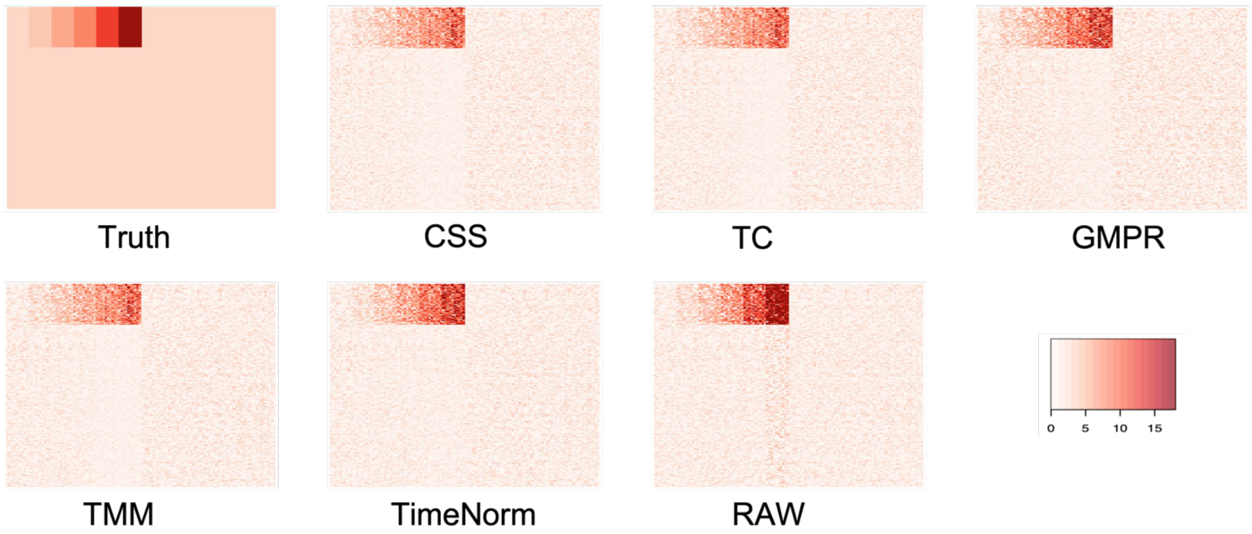

S7: Heatmap of simulated data from ground truth, raw count (with noise), and normalized data using CSS, TS, TMM, TimeNorm, and GMPR for the setting **Test 2B**. Each row represents a feature and the 100 DAFs are at the top. In each plot the left half is the time series profiles for features under condition one (treatment group) and right half for features under condition two (control group).

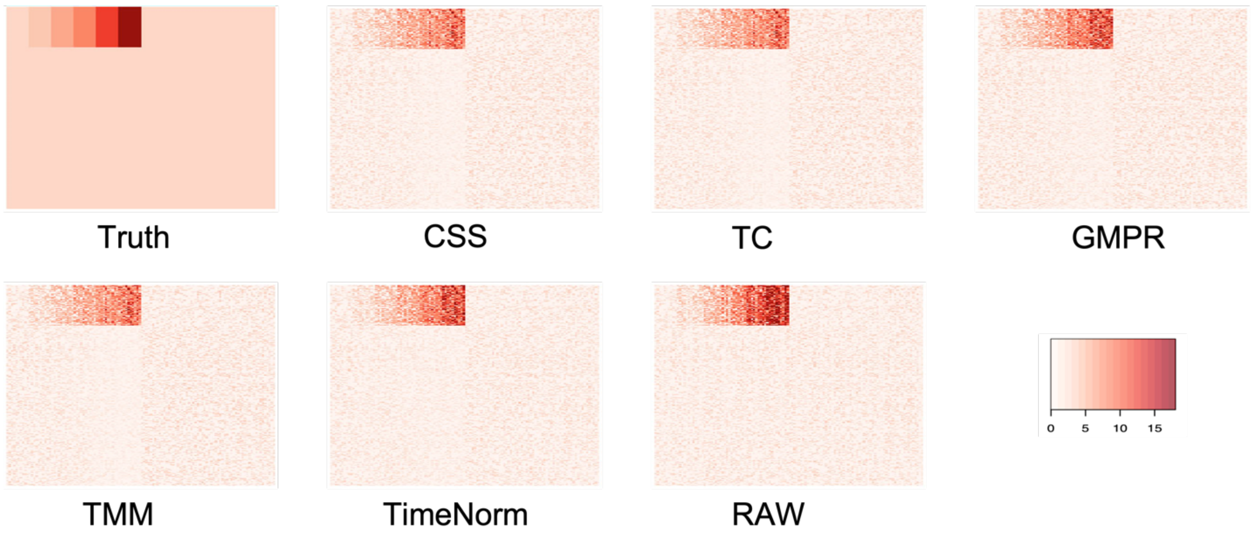

S8: Heatmap of simulated data from ground truth, raw count (with noise), and normalized data using CSS, TS, TMM, TimeNorm, and GMPR for the setting **Test 2C**. Each row represents a feature and the 100 DAFs are at the top. In each plot the left half is the time series profiles for features under condition one (treatment group) and right half for features under condition two (control group).

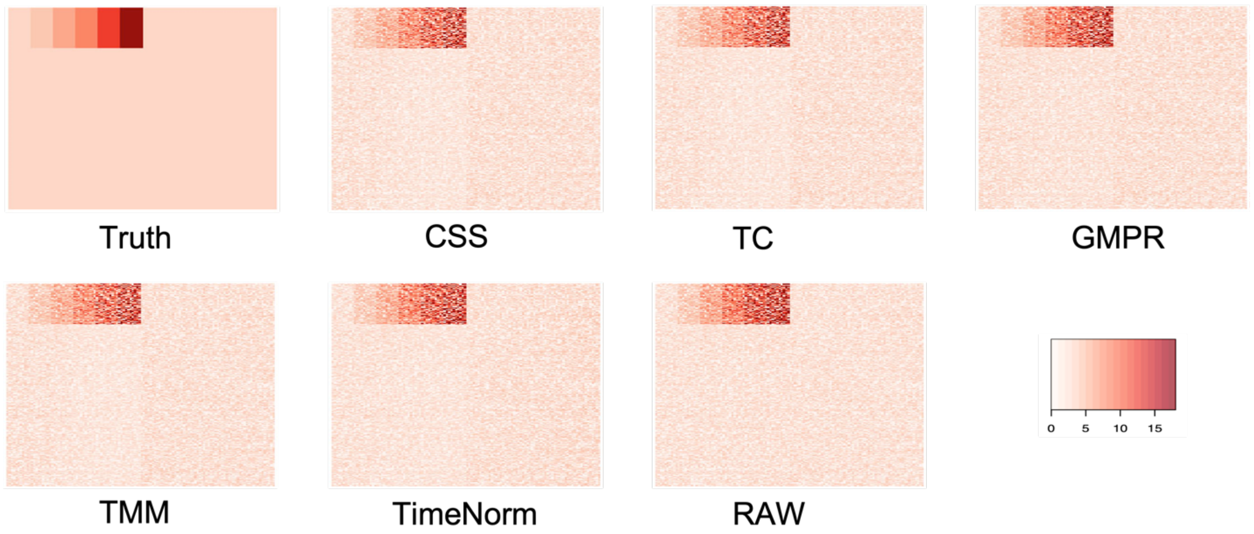

S9: Heatmap of simulated data from ground truth, raw count (with noise), and normalized data using CSS, TS, TMM, TimeNorm, and GMPR for the setting **Test 3A**. Each row represents a feature and the 100 DAFs are at the top. In each plot the left half is the time series profiles for features under condition one (treatment group) and right half for features under condition two (control group).

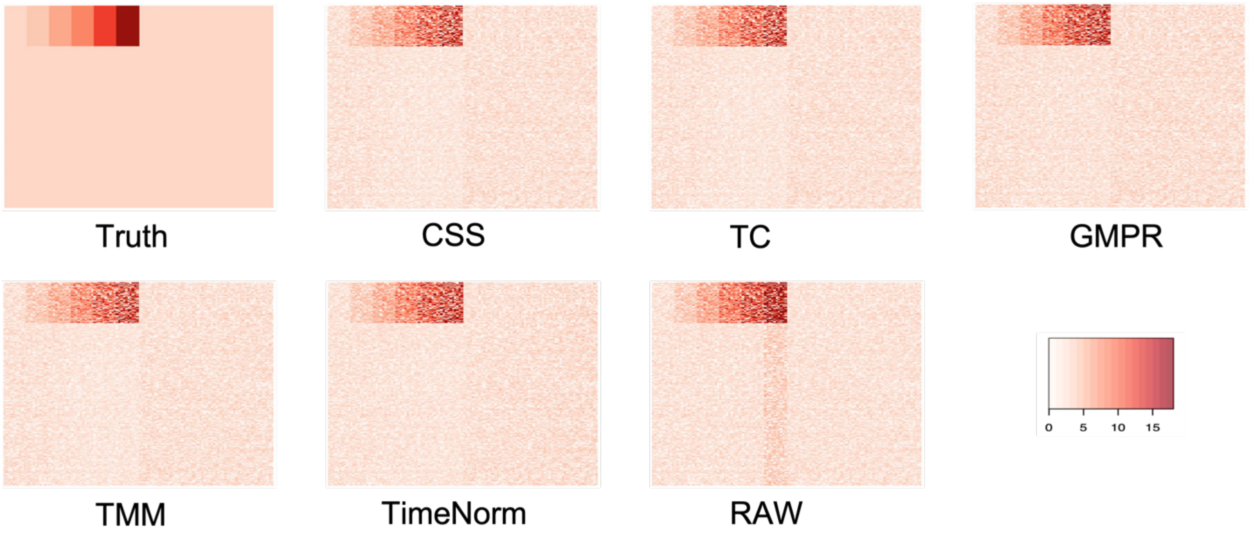

S10: Heatmap of simulated data from ground truth, raw count (with noise), and normalized data using CSS, TS, TMM, TimeNorm, and GMPR for the setting **Test 3B**. Each row represents a feature and the 100 DAFs are at the top. In each plot the left half is the time series profiles for features under condition one (treatment group) and right half for features under condition two (control group).

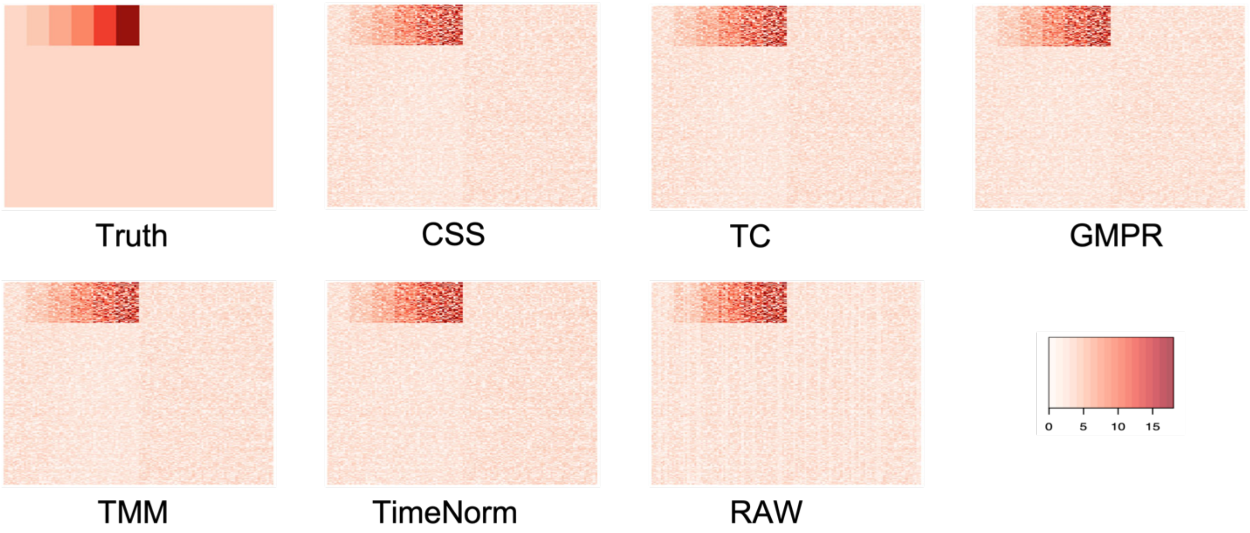

S11: Heatmap of simulated data from ground truth, raw count (with noise), and normalized data using CSS, TS, TMM, TimeNorm, and GMPR for the setting **Test 3C**. Each row represents a feature and the 100 DAFs are at the top. In each plot the left half is the time series profiles for features under condition one (treatment group) and right half for features under condition two (control group).

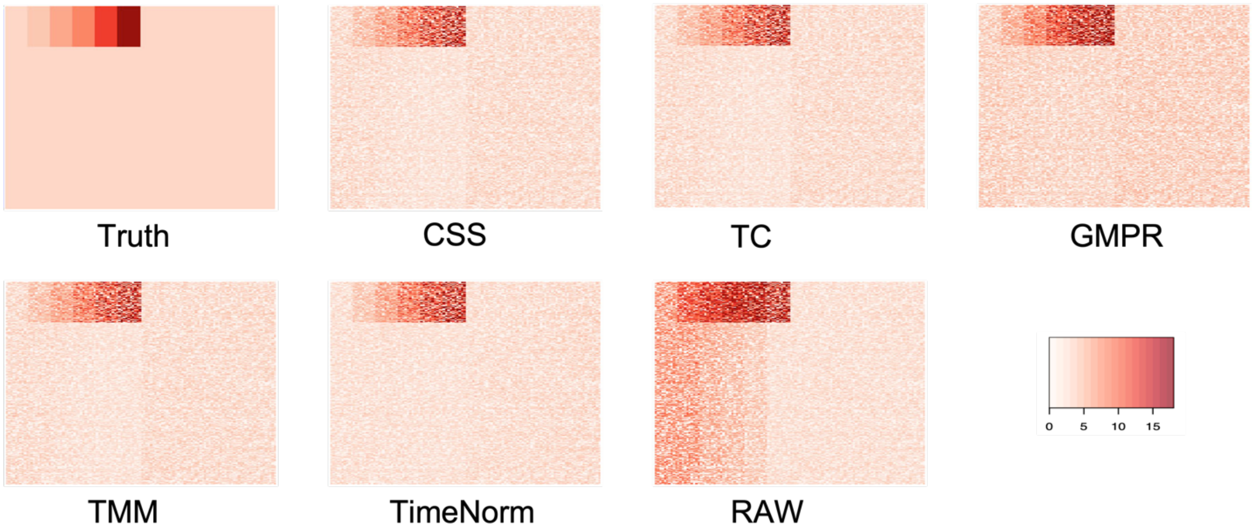

S12: Heatmap of simulated data from ground truth, raw count (with noise), and normalized data using CSS, TS, TMM, TimeNorm, and GMPR for the setting **Test 3D**. Each row represents a feature and the 100 DAFs are at the top. In each plot the left half is the time series profiles for features under condition one (treatment group) and right half for features under condition two (control group).

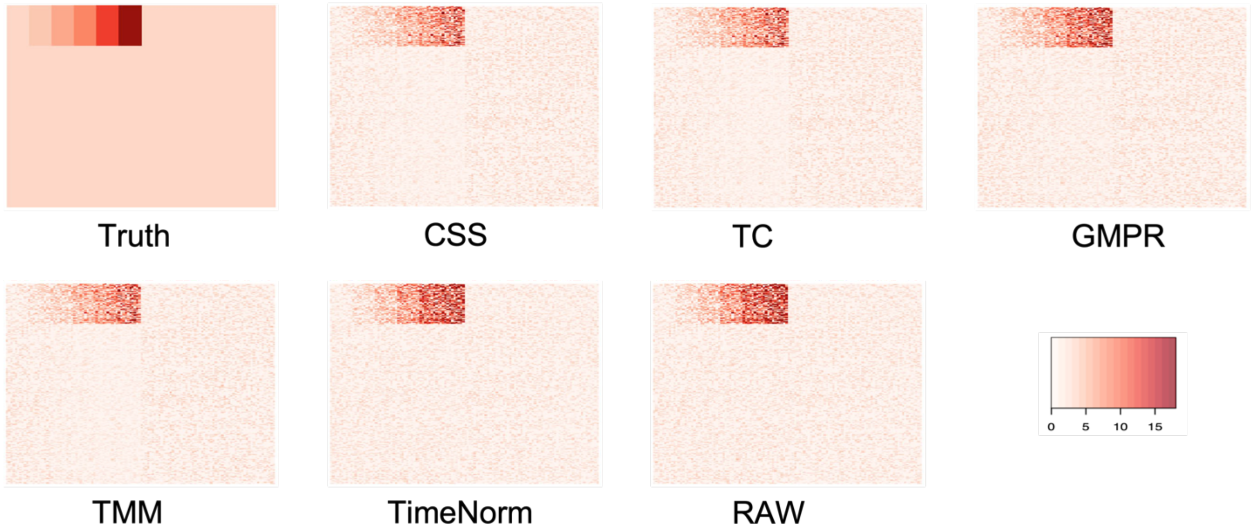

S13: Heatmap of simulated data from ground truth, raw count (with noise), and normalized data using CSS, TS, TMM, TimeNorm, and GMPR for the setting **Test 4A**. Each row represents a feature and the 100 DAFs are at the top. In each plot the left half is the time series profiles for features under condition one (treatment group) and right half for features under condition two (control group).

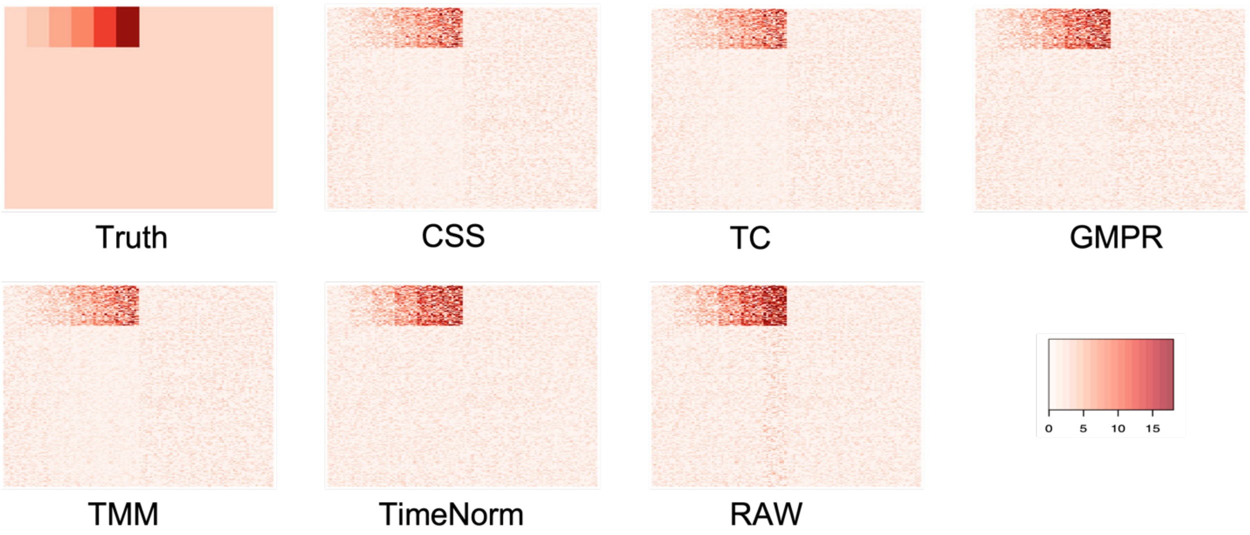

S14: Heatmap of simulated data from ground truth, raw count (with noise), and normalized data using CSS, TS, TMM, TimeNorm, and GMPR for the setting **Test 4B**. Each row represents a feature and the 100 DAFs are at the top. In each plot the left half is the time series profiles for features under condition one (treatment group) and right half for features under condition two (control group).

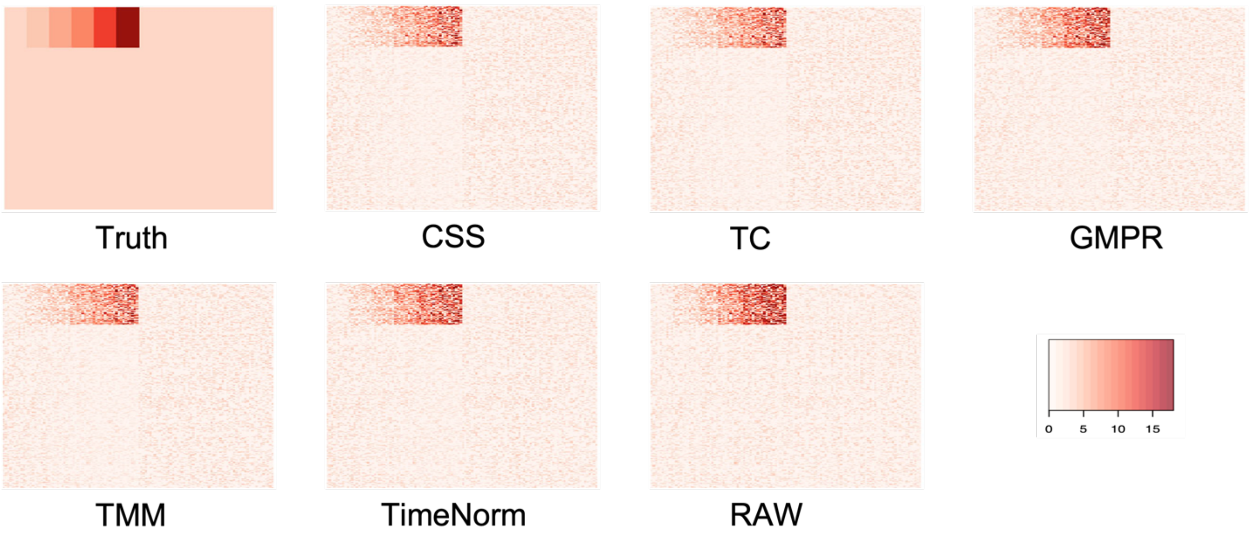

S15: Heatmap of simulated data from ground truth, raw count (with noise), and normalized data using CSS, TS, TMM, TimeNorm, and GMPR for the setting **Test 4C**. Each row represents a feature and the 100 DAFs are at the top. In each plot the left half is the time series profiles for features under condition one (treatment group) and right half for features under condition two (control group).

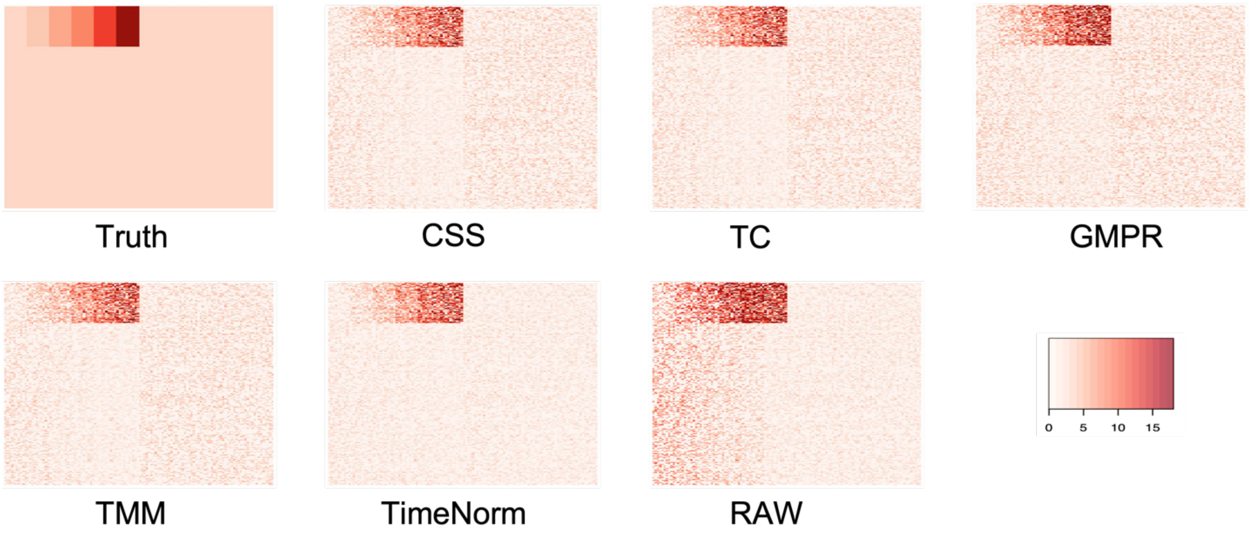

S16: Heatmap of simulated data from ground truth, raw count (with noise), and normalized data using CSS, TS, TMM, TimeNorm, and GMPR for the setting **Test 4D**. Each row represents a feature and the 100 DAFs are at the top. In each plot the left half is the time series profiles for features under condition one (treatment group) and right half for features under condition two (control group).

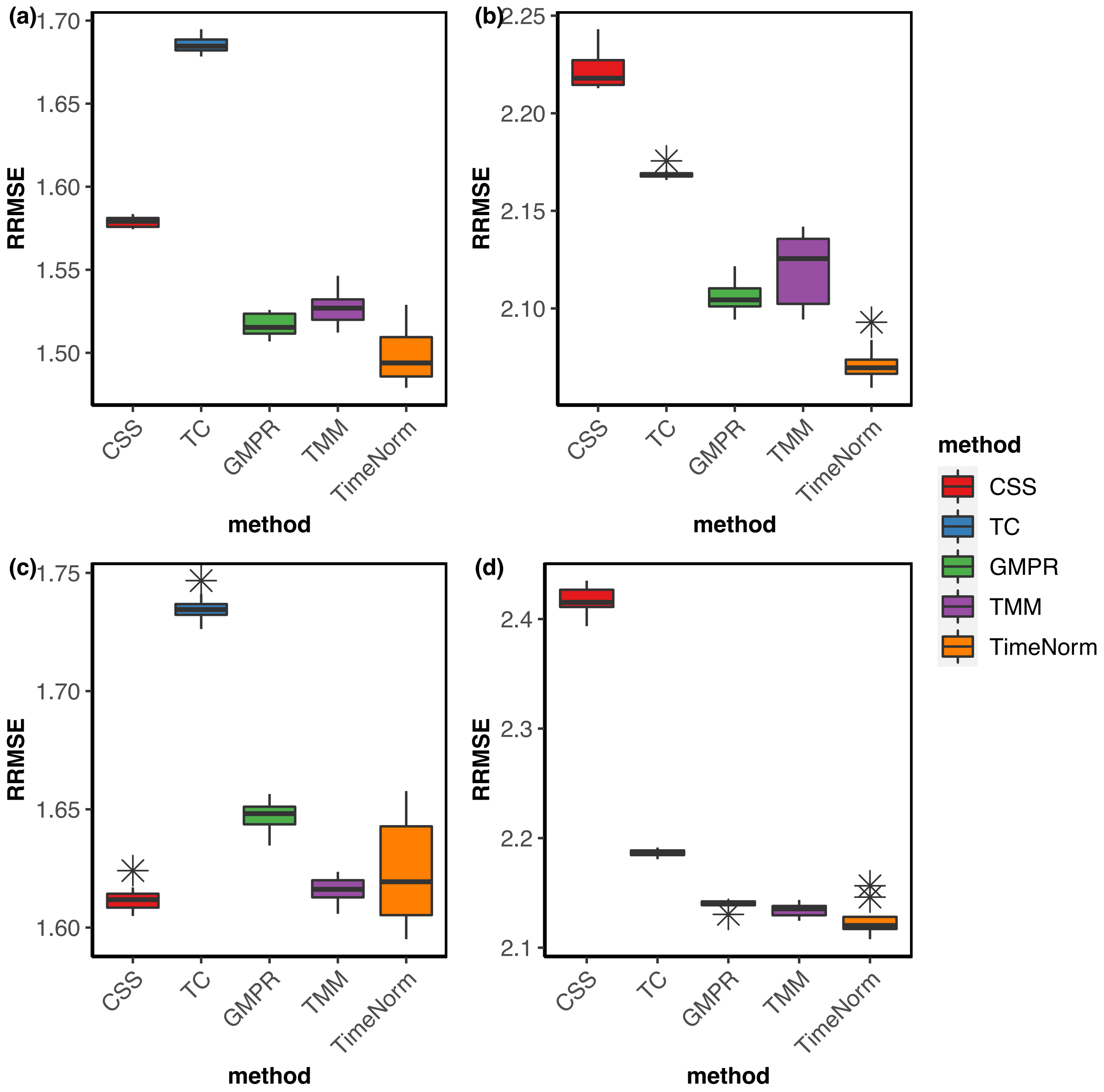

S17: Boxplots of Relative Root Mean Square Error for comparison of different normalization methods based on ten replicated simulations for (a)Test 1A; (b)Test 2A; (c)Test 3A; and (d)Test 4A. The short error bars represent the standard deviation from ten replications.

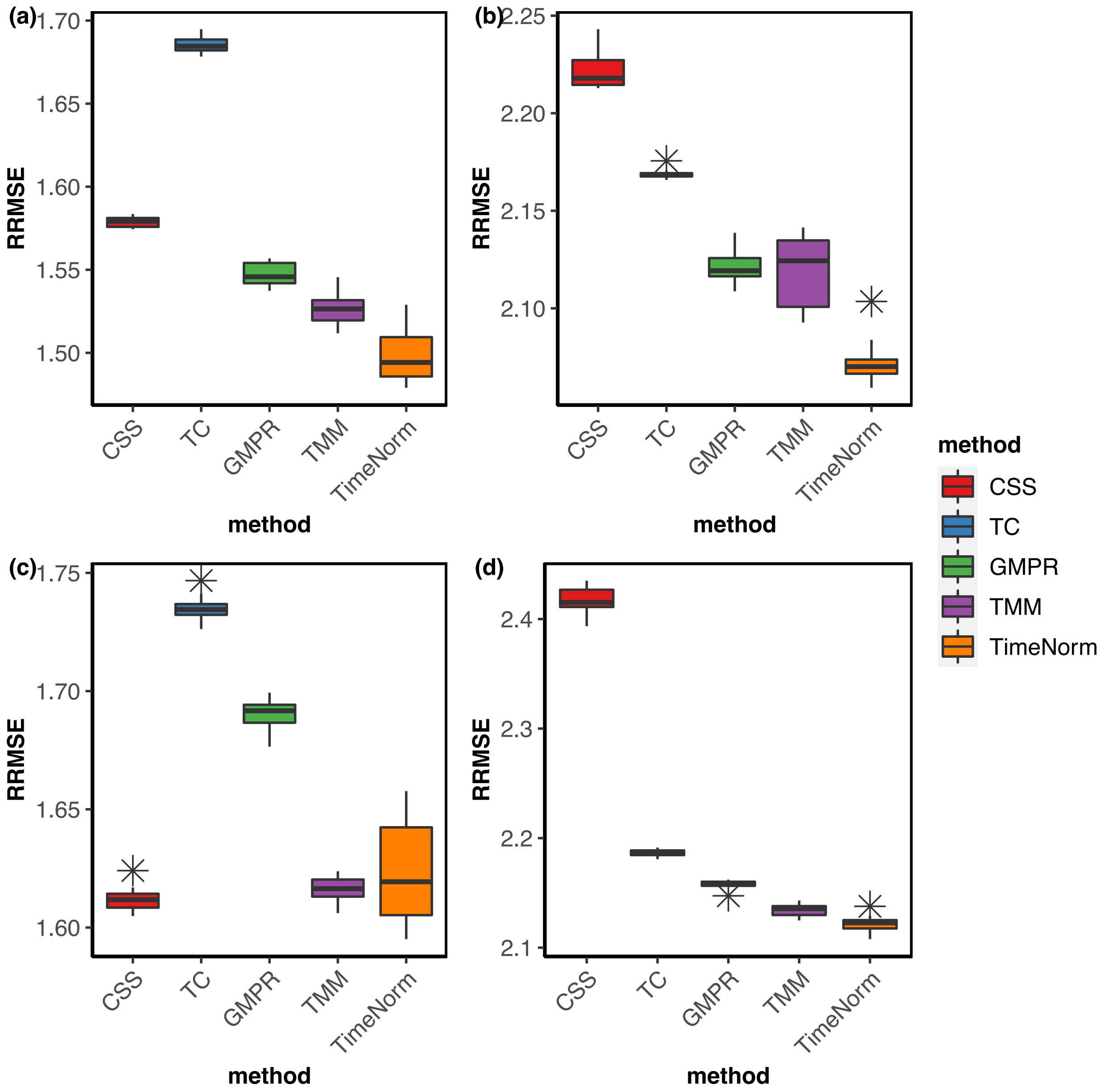

S18: Boxplots of Relative Root Mean Square Error for comparison of different normalization methods based on ten replicated simulations for (a)Test 1B; (b)Test 2B; (c)Test 3B; and (d)Test 4B. The short error bars represent the standard deviation from ten replications.

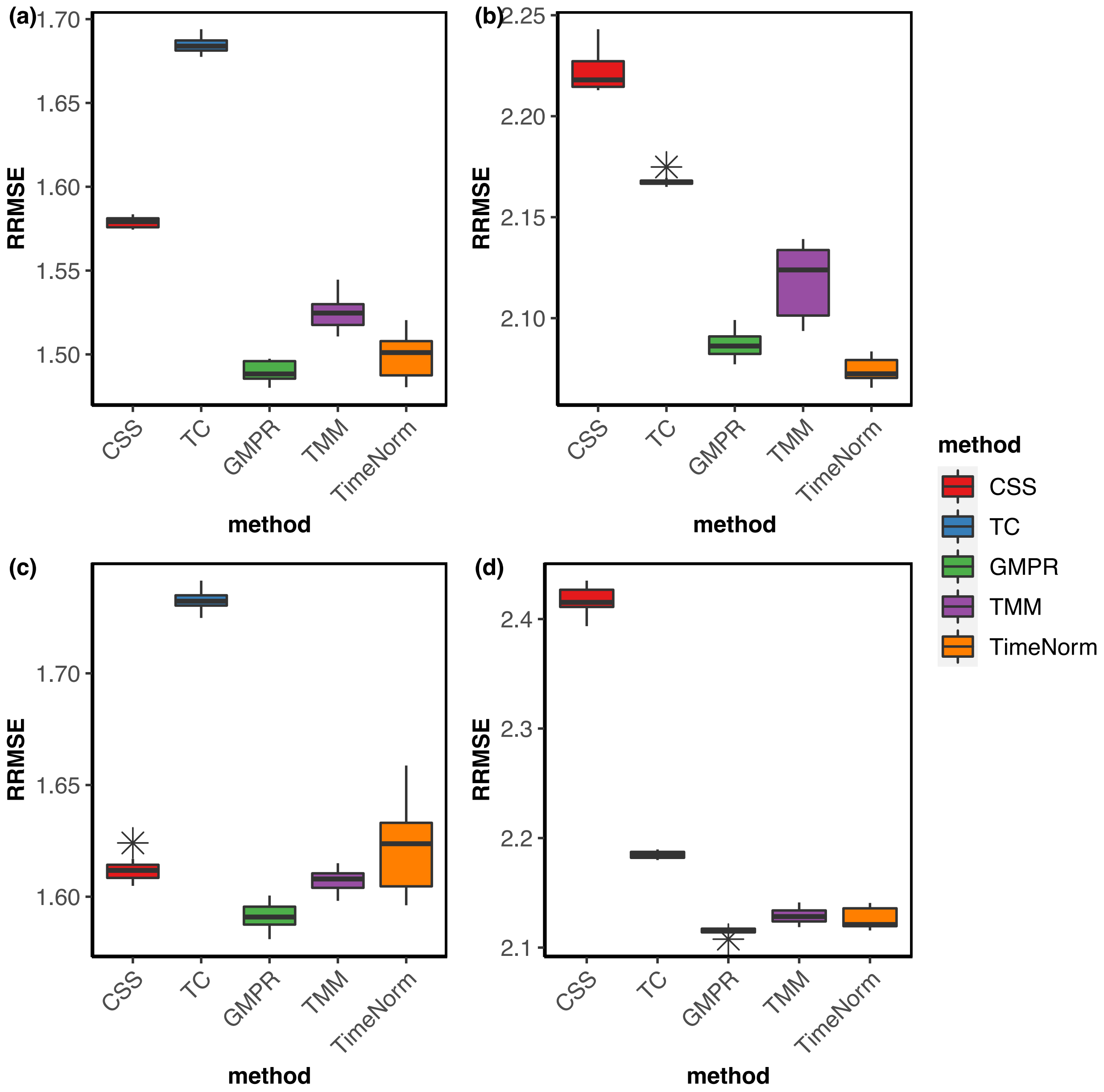

S19: Boxplots of Relative Root Mean Square Error for comparison of different normalization methods based on ten replicated simulations for (a)Test 1C; (b)Test 2C; (c)Test 3C; and (d)Test 4C. The short error bars represent the standard deviation from ten replications.

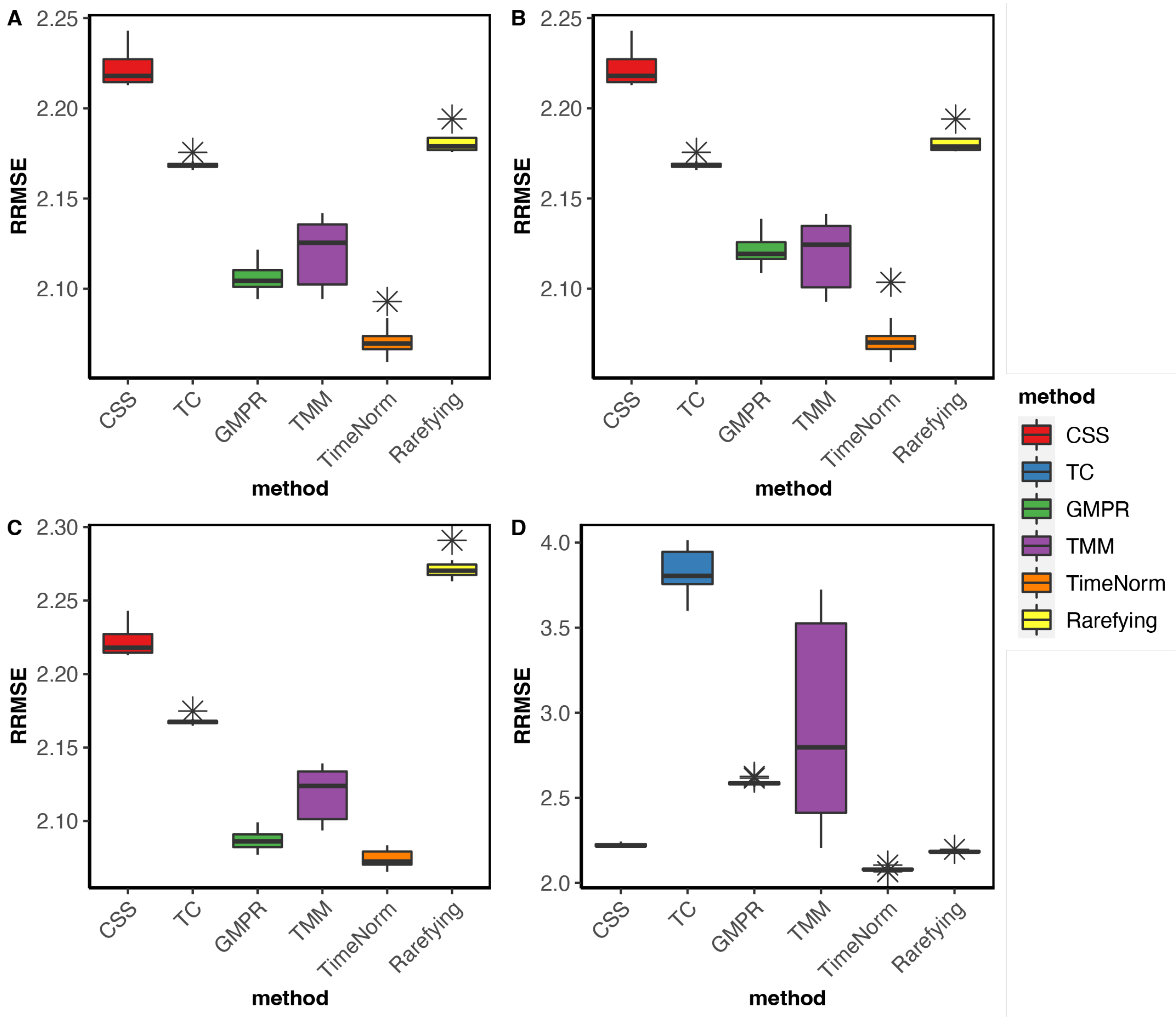

S20: Boxplots of Relative Root Mean Square Error for comparison of different normalization methods (CSS, TC, GMPR, TMM, TimeNorm, and Rarefying) based on ten replicated simulations for (a)Test 2A; (b)Test 2B; (c)Test 2C; and (d)Test 2D. The short error bars represent the standard deviation from ten replications.

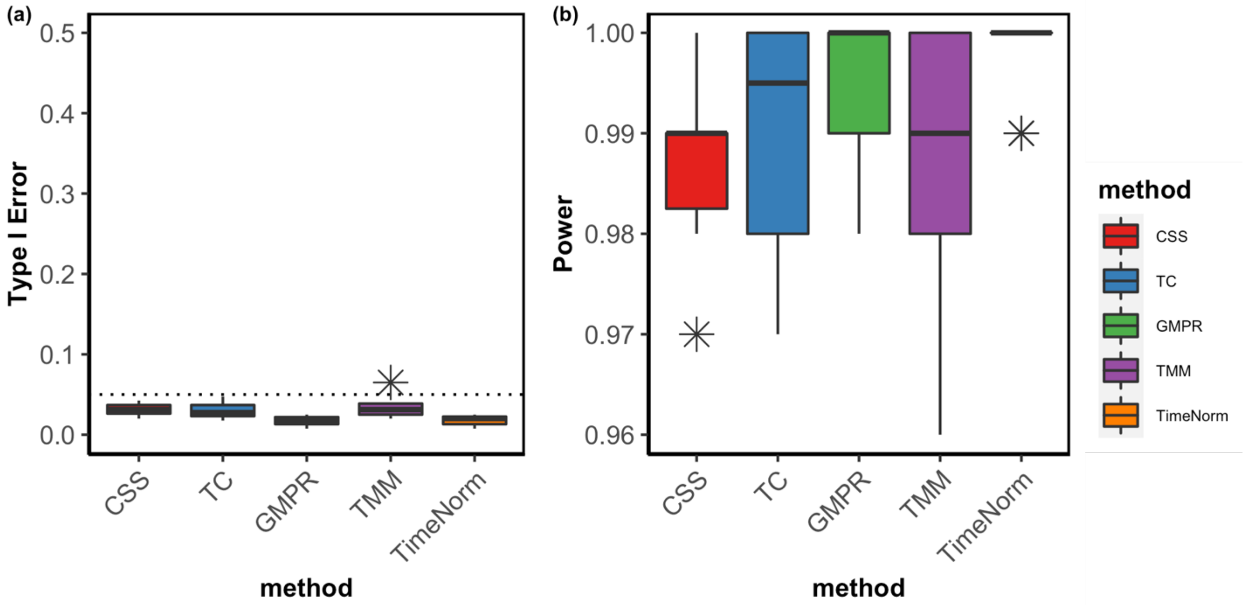

S21: Boxplots of Type I Error and power of DA analysis using metaDprof for the raw count and different normalization methods based on ten replicated simulations for **Test 1A**. The short error bars represent the standard deviation from ten replications.

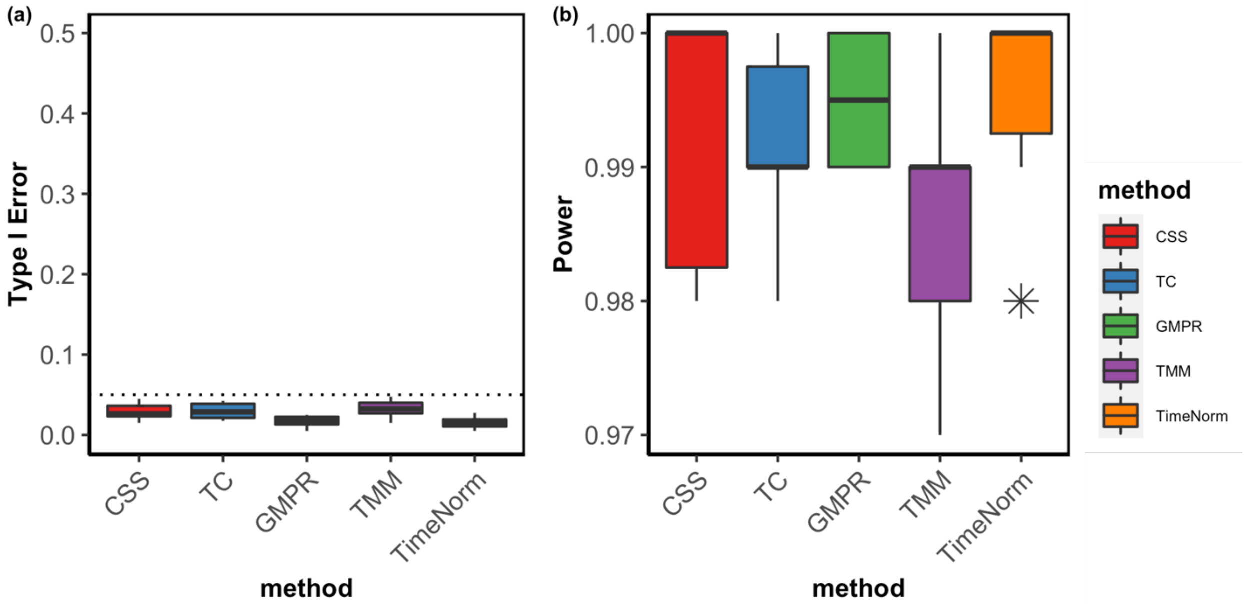

S22: Boxplots of Type I Error and power of DA analysis using metaDprof for the raw count and different normalization methods based on ten replicated simulations for **Test 1B**. The short error bars represent the standard deviation from ten replications.

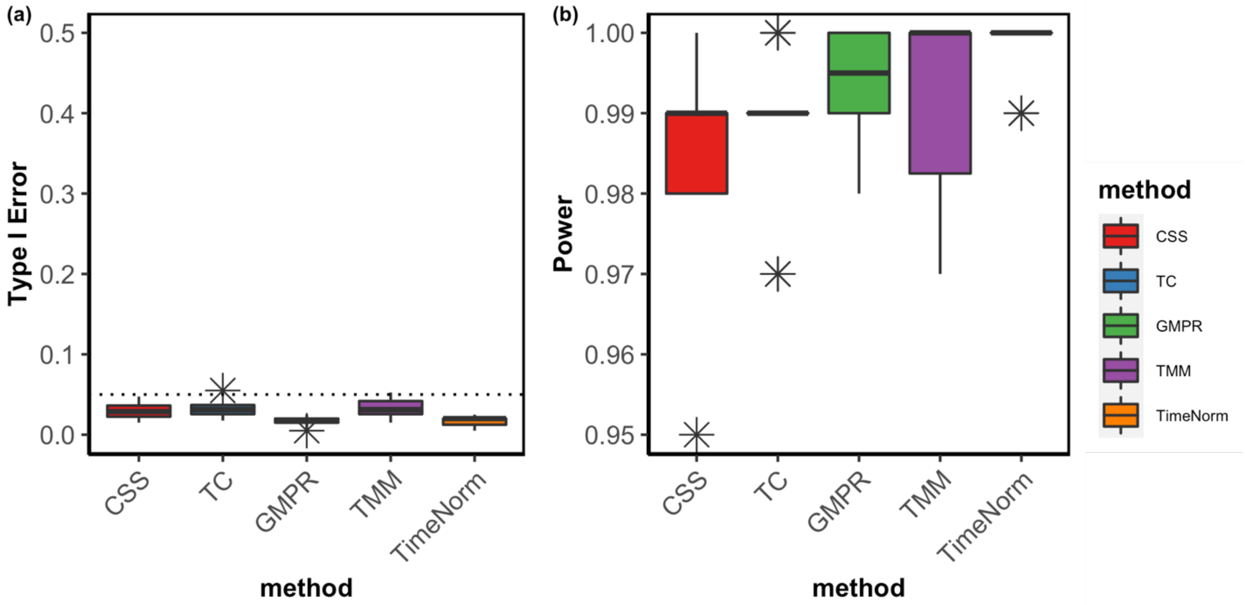

S23: Boxplots of Type I Error and power of DA analysis using metaDprof for the raw count and different normalization methods based on ten replicated simulations for **Test 1C**. The short error bars represent the standard deviation from ten replications.

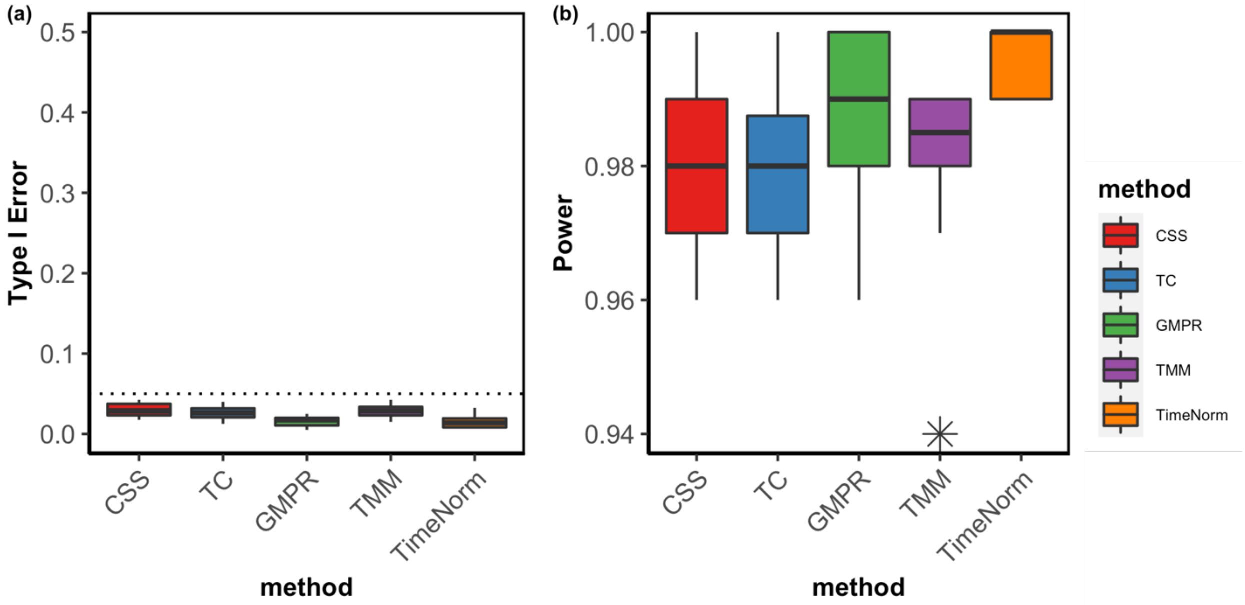

S24: Boxplots of Type I Error and power of DA analysis using metaDprof for the raw count and different normalization methods based on ten replicated simulations for **Test 1D**. The short error bars represent the standard deviation from ten replications.

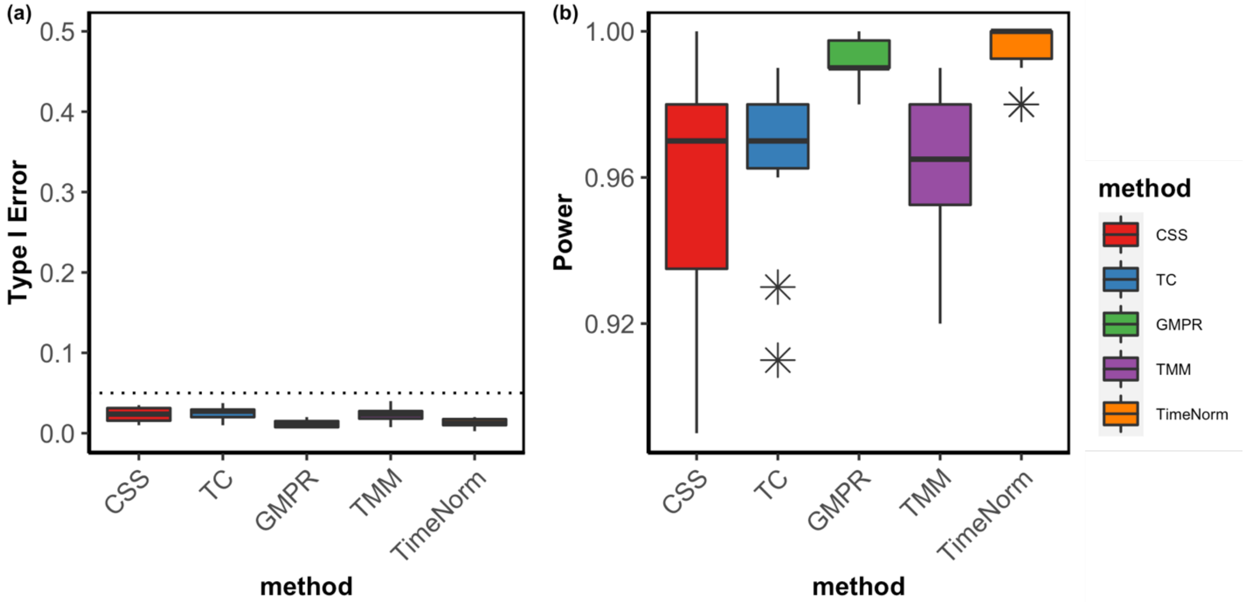

S25: Boxplots of Type I Error and power of DA analysis using metaDprof for the raw count and different normalization methods based on ten replicated simulations for **Test 2A**. The short error bars represent the standard deviation from ten replications.

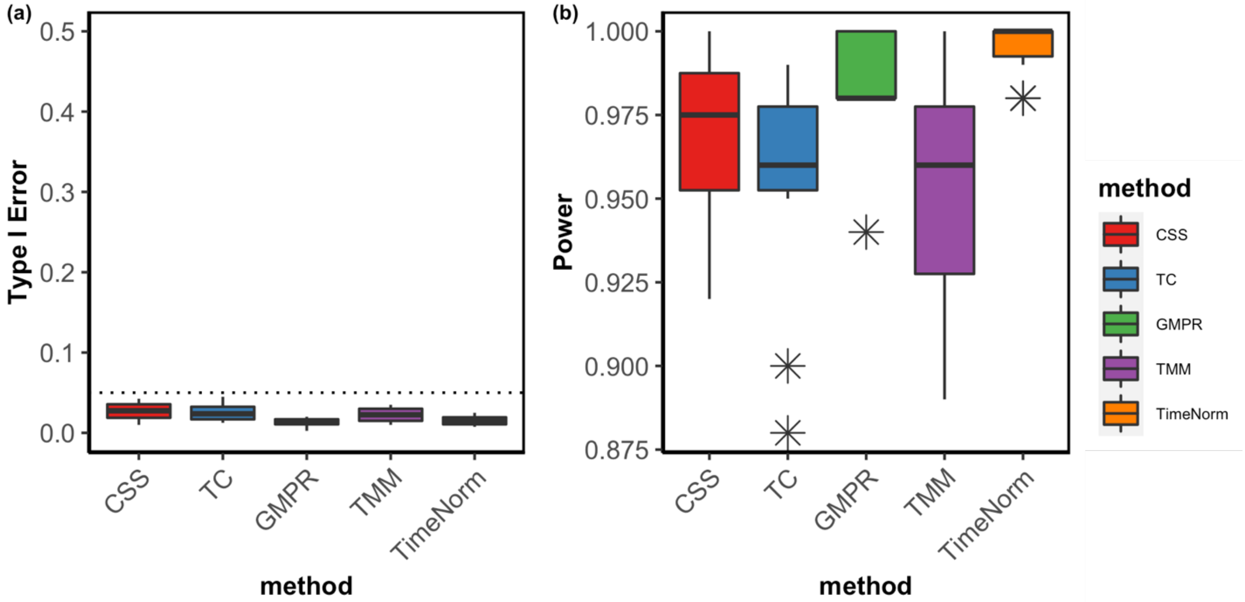

S26: Boxplots of Type I Error and power of DA analysis using metaDprof for the raw count and different normalization methods based on ten replicated simulations for **Test 2B**. The short error bars represent the standard deviation from ten replications.

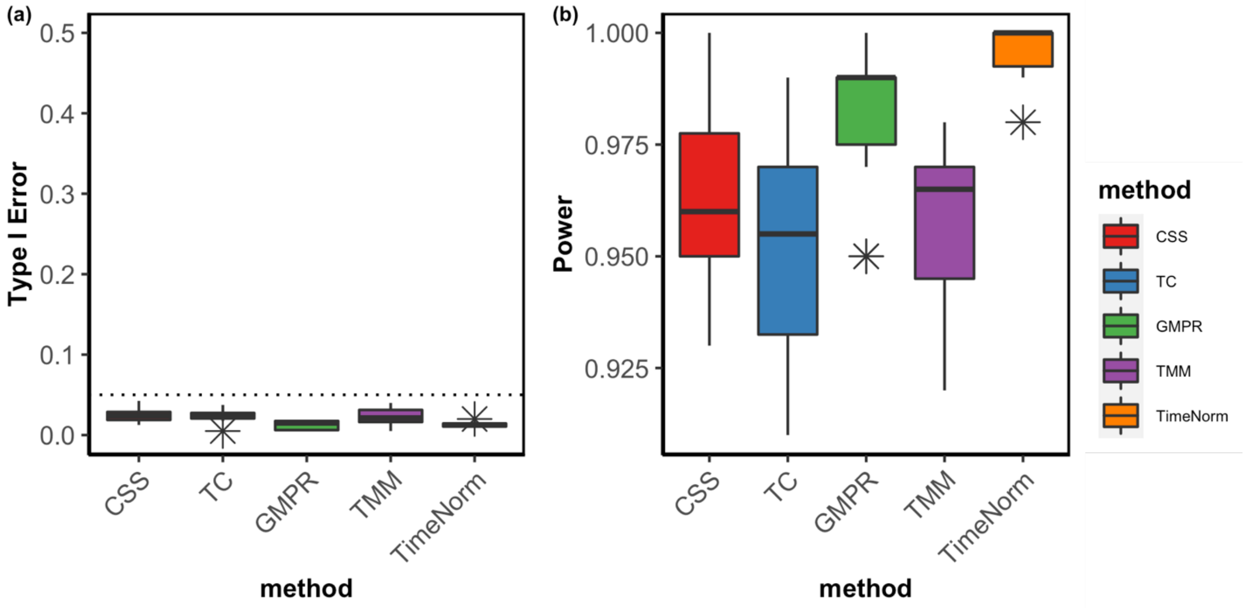

S27: Boxplots of Type I Error and power of DA analysis using metaDprof for the raw count and different normalization methods based on ten replicated simulations for **Test 2C**. The short error bars represent the standard deviation from ten replications.

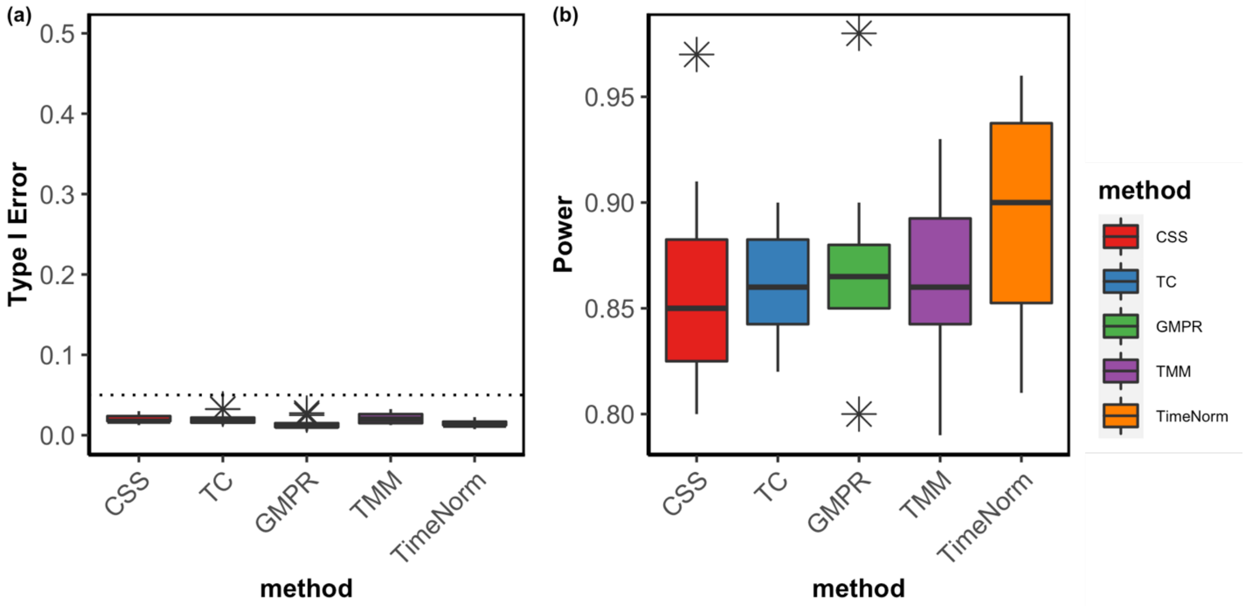

S28: Boxplots of Type I Error and power of DA analysis using metaDprof for the raw count and different normalization methods based on ten replicated simulations for **Test 3A**. The short error bars represent the standard deviation from ten replications.

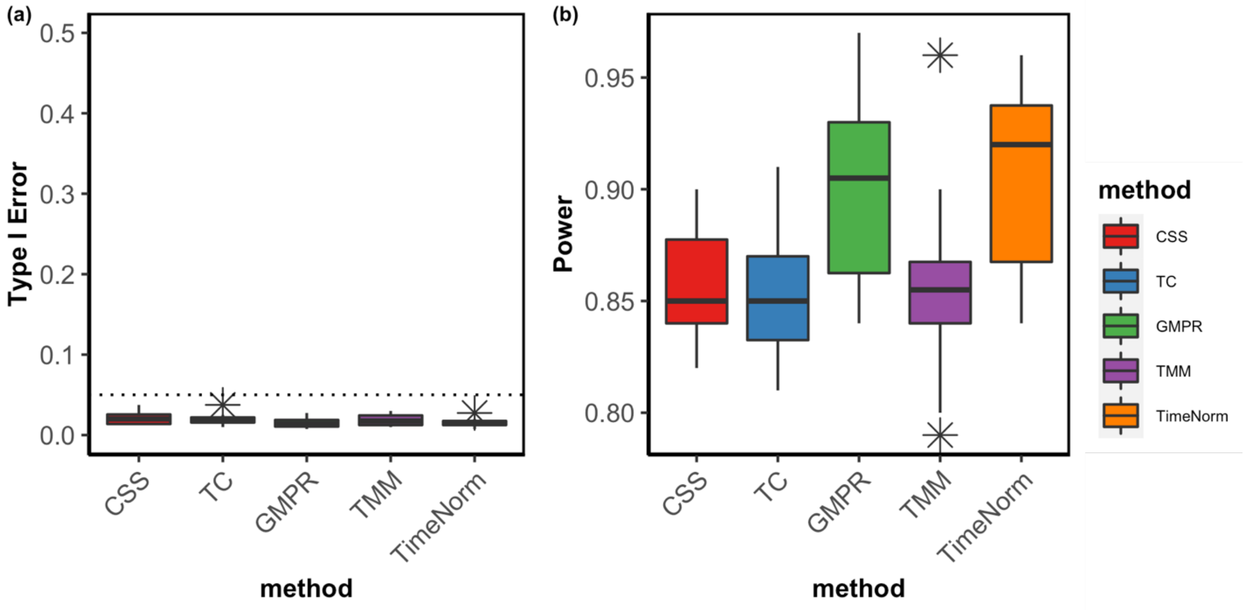

S29: Boxplots of Type I Error and power of DA analysis using metaDprof for the raw count and different normalization methods based on ten replicated simulations for **Test 3B**. The short error bars represent the standard deviation from ten replications.

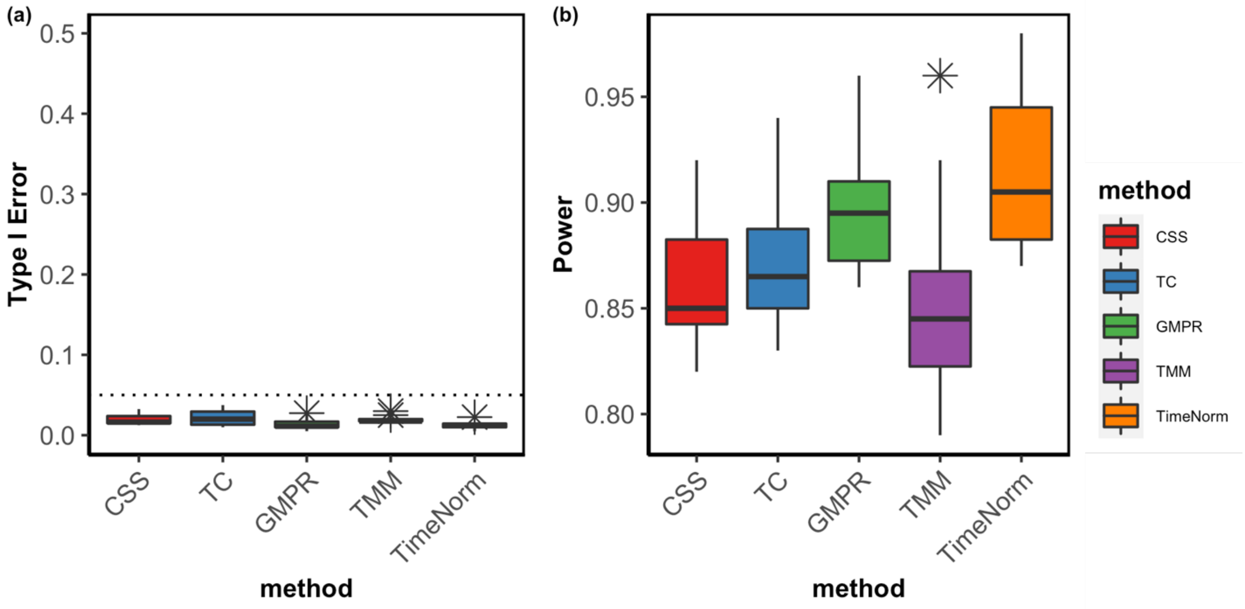

S30: Boxplots of Type I Error and power of DA analysis using metaDprof for the raw count and different normalization methods based on ten replicated simulations for **Test 3C**. The short error bars represent the standard deviation from ten replications.

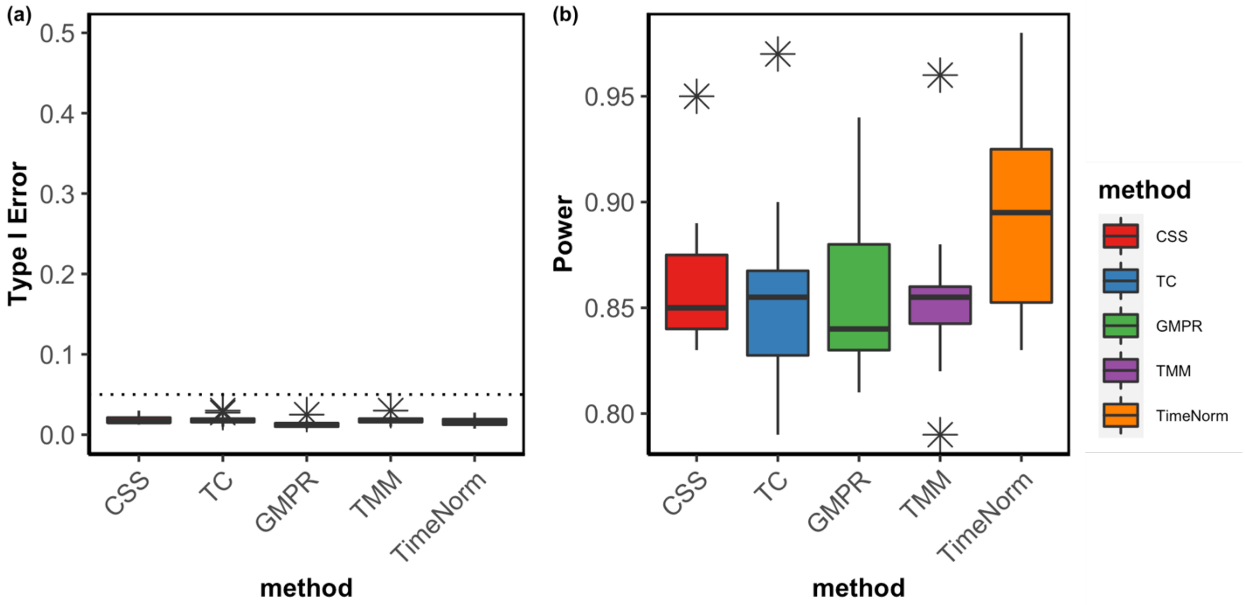

S31: Boxplots of Type I Error and power of DA analysis using metaDprof for the raw count and different normalization methods based on ten replicated simulations for **Test 3D**. The short error bars represent the standard deviation from ten replications.

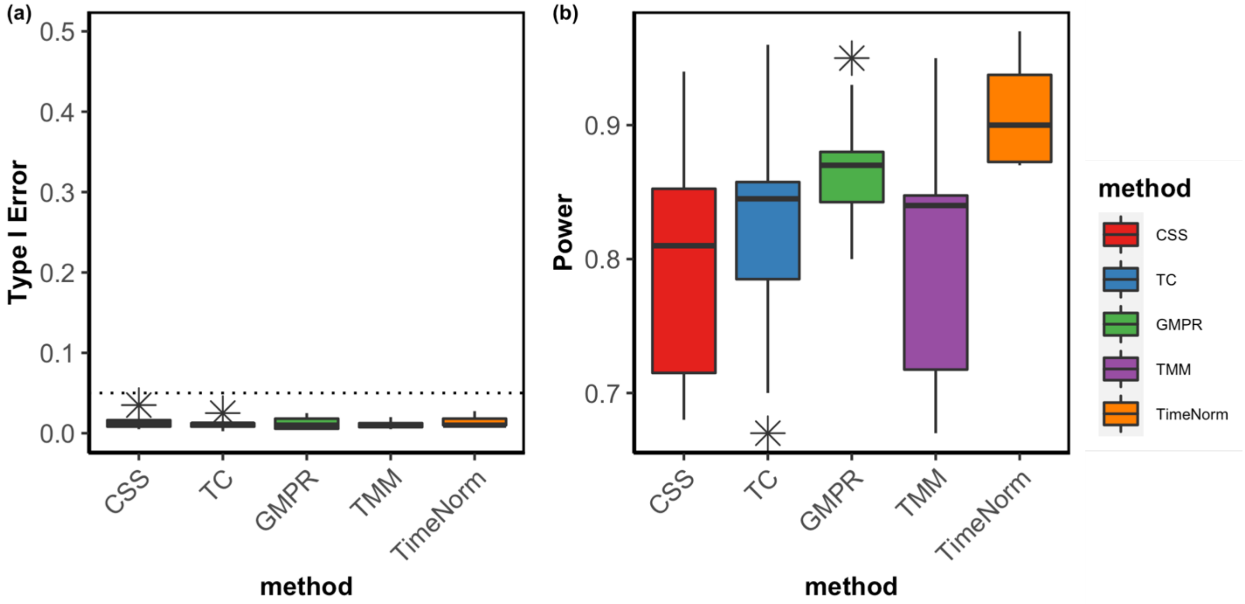

S32: Boxplots of Type I Error and power of DA analysis using metaDprof for the raw count and different normalization methods based on ten replicated simulations for **Test 4A**. The short error bars represent the standard deviation from ten replications.

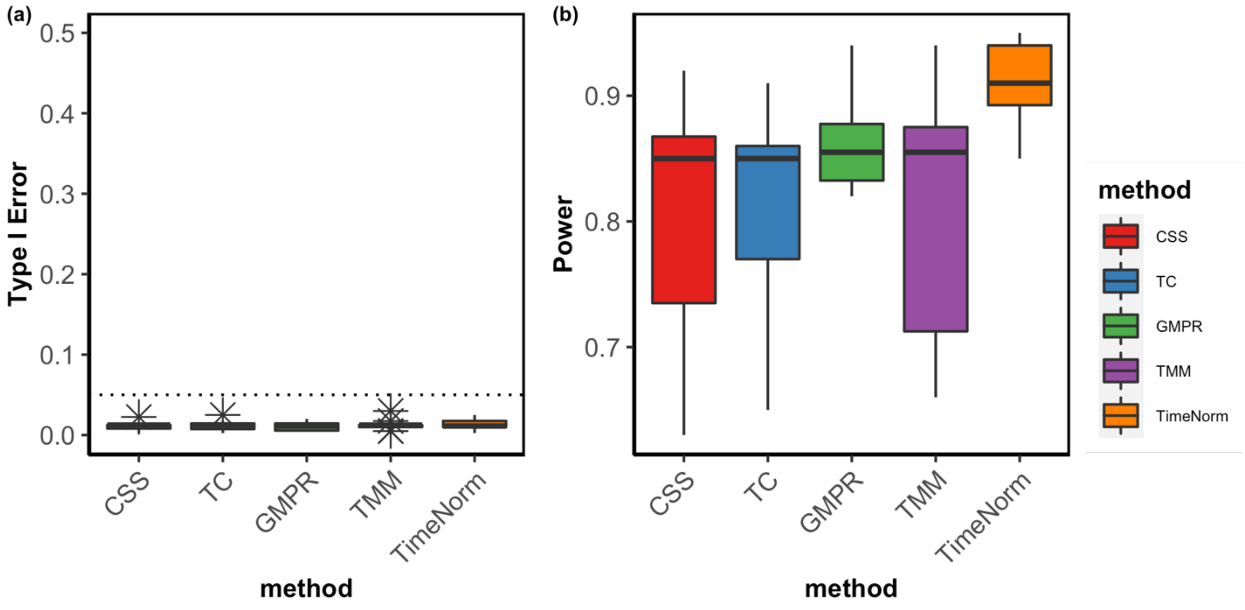

S33: Boxplots of Type I Error and power of DA analysis using metaDprof for the raw count and different normalization methods based on ten replicated simulations for **Test 4B**. The short error bars represent the standard deviation from ten replications.

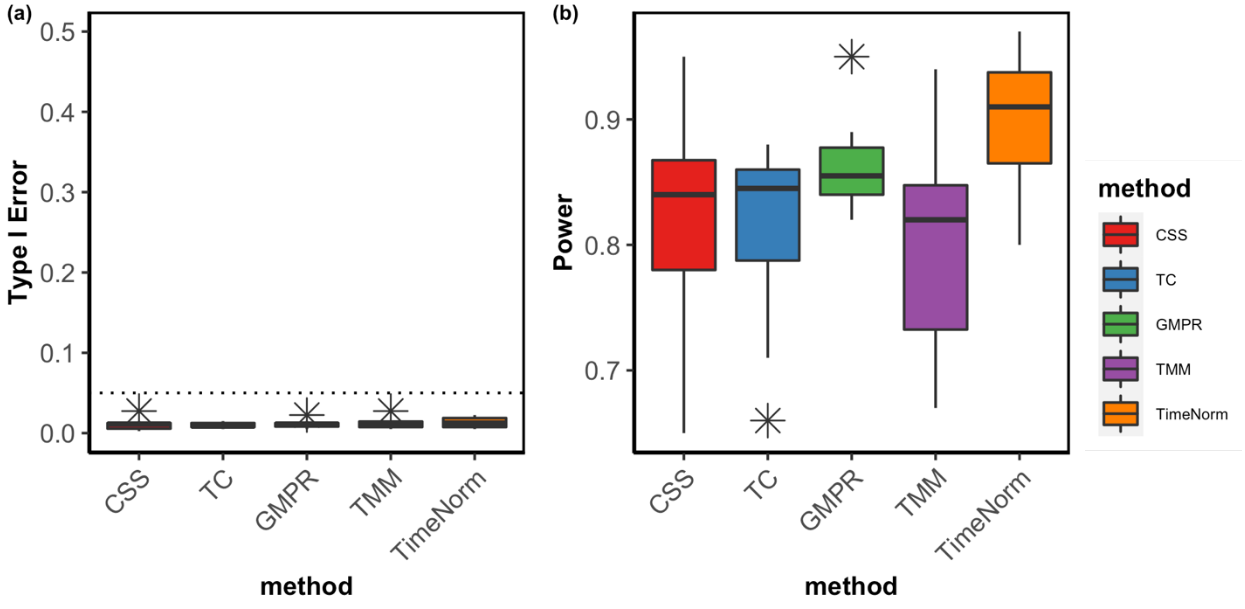

S34: Boxplots of Type I Error and power of DA analysis using metaDprof for the raw count and different normalization methods based on ten replicated simulations for **Test 4C**. The short error bars represent the standard deviation from ten replications.

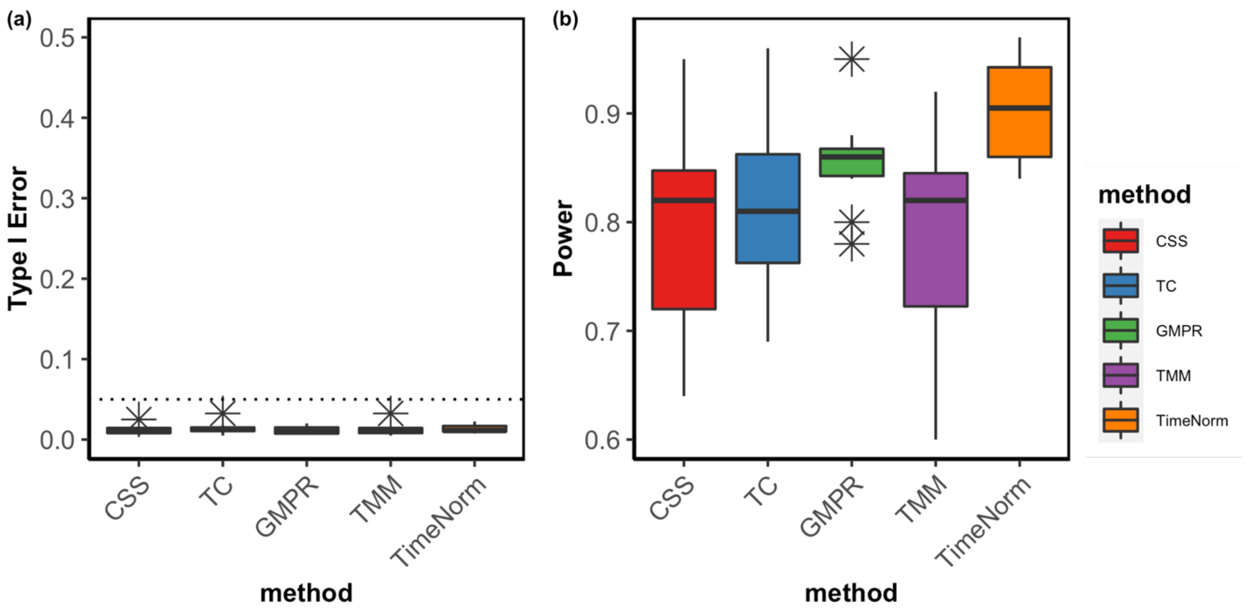

S35: Boxplots of Type I Error and power of DA analysis using metaDprof for the raw count and different normalization methods based on ten replicated simulations for **Test 4D**. The short error bars represent the standard deviation from ten replications.

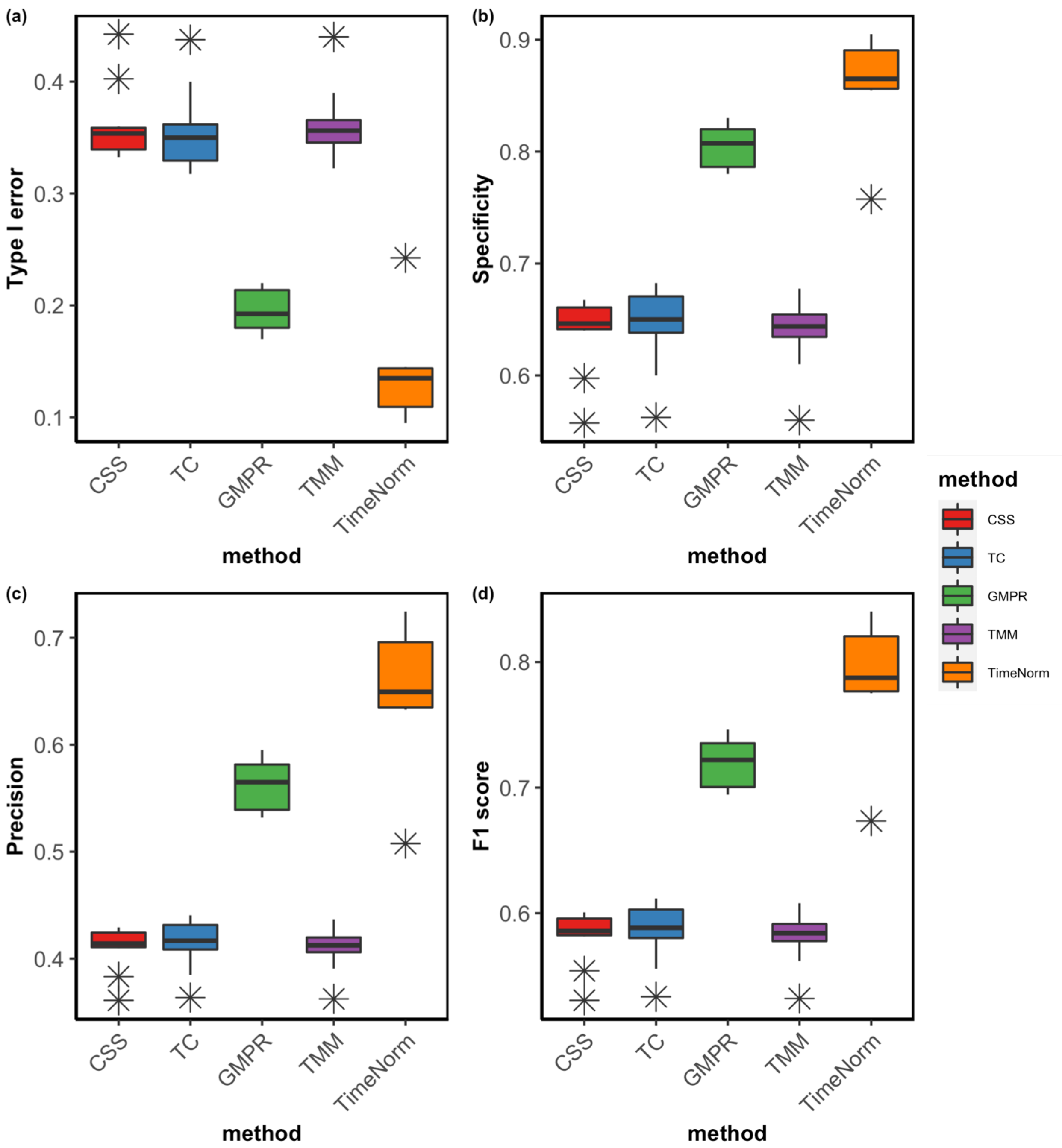

S36: Boxplots of Type I error, Specificity, Precision, F1 score of DA analysis using splinectomeR for different normalization methods based on ten replicated simulations for **Test 1A**.

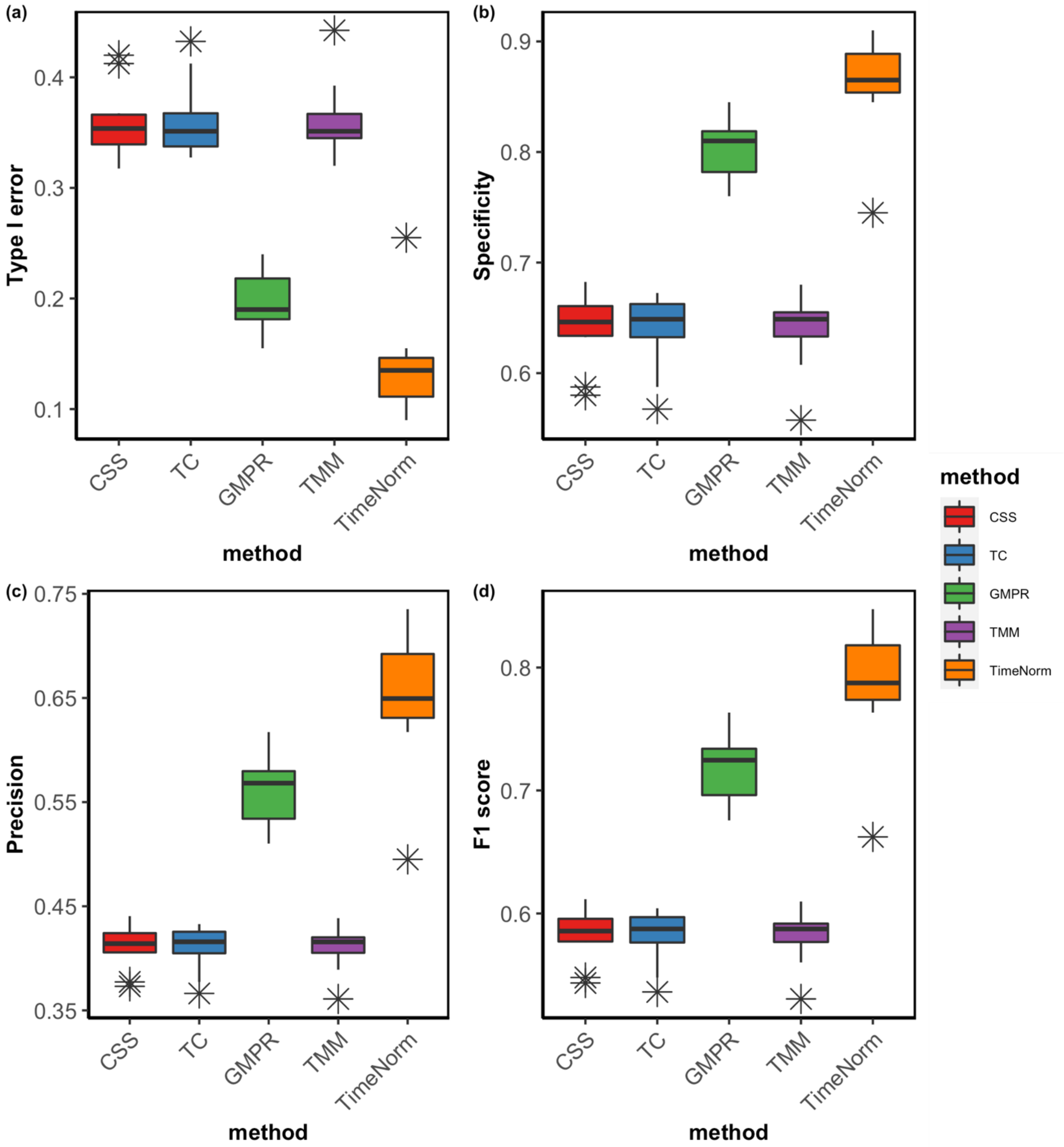

S37: Boxplots of Type I error, Specificity, Precision, F1 score of DA analysis using splinectomeR for different normalization methods based on ten replicated simulations for **Test 1B**.

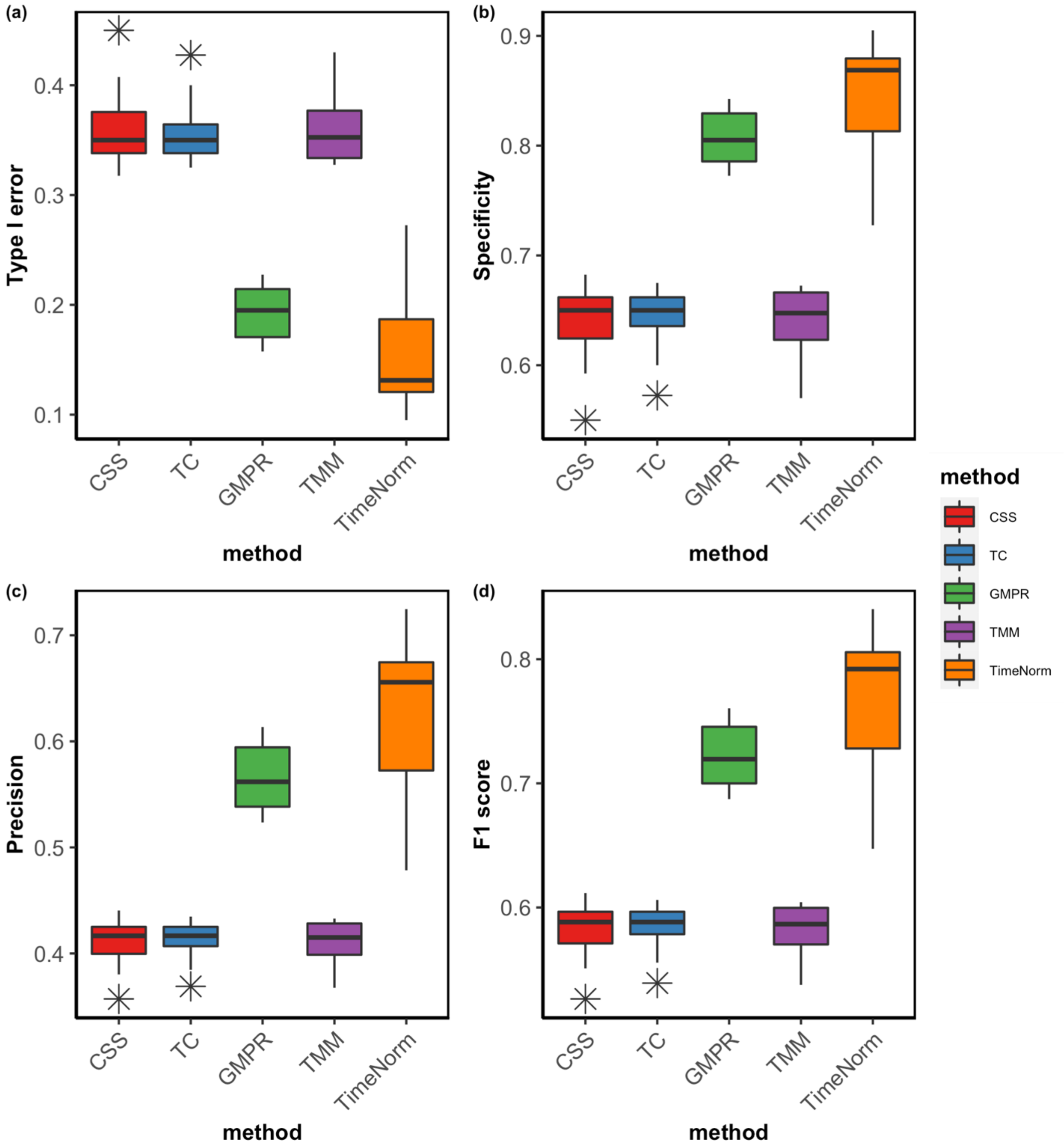

S38: Boxplots of Type I error, Specificity, Precision, F1 score of DA analysis using splinectomeR for different normalization methods based on ten replicated simulations for **Test 1C**.

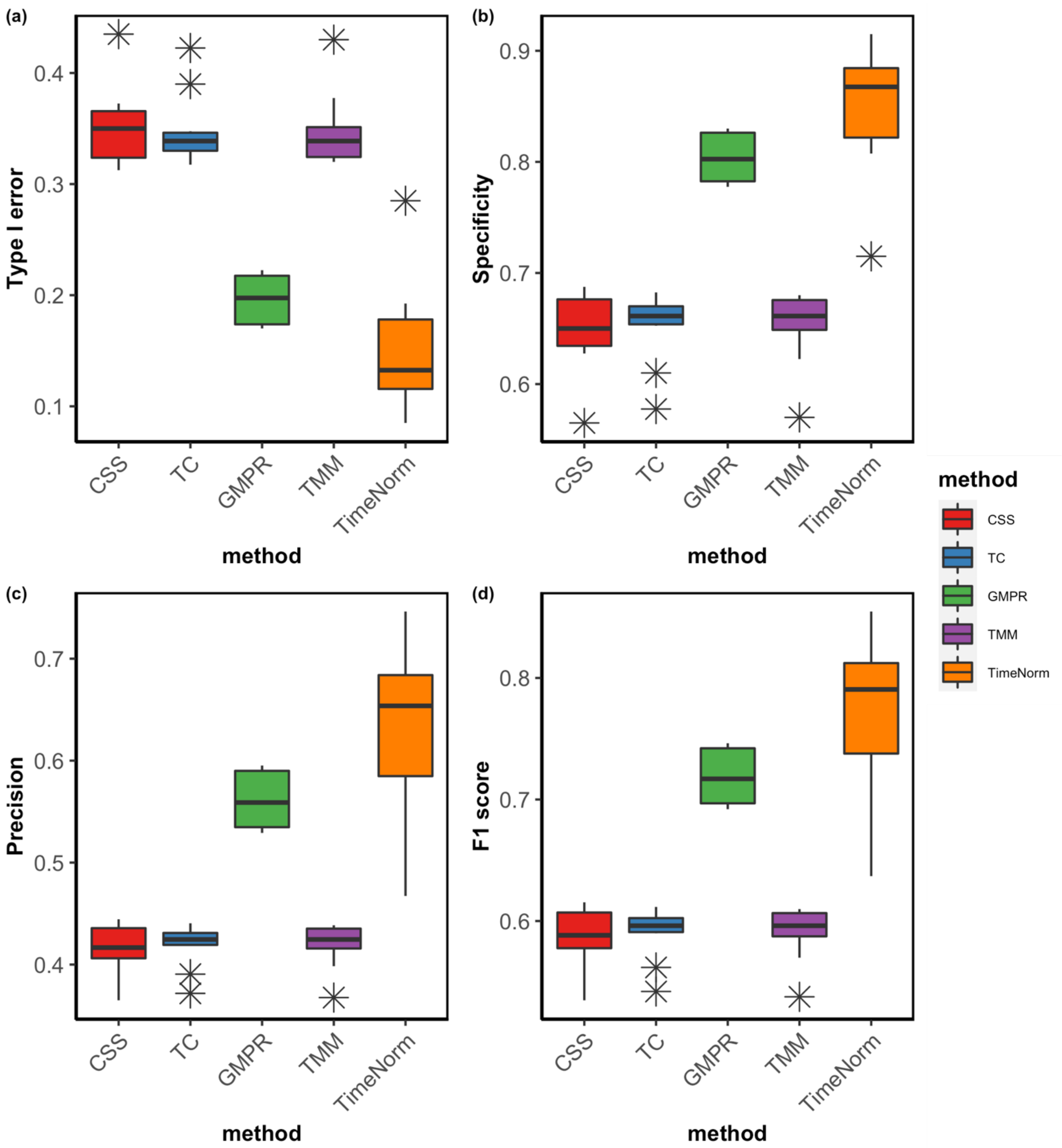

S39: Boxplots of Type I error, Specificity, Precision, F1 score of DA analysis using splinectomeR for different normalization methods based on ten replicated simulations for **Test 1D**.

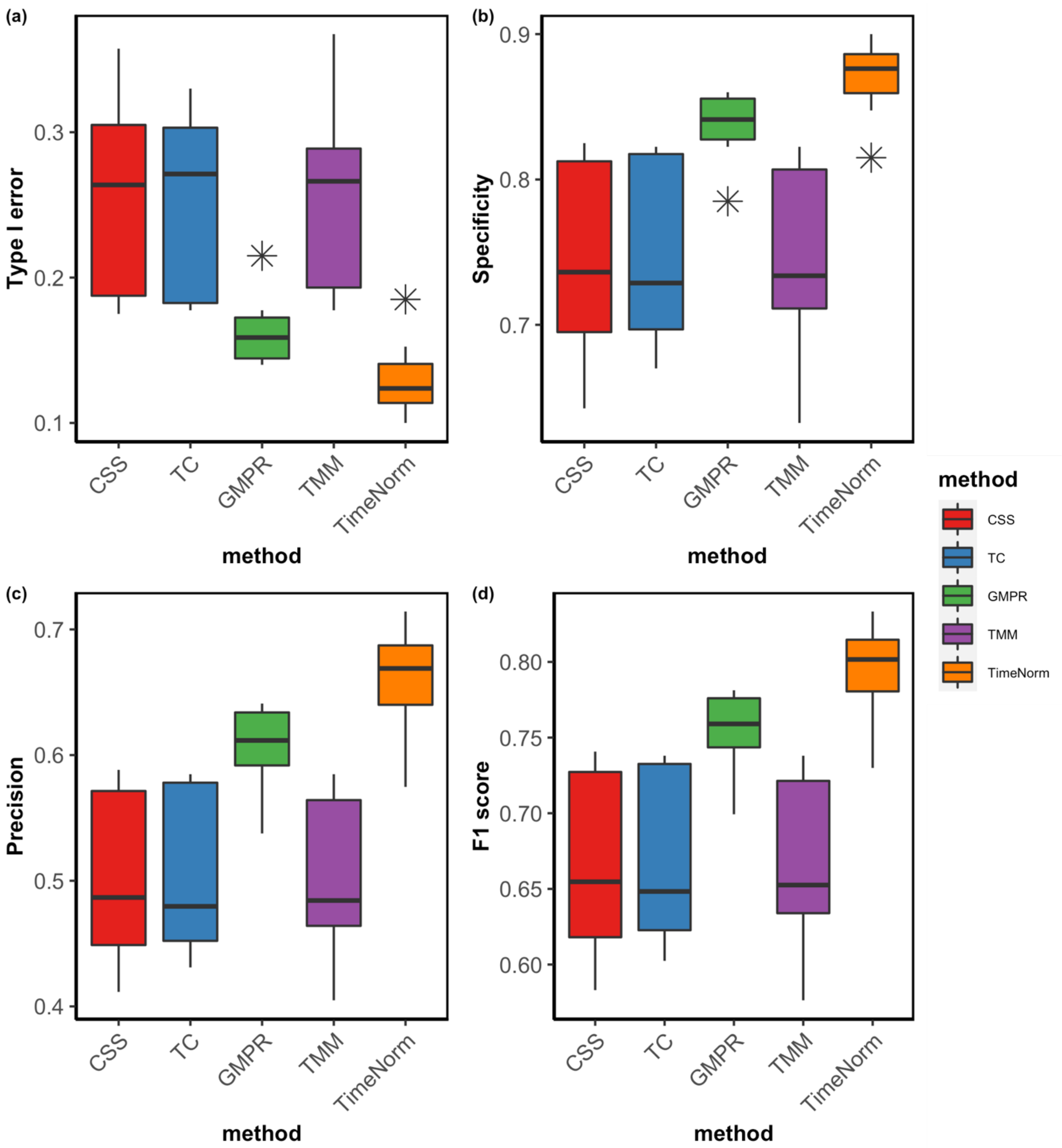

S40: Boxplots of Type I error, Specificity, Precision, F1 score of DA analysis using splinectomeR for different normalization methods based on ten replicated simulations for **Test 2A**.

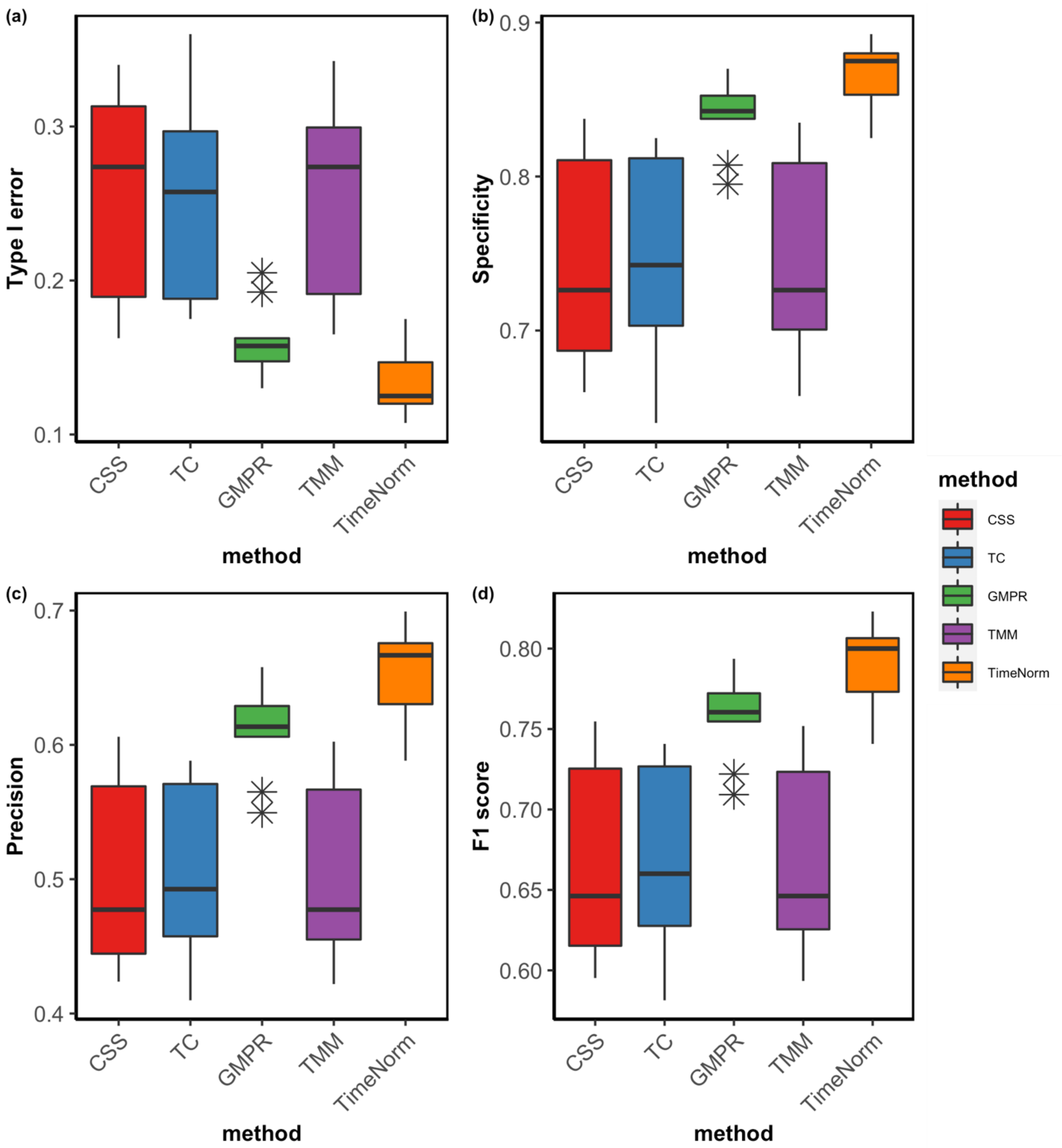

S41: Boxplots of Type I error, Specificity, Precision, F1 score of DA analysis using splinectomeR for different normalization methods based on ten replicated simulations for **Test 2B**.

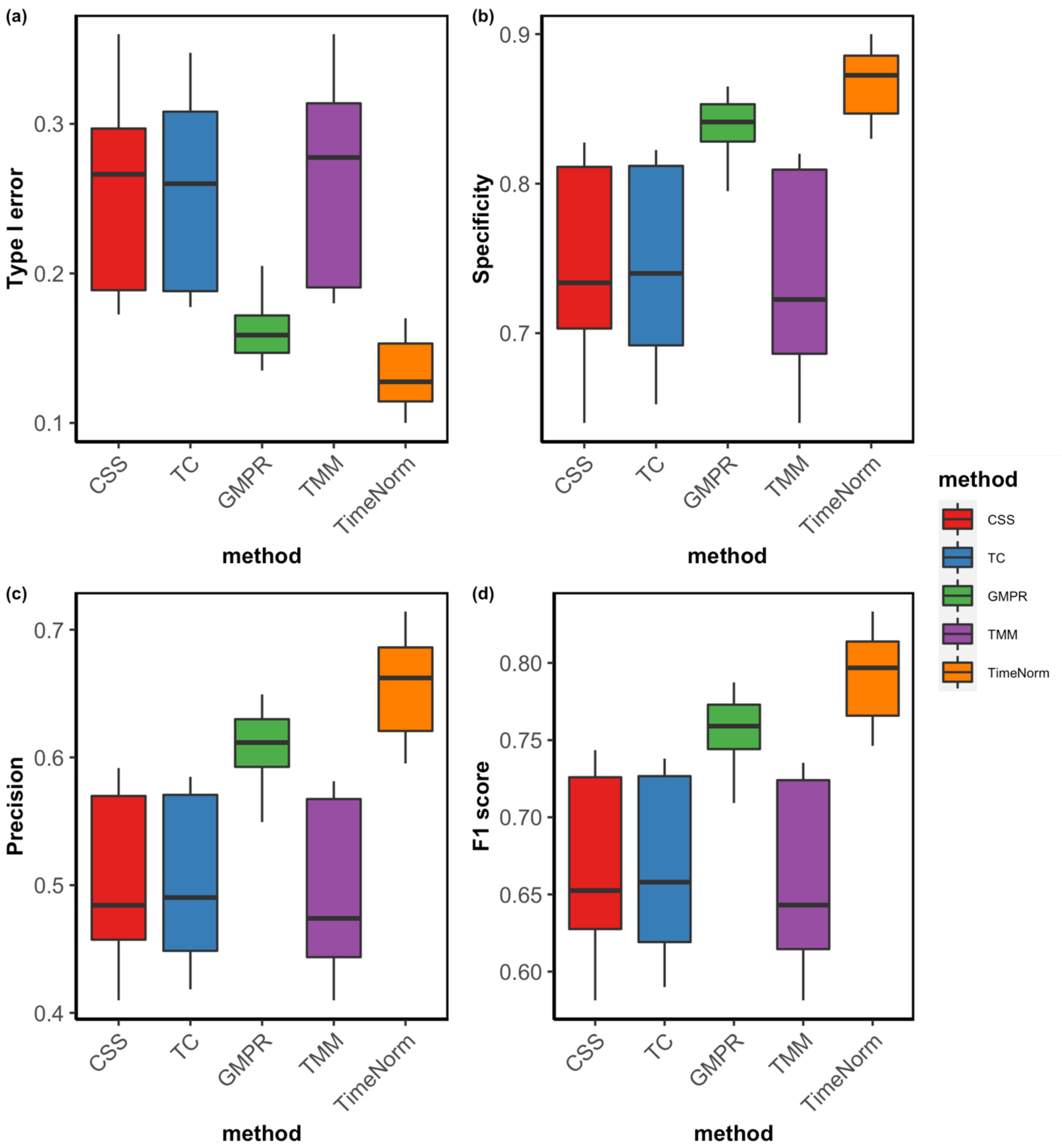

S42: Boxplots of Type I error, Specificity, Precision, F1 score of DA analysis using splinectomeR for different normalization methods based on ten replicated simulations for **Test 2C**.

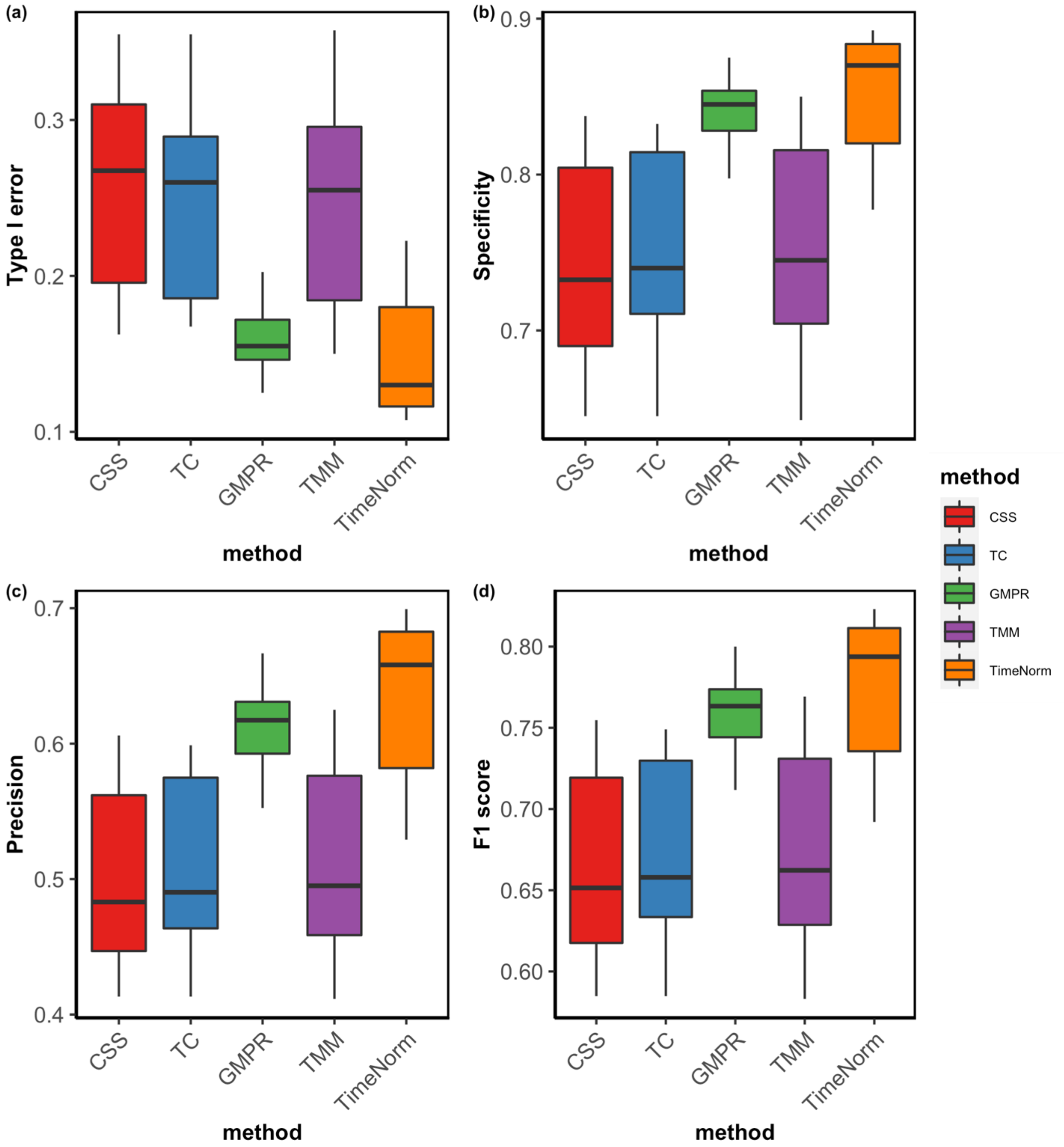

S43: Boxplots of Type I error, Specificity, Precision, F1 score of DA analysis using splinectomeR for different normalization methods based on ten replicated simulations for **Test 2D**.

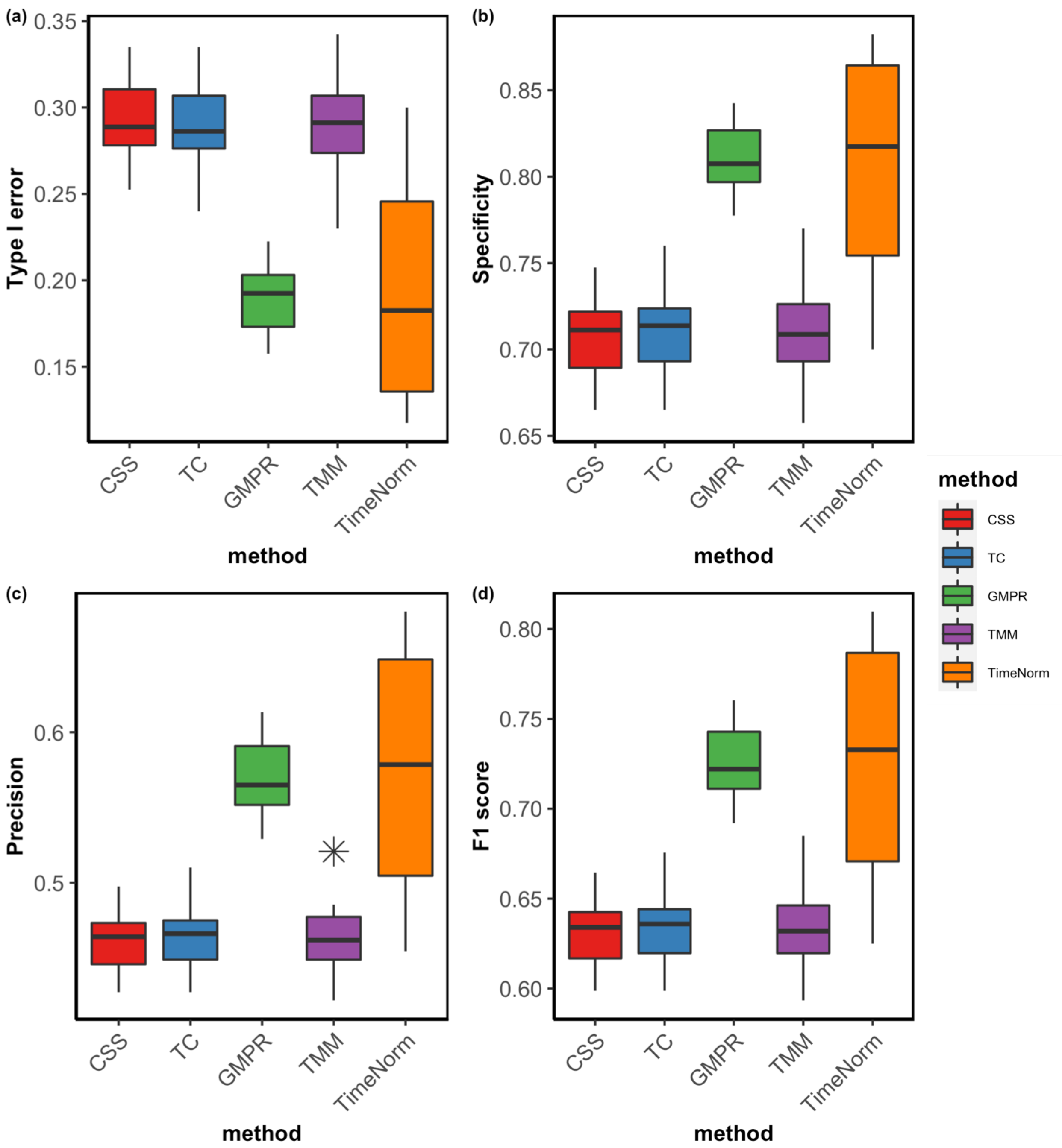

S44: Boxplots of Type I error, Specificity, Precision, F1 score of DA analysis using splinectomeR for different normalization methods based on ten replicated simulations for **Test 3A**.

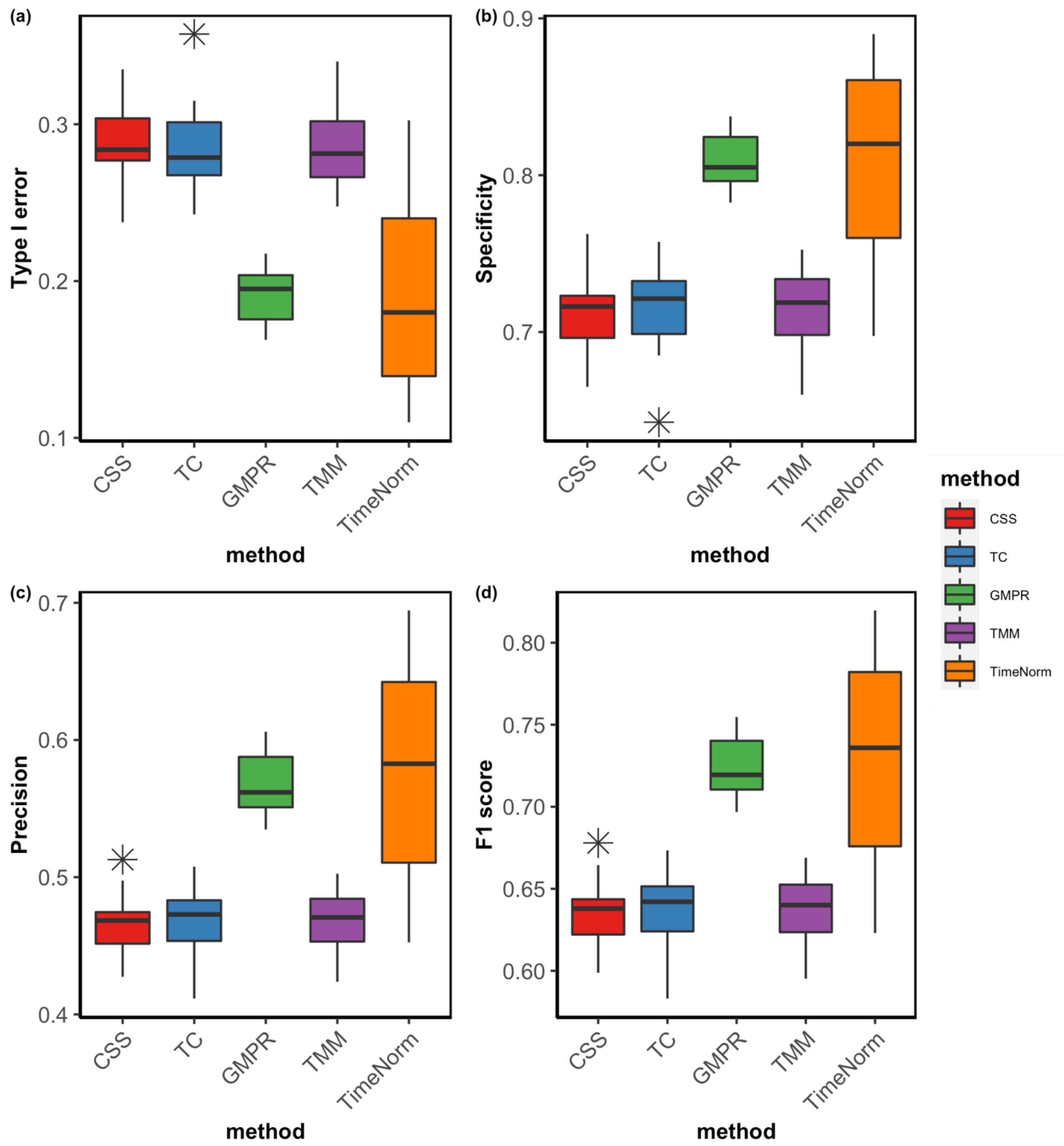

S45: Boxplots of Type I error, Specificity, Precision, F1 score of DA analysis using splinectomeR for different normalization methods based on ten replicated simulations for **Test 3B**.

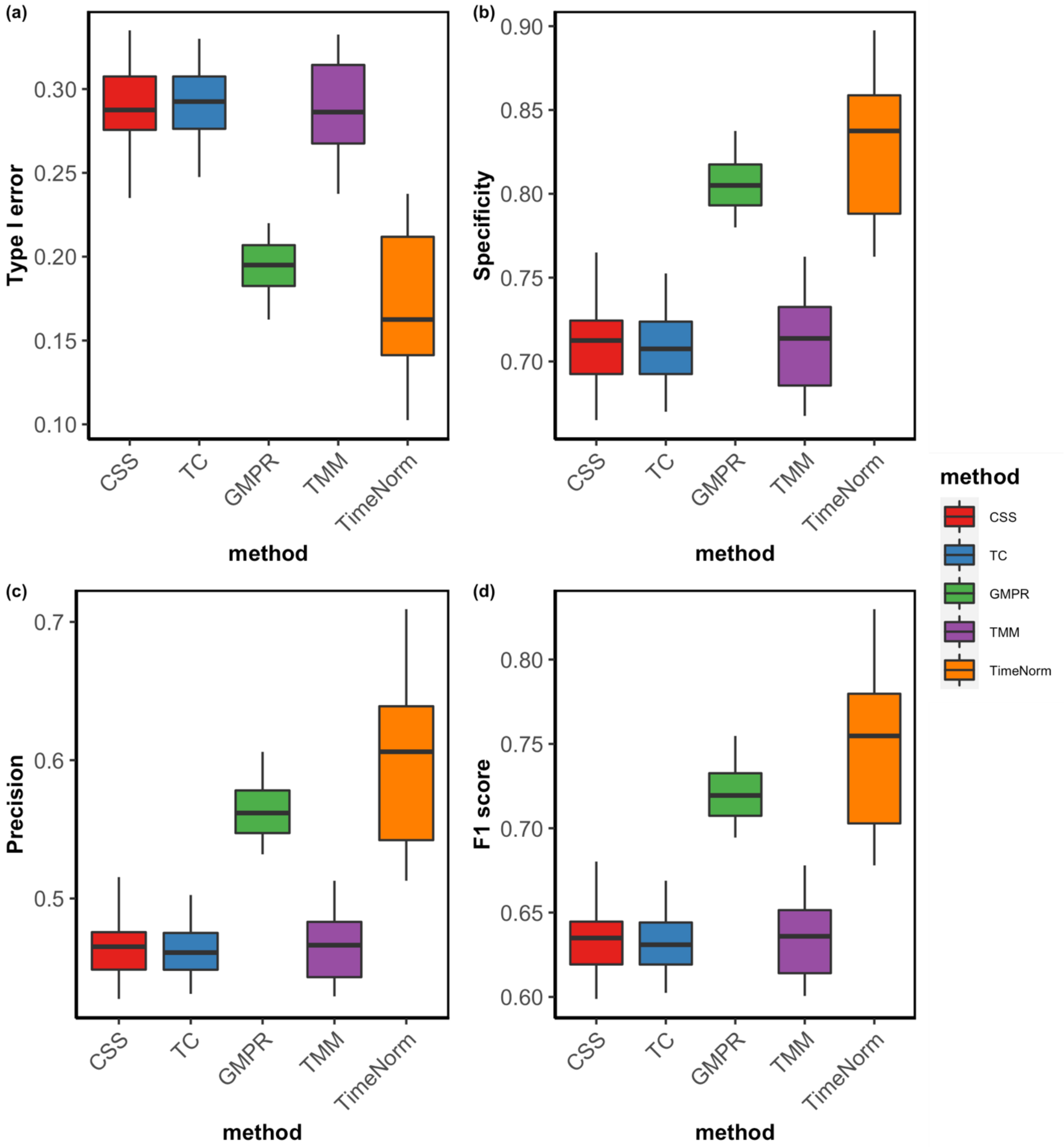

S46: Boxplots of Type I error, Specificity, Precision, F1 score of DA analysis using splinectomeR for different normalization methods based on ten replicated simulations for **Test 3C**.

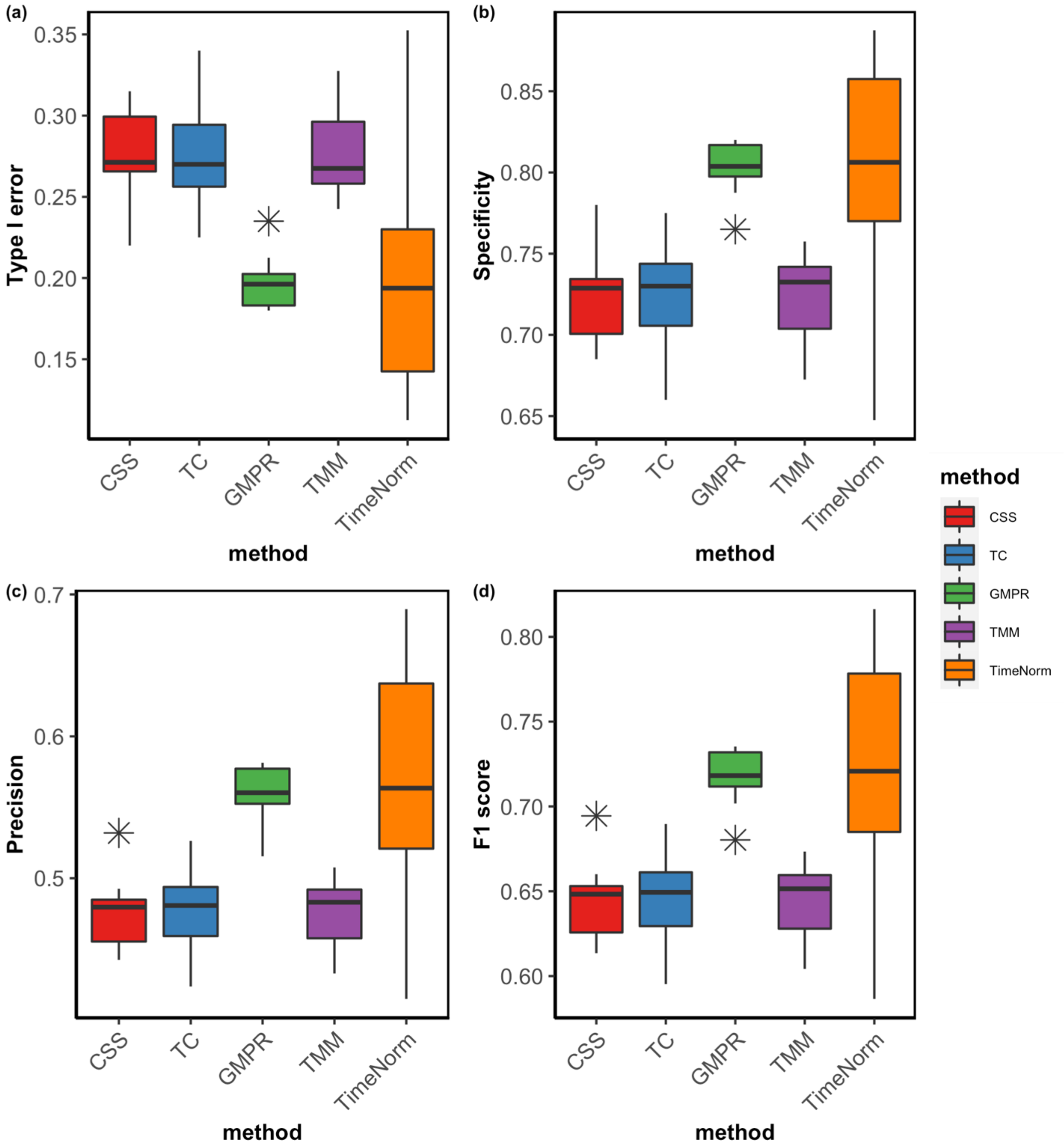

S47: Boxplots of Type I error, Specificity, Precision, F1 score of DA analysis using splinectomeR for different normalization methods based on ten replicated simulations for **Test 3D**.

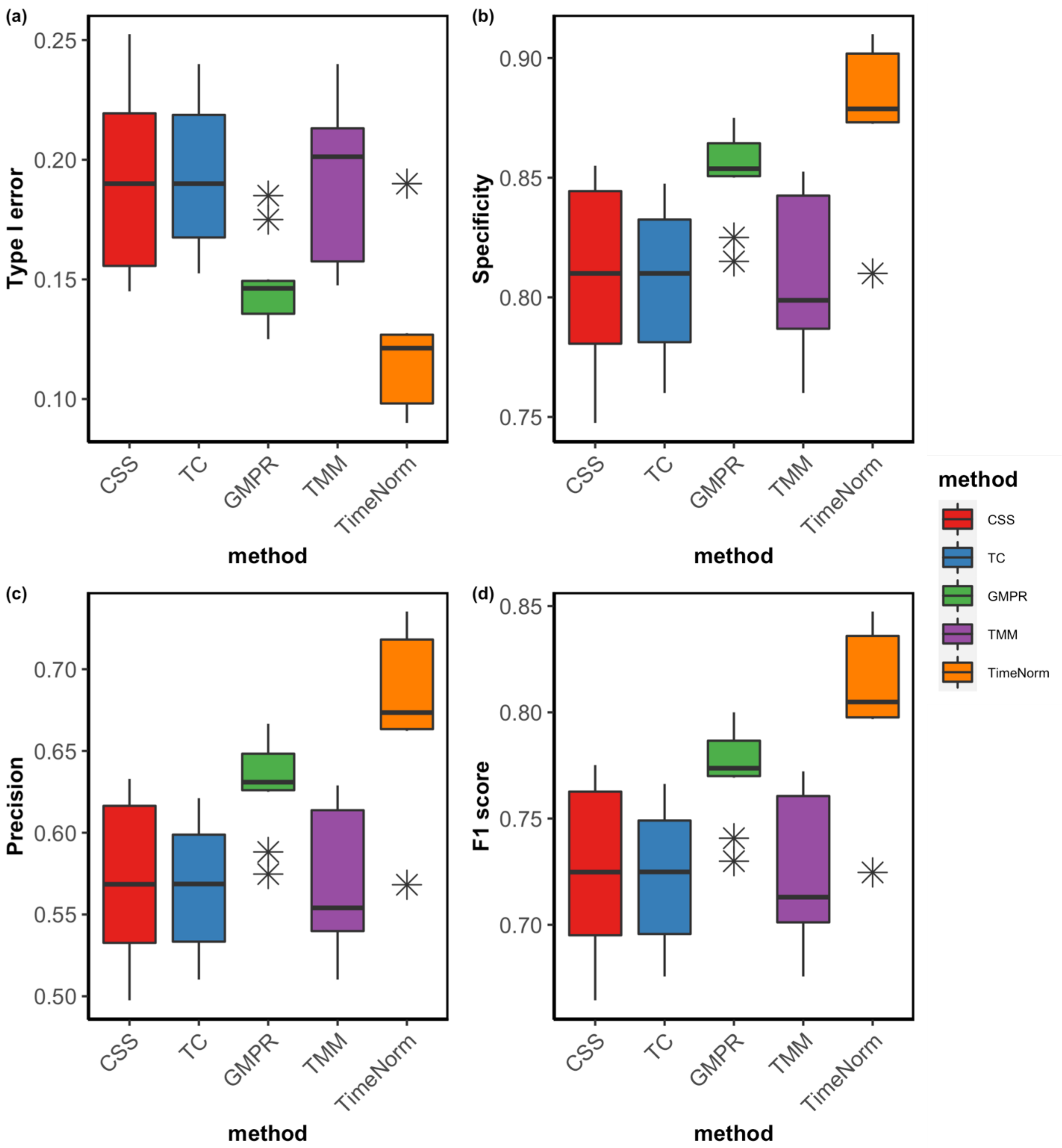

S48: Boxplots of Type I error, Specificity, Precision, F1 score of DA analysis using splinectomeR for different normalization methods based on ten replicated simulations for **Test 4A**.

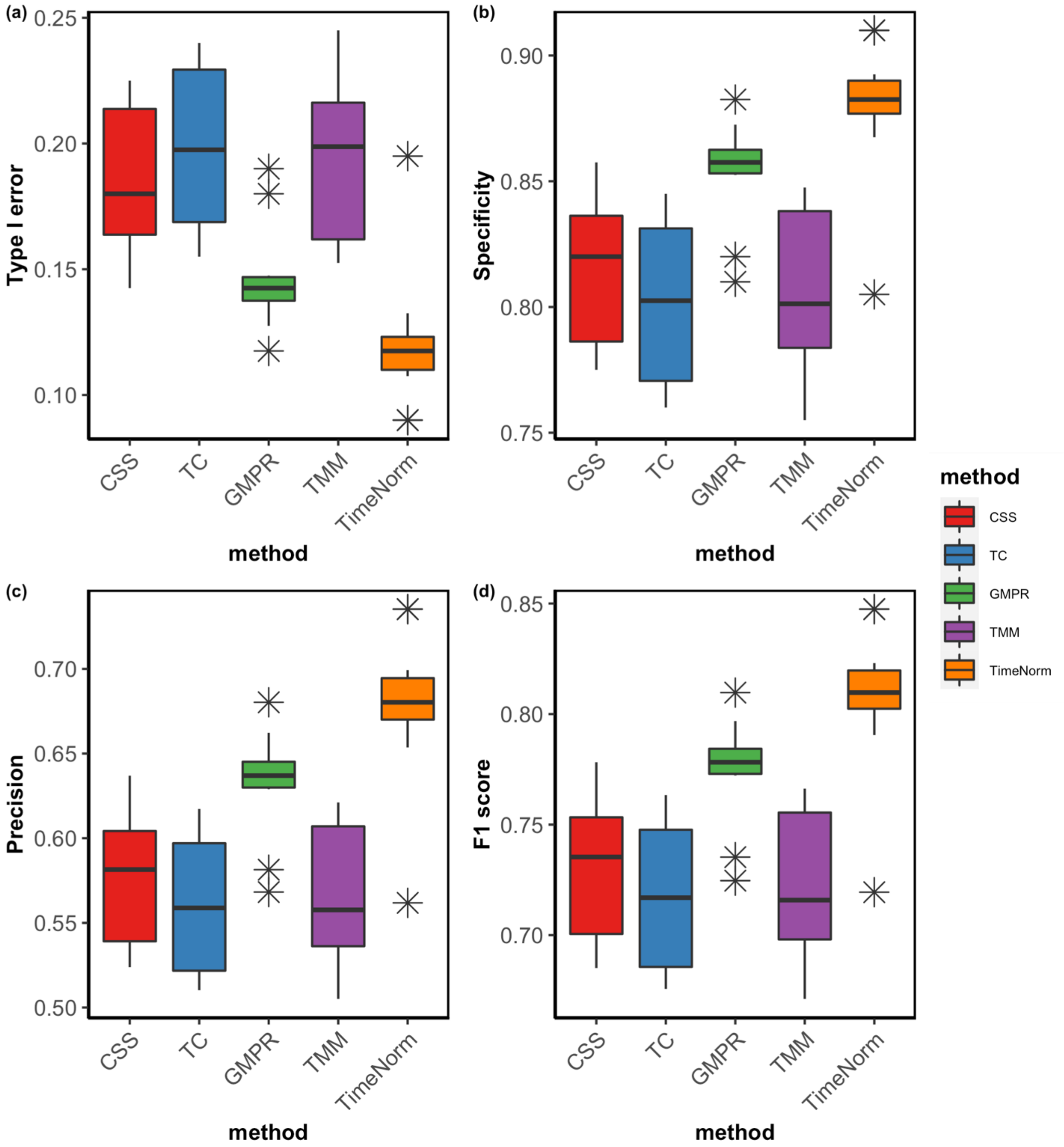

S49: Boxplots of Type I error, Specificity, Precision, F1 score of DA analysis using splinectomeR for different normalization methods based on ten replicated simulations for **Test 4B**.

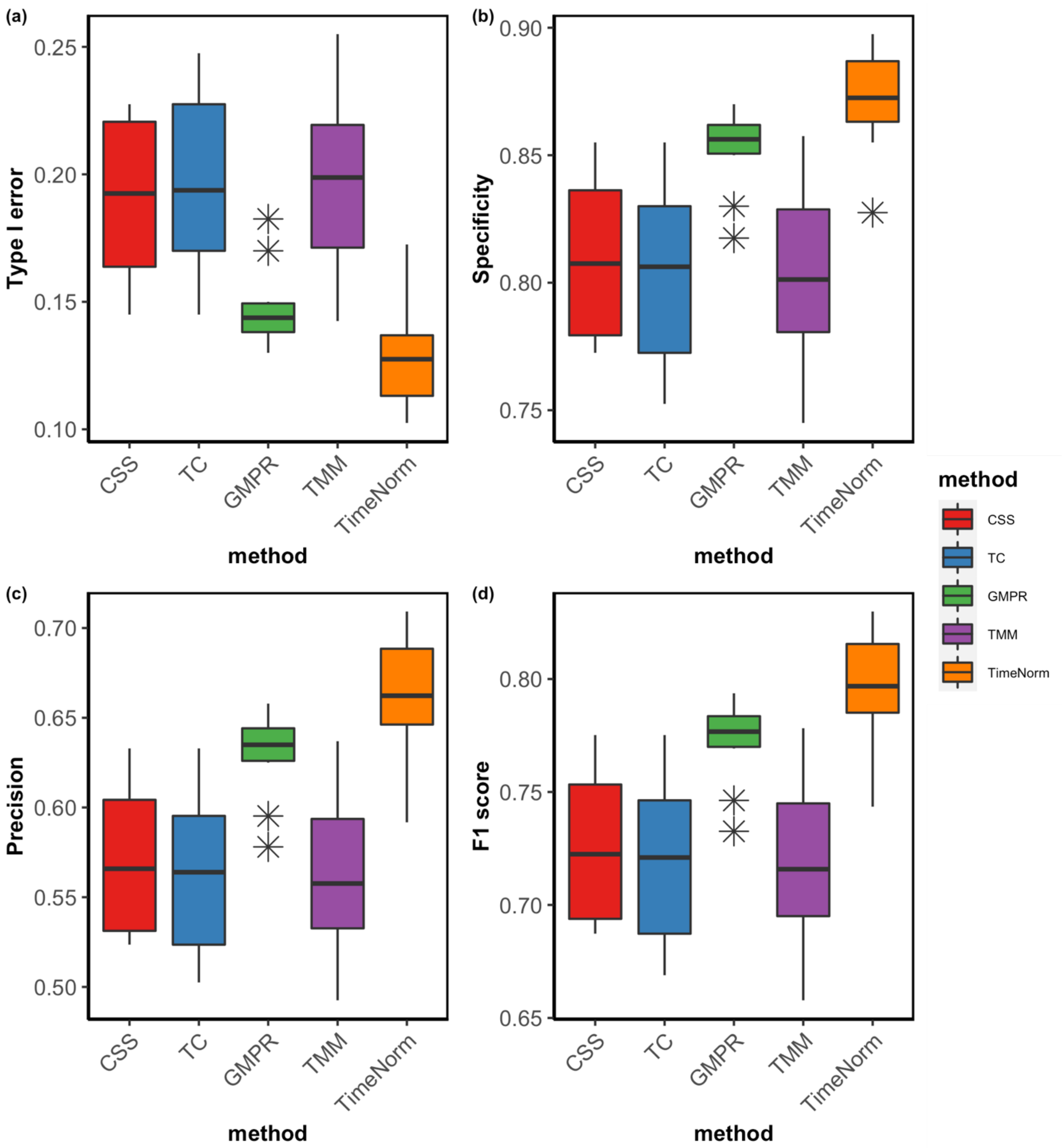

S50: Boxplots of Type I error, Specificity, Precision, F1 score of DA analysis using splinectomeR for different normalization methods based on ten replicated simulations for **Test 4C**.

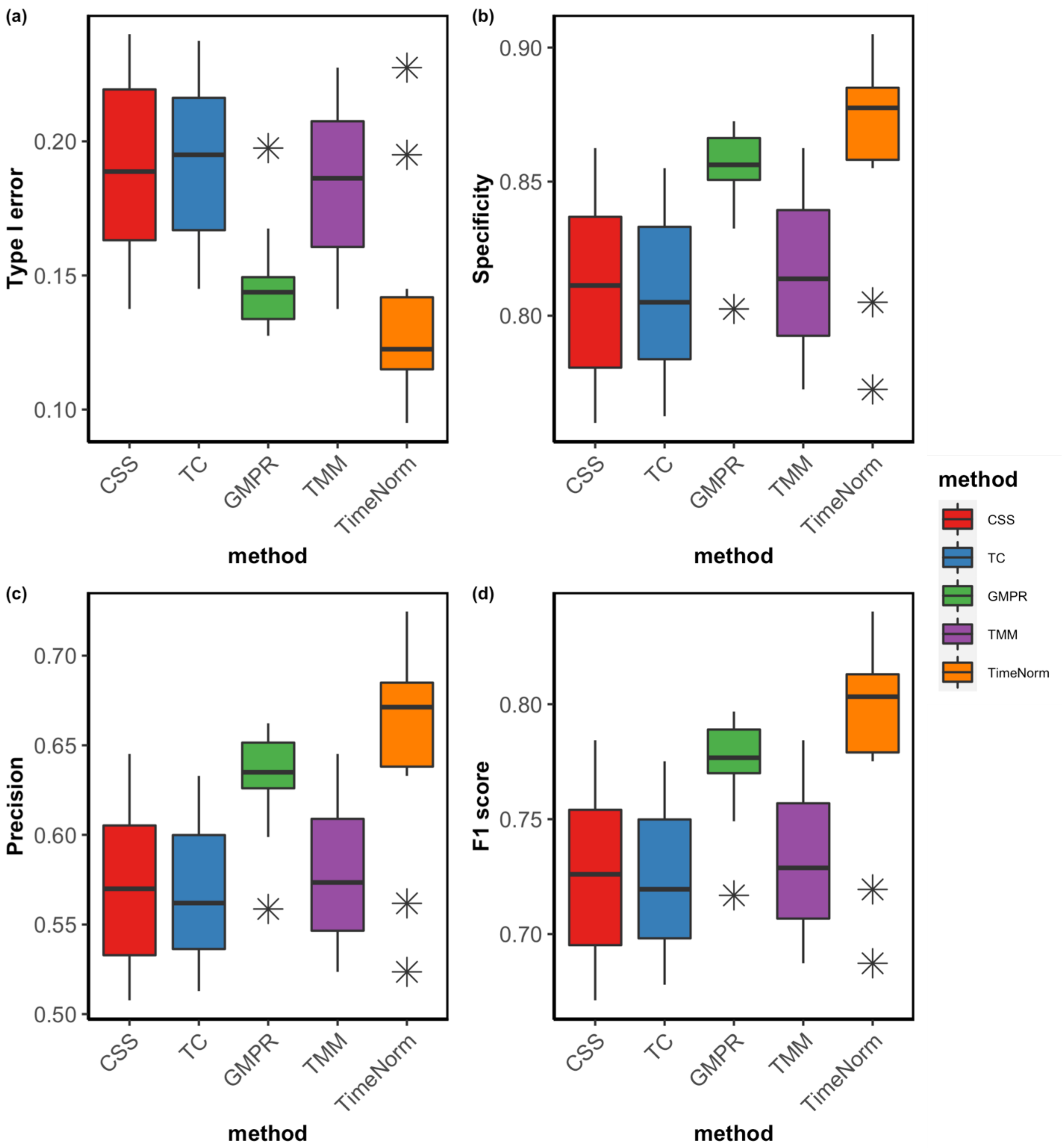

S51: Boxplots of Type I error, Specificity, Precision, F1 score of DA analysis using splinectomeR for different normalization methods based on ten replicated simulations for **Test 4D**.

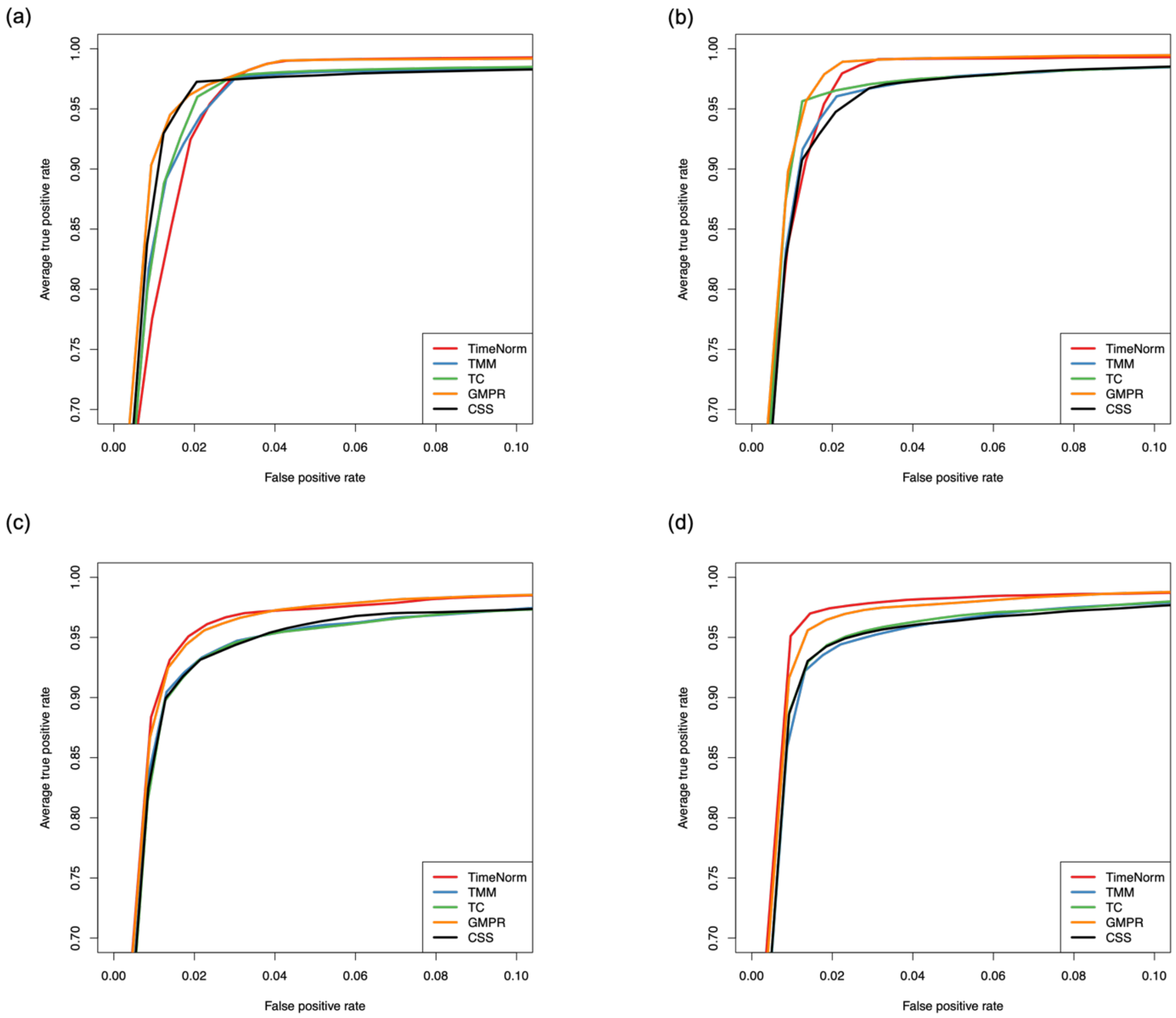

S52: Partial of mean Receiver Operating Characteristic (ROC) for various settings based on ten replicated simulations with 500 features. (a)Test 1A; (b)Test 2A; (c)Test 3A; and (d)Test 4A.

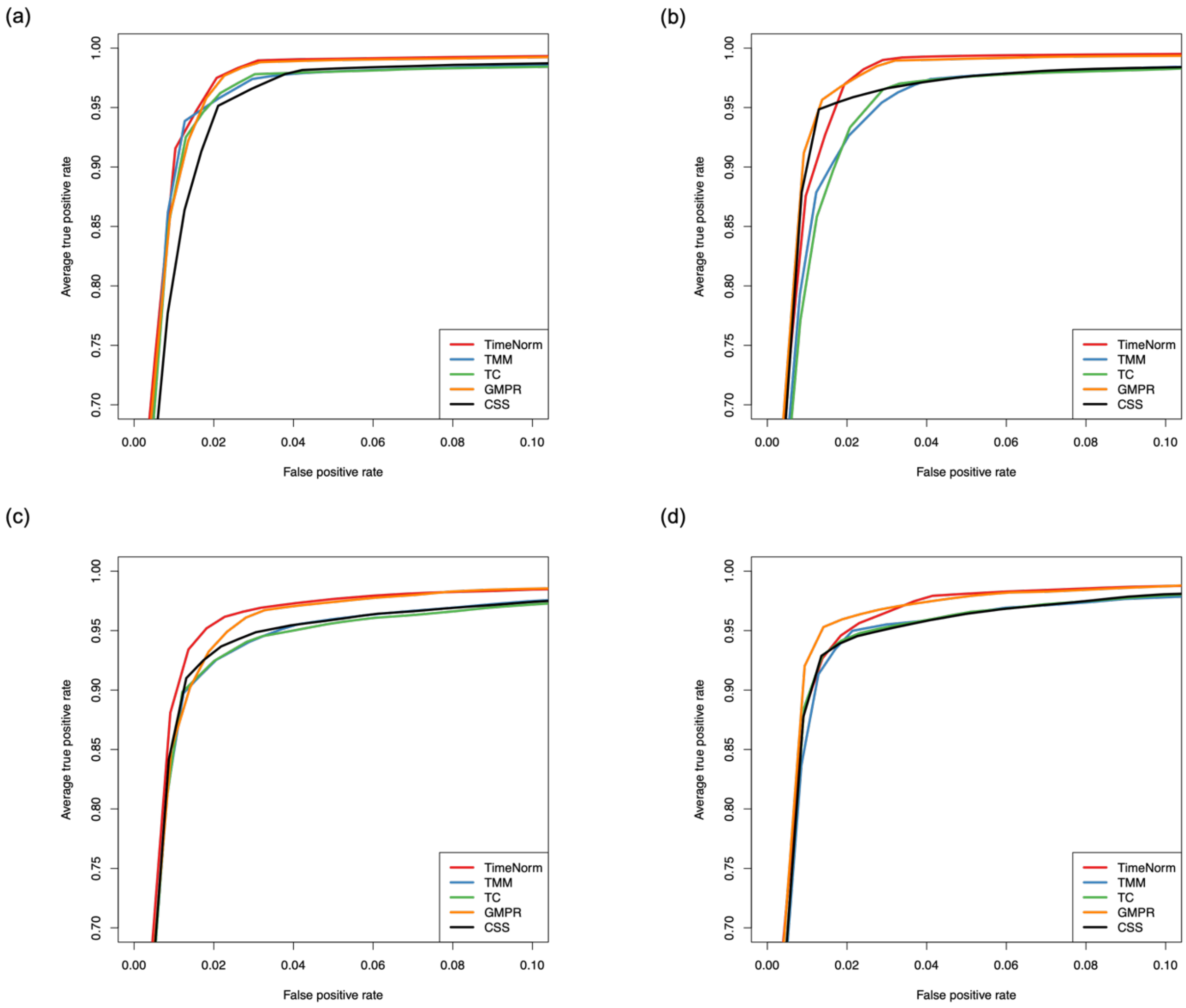

S52: Partial of mean Receiver Operating Characteristic (ROC) for various settings based on ten replicated simulations with 500 features. (a)Test 1B; (b)Test 2B; (c)Test 3B; and (d)Test 4B.

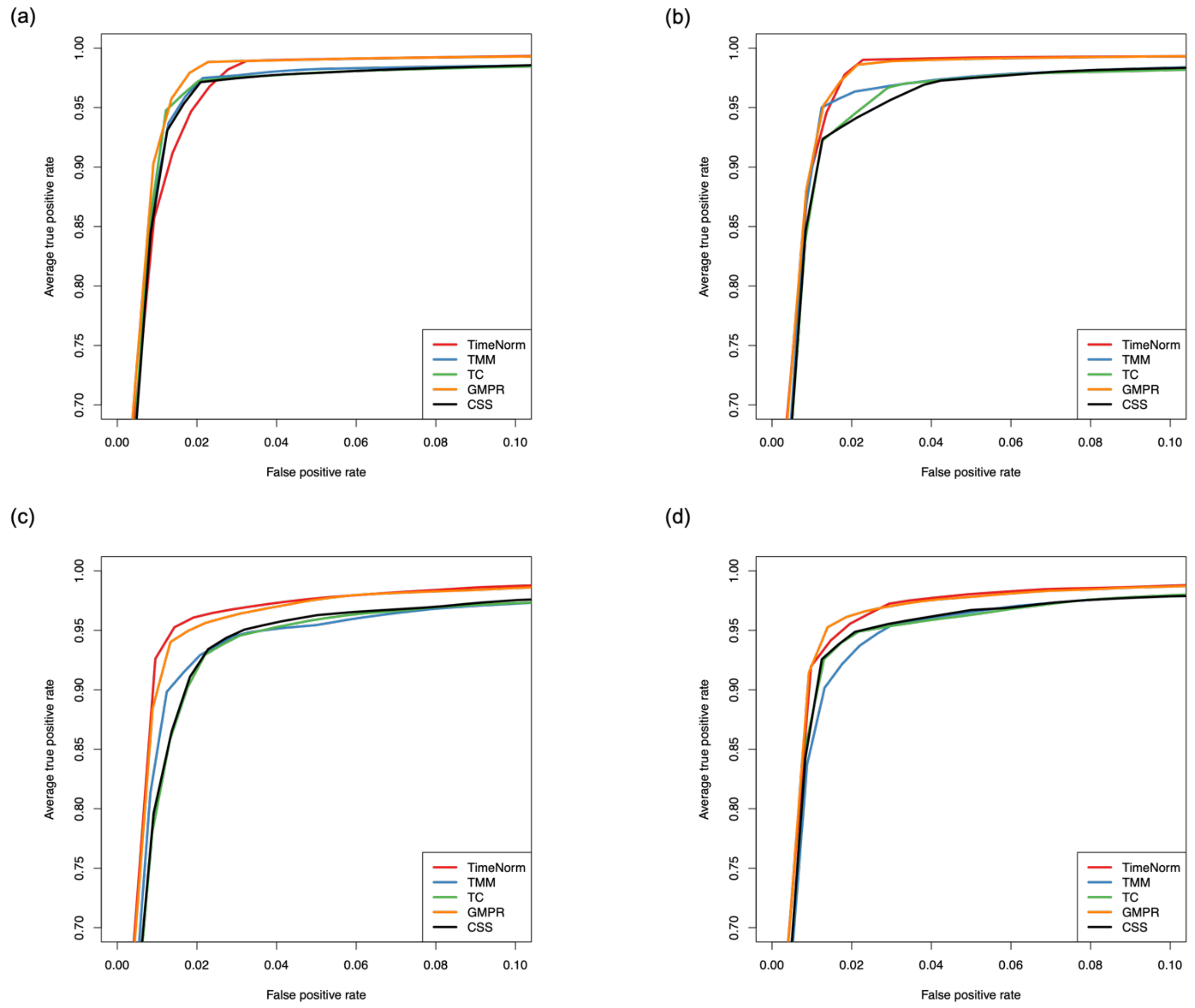

S53: Partial of mean Receiver Operating Characteristic (ROC) for various settings based on ten replicated simulations with 500 features. (a)Test 1C; (b)Test 2C; (c)Test 3C; and (d)Test 4C.

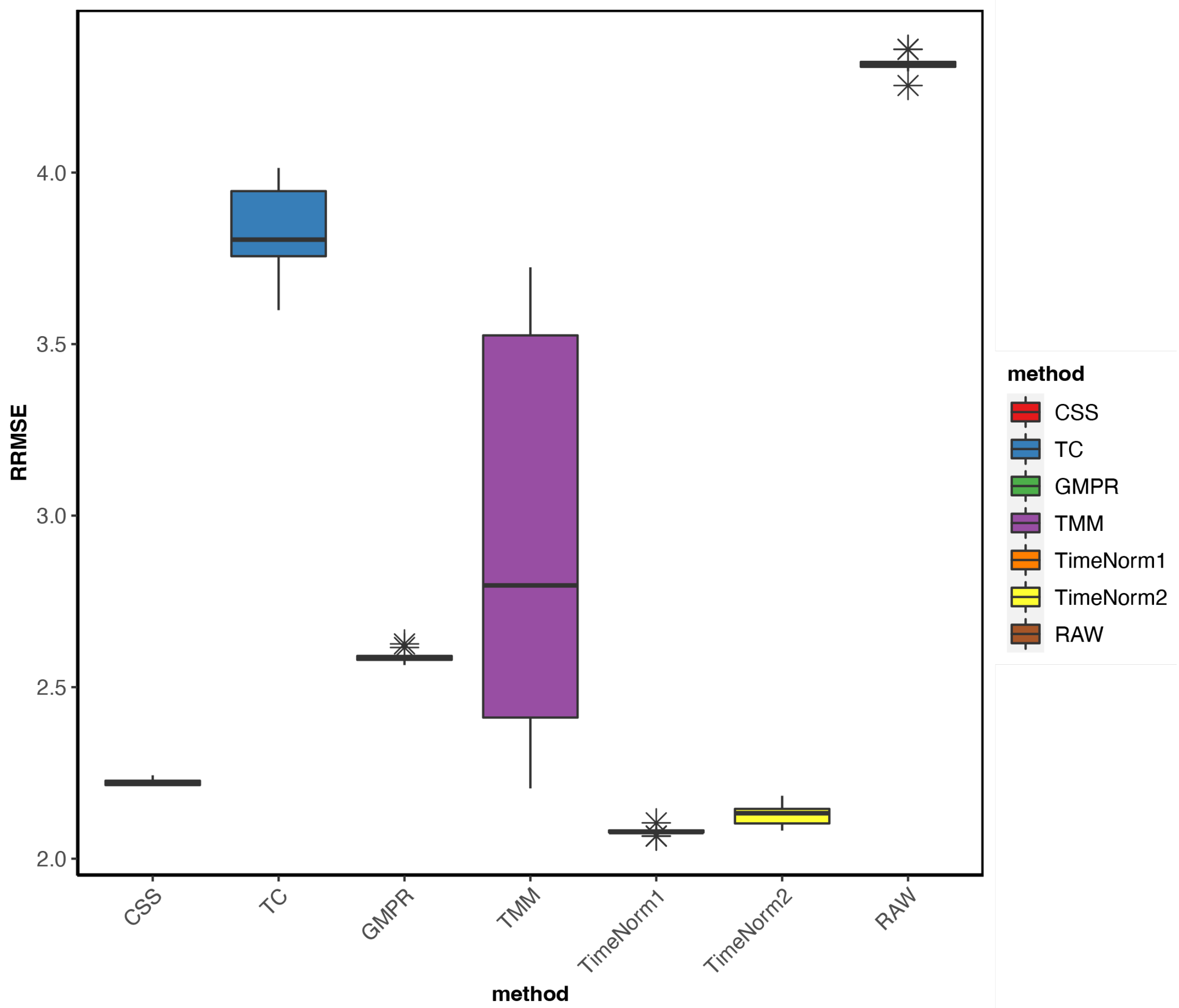

S54: Boxplots of Relative Root Mean Square Error for comparison of different normalization methods based on ten replicated simulations for Test 2D. TimeNorm1 use the minimum number of different features as stable features. TimeNorm2 use the second least number of different features as stable features. The short error bars represent the standard deviation from ten replications.
